# Revealing the key point of the temperature stress response of *Arthrospira platensis* C1 at the interconnection of C- and N- metabolism by proteome analyses and PPI networking

**DOI:** 10.1186/s12860-020-00285-y

**Published:** 2020-06-12

**Authors:** Pavinee Kurdrid, Phutnichar Phuengcharoen, Jittisak Senachak, Sirilak Saree, Apiradee Hongsthong

**Affiliations:** 1 Biochemical Engineering and Systems Biology Research Group, National Center for Genetic Engineering and Biotechnology, National Science and Technology Development Agency at King Mongkut’s University of Technology Thonburi, Mailing Address: IBEG/BIOTEC@KMUTT, 49 Soi Thian Thale 25, Tha Kham, Bang Khun Thian, Bangkok, 10150 Thailand; 2 Pilot Plant Development and Training Institute, King Mongkut’s University of Technology Thonburi, 49 Soi Thian Thale 25, Tha Kham, Bang Khun Thian, Bangkok, 10150 Thailand

**Keywords:** Proteome, Phosphoproteome, Protein-protein interaction network, Two-component system, Regulation

## Abstract

**Background:**

Growth-temperature stress causes biochemical changes in the cells and reduction of biomass yield. Quantitative proteome of *Arthrospira platensis* C1 in response to low- and high temperature stresses was previously analysed to elucidate the stress response mechanism. The data highlighted the linkage of signaling proteins and proteins involved in nitrogen and ammonia assimilation, photosynthesis and oxidative stress.

**Results:**

After phosphoproteome analysis was carried out in this study, the tentative temperature response cascade of *A. platensis* C1 was drawn based on data integration of quantitative proteome and phosphoproteome analysis and protein-protein interaction (PPI) networks. The integration revealed 31 proteins regulated at the protein-expression and post-translational levels; thus, this group of proteins was designated bi-level regulated proteins. PPI networks were then constructed based on *A. platensis* C1 gene inference from publicly available interaction data. The key two-component system (TCS) proteins, SPLC1_S082010 and SPLC1_S230960, were identified as bi-level regulated proteins and were linked to SPLC1_S270380 or glutamate synthase, an important enzyme in nitrogen assimilation that synthesizes glutamate from 2-oxoglutarate, which is known as the signal compound that regulates the carbon/nitrogen (C/N) balance of cells. Moreover, the role of the p-site in the PPIs of some phosphoproteins of interest was determined using site-directed mutagenesis and a yeast two-hybrid system. Evidence showing the critical role of the p-site in the PPI was observed for the multi-sensor histidine kinase SPLC1_S041070 (Hik28) and glutamate synthase. PPI subnetwork also showed that the Hik28 involved with the enzymes in fatty acid desaturation and nitrogen metabolism. The effect of Hik28-deletion was validated by fatty acid analysis and measurement of photosynthetic activity under nitrogen depletion.

**Conclusions:**

Taken together, the data clearly represents *(i)* the multi-level regulation of proteins involved in the stress response mechanism and *(ii)* the key point of the temperature stress response at the interconnection of C- and N- metabolism.

## Background

The elevation and reduction of growth temperature are important environmental factors for cell viability, growth and capacity to economically synthesize high-value chemicals. Low-temperature stress leads to impaired protein biosynthesis, the stabilization of DNA and RNA secondary structures, and, particularly, a reduction in membrane fluidity, whereas high-temperature stresses causes protein aggregation and denaturation; to cope with these stresses, a cellular response occurs. Thus, various stress response mechanisms are well evolved to adapt to physical and biochemical changes in the cellular microenvironment. In *Spirulina* (*Arthrospira*), a cyanobacterium, when the cells are mass cultivated in outdoor ponds, temperature fluctuations are typically encountered, causing relevant effects on the biomass yield and the biochemical content of the cells, which can have economic value due to pharmaceutical properties, e.g., in the case of polyunsaturated fatty acids. Cells undergo many cellular modifications under thermal stress conditions, and these modifications are generated by a network of genes regulated either simultaneously or in a cascade.

Phosphorylation is one of the most biologically relevant post-translational modifications and plays a crucial role in many cellular mechanisms, including signal transduction in response to various stresses. Protein phosphorylation-based signaling is regulated by a reversible enzyme-catalyzed process, in which protein kinases and protein phosphatases participate in an antagonistic manner. Several studies have suggested that Ser/Thr/Tyr phosphorylation plays a key role in the regulation of cellular responses and physiological processes in prokaryotes [1, 2]. In cyanobacteria, a two-component system comprised of a histidine kinase and its specific response regulator functions by a phosphorylation-based signal transduction mechanism [3]. Therefore, the signal transduction cascade through the phosphorylation of serine, threonine and tyrosine residues in response to stresses involves both a two-component system and Ser/Thr/Tyr kinases [3, 4]. Moreover, in response to a signal, the expression of many corresponding genes will either be induced or reduced for cellular adaptation to a particular stress. Gene regulation at the transcriptional level via promoter recognition by a sigma factor is a cellular process tightly associated with phosphorylation [5, 6].

After the *Spirulina (Arthrospira) platensis* C1 genome was completely analyzed and published [7], high-throughput approaches, including gel-based and non-gel-based proteomics, were applied in previous studies to analyze the temperature response of this organism [8–10]. Using a bioinformatics approach, the results of comparative proteome analyses of low- and high-temperature stresses and potential protein-protein interaction networks in response to both stress conditions have led to four important developments [10]. First, the data indicate that proteins involved in nitrogen and ammonia assimilation are associated with temperature stress responses. Second, low-temperature stress is tightly linked with photosynthesis and oxidative stress; however, no specific mechanism has been revealed in the high-temperature stress response. Third, the results highlight the crosstalk between signaling pathways, e.g., Hik14, Hik21 and Hik28, revealing potential interactions between differentially expressed proteins identified in both temperature stress conditions. Finally, a yeast two-hybrid system (Y2H) was employed to examine differentially expressed proteins identified in the *Spirulina* protein-protein interaction network, and the results suggested that the potential protein-protein interaction (PPI) network may generate interactions in *Spirulina*. Therefore, in contrast to proteome analyses of knockout mutants, the bioinformatics approach used in the analysis of the protein-protein interaction network helped to directly examine the specificity or crosstalk between signaling components and their target proteins.

Previously, the phosphoproteomes produced in response to heat and cold stresses were investigated in *Saccharomyces cerevisiae* [11]. However, the present study is the first to use a phosphoproteomics strategy to identify phosphorylated proteins under low- and high-temperature conditions to analyze temperature stress response mechanisms in cyanobacteria, which are photosynthetic organisms. Moreover, comparative analyses of the phosphoproteome profile under both temperature stresses were also performed. In addition to the comparative phosphoproteomics analysis, an integrated potential PPI network of phosphorylated proteins and differentially expressed proteins identified in previous studies was constructed using a bioinformatics approach to determine the biological significance of the proteomics data. PPI pairs involved in signal transduction mechanisms and presented in the bi-level regulated network were examined for their potential roles in phosphorylation using a yeast two-hybrid system (Y2H). Moreover, the possible role of the protein in signal transduction mechanism, Hik28, involved in fatty acid desaturation and nitrogen metabolism suggested by the PPI subnetwork was also validated by fatty acid analysis and determination of photosynthetic activity of its deletion mutant under nitrogen-depleted condition.

## Results

### General overview of the quantitative proteome and phosphoproteome data

The *Arthrospira (Spirulina) platensis* strain C1 genome contains 6108 ORFs, among which 2983, 10 and 4 proteins were hypothetical, of unknown function and uncharacterized proteins, respectively [7]. In a previous *A. platensis* C1 proteome coverage study, 3434 and 1370 proteins (a total of 4804 proteins) were identified using gel- and non-gel-based techniques, respectively [10].

**Fig. 1:**
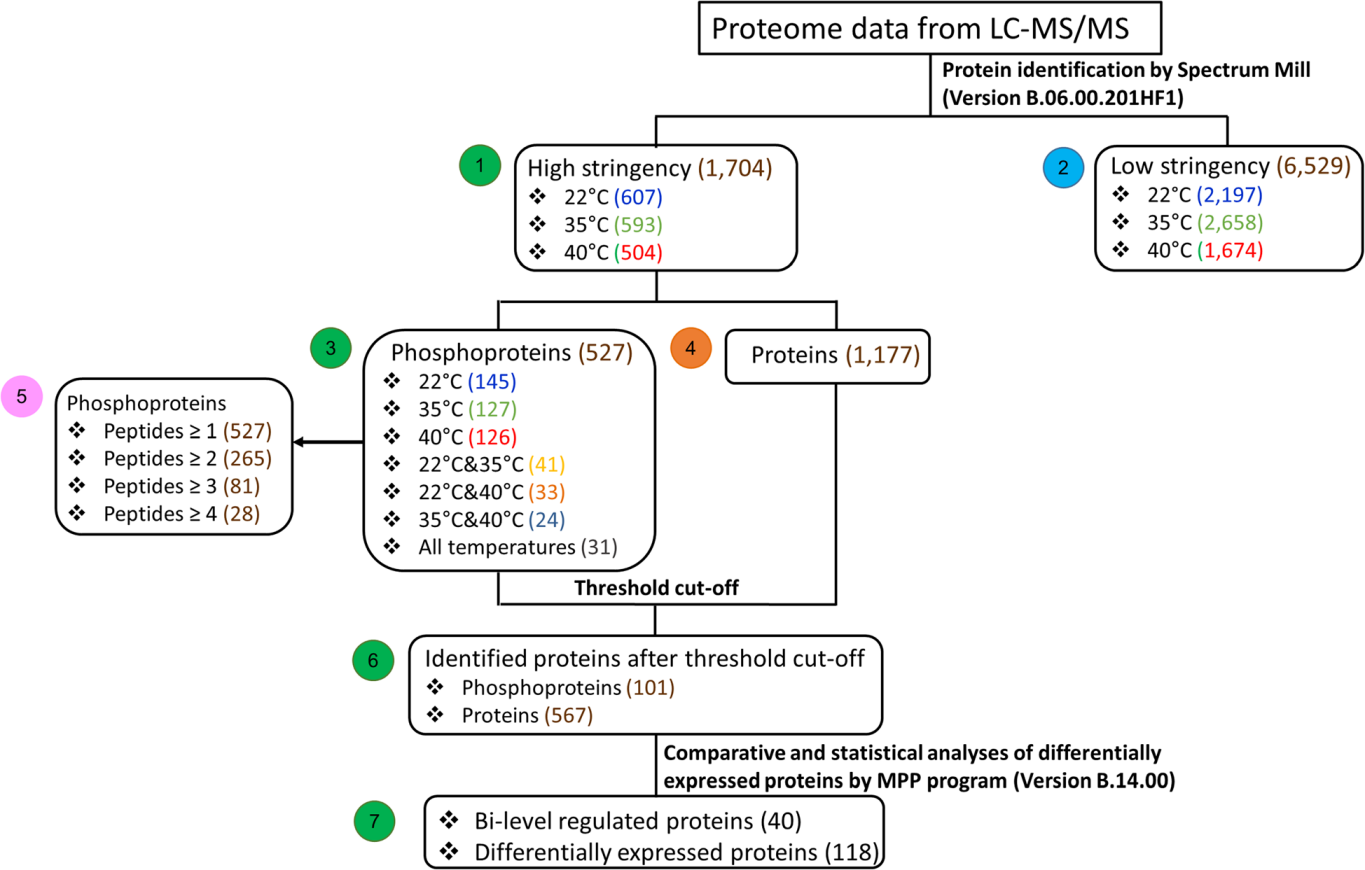
Workflow for the quantitative and phosphoproteome data analyses. The number in parenthesis is the number of proteins in each group. The number in color-circle indicates the number of the each data-box

In the present study, comparative analyses of the quantitative proteome and phosphoproteome of *A. platensis* C1 grown under optimal temperature and after temperature shifts to 22 °C and 40 °C for 180 min were carried out. The data obtained from LC-MS/MS was analyzed according to the flowchart in Fig. 1. The total number of identified proteins after the threshold cut-off was 668 proteins, consisting of 101 phosphoproteins and 567 differentially expressed proteins (Box 6, Fig. 1). Then, the proteins regulated at the translational and post-translational levels or designated bi-level regulated proteins were identified (Box 7, Fig. 1). The details on each group of proteins in Fig. 1 are discussed in the following sections.

### Quantitative proteome analysis: expression patterns of differentially expressed proteins and their protein-protein interaction networking

**Fig. 2:**
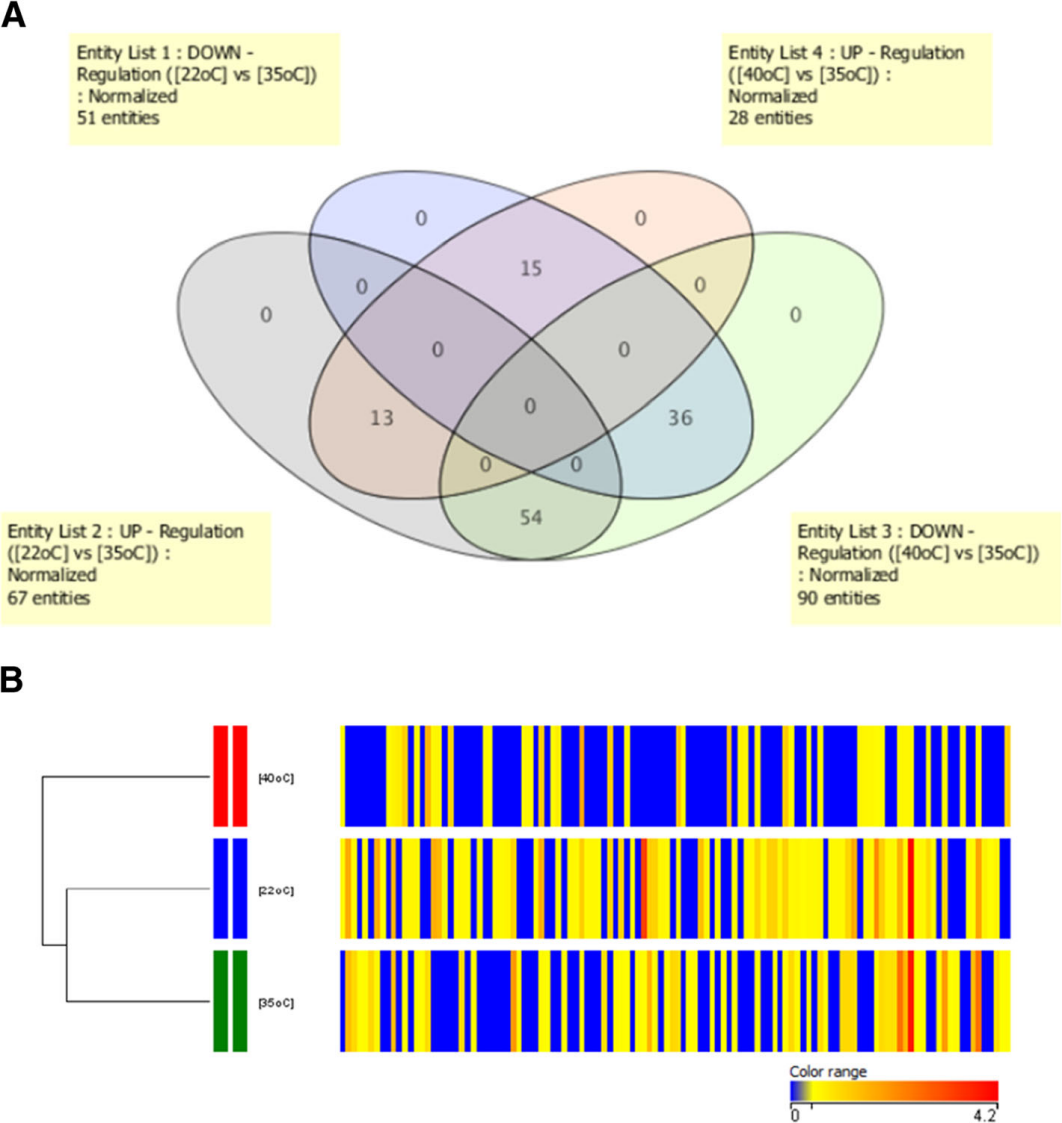
Protein expression level obtained from quantitative proteome analysis; (**a**) Venn diagram showing the numbers of up- and down- regulated proteins at 180 min after the temperature downshift, 22 °C, (Purple and Blue objects) and upshift, 40 °C, (Pink and Green objects) and (**b**) Heatmap hierarchical clustering of the protein expression under the three experimental conditions

In our previous studies, the differentially expressed proteins obtained from proteome analysis of samples collected from different time points were clustered by their expression patterns as adaptive, tolerant and resistive responses [10, 12]. The time point at 180 min after the temperature shift showed saturation of the number of differentially expressed proteins. Therefore, in the present study, wherein comparative analysis of the quantitative proteome and phosphoproteome was carried out to identify the bi-level regulated proteins, the fold-changes of protein expressed levels at 180 min after low- and high-temperature stresses were analyzed. Sixty-seven and twenty-eight proteins were found to be upregulated after temperature shifts to 22 °C and 40 °C, respectively, whereas 51 and 90 proteins were downregulated after the upshift and downshift of the temperature (Fig. 2a). The heatmap hierarchical clustering of the protein expression pattern showed the association of the pattern under the optimal growth condition and under low temperature stress (Fig. 2b).

Upon comparative analysis of the protein regulation at 22 °C and 40 °C, the proteins could be grouped according to their regulation under both temperatures. There were 17 and 35 proteins which were up- and downregulated under both stress temperatures, respectively. Moreover, two groups of proteins showed diverse directions of regulation after temperature upshift and downshift. The group of 49 proteins was upregulated at 22 °C and downregulated at 40 °C, whereas the other 17 proteins were downregulated at low temperature and upregulated at high temperature (see Additional file 1). Then, the PPI subnetwork (see Additional file 2A-D) of each group was generated using STRING, and the *A. platensis* C1 proteins were inferred using *A. platensis* NIES39 via orthologous grouping because *A. platensis* C1 proteins are not present in the STRING database. The pathway analysis showed the connection of each group of proteins with various pathways (Table 1), and it should be noted that the proteins in nitrogen metabolism were upregulated at 22 °C and vice versa at 40 °C. In contrast, the proteins involved in carbon fixation and carbon metabolism were downregulated under both stress temperatures.

**Table 1:** List of metabolic pathways which the proteins in the PPI networks of up- and down- regulated proteins were involved

Pathway/Protein regulation level at 22 °C - 40 °C			
down - down	up - up	down - up	up - down
Metabolic pathways	Metabolic pathways	Metabolic pathways	Ribosome
Ribosome	Oxidative phosphorylation	Oxidative phosphorylation	Nitrogen metabolism
Carbon fixation in photosynthetic organisms	Photosynthesis	Photosynthesis	
Oxidative phosphorylation	Purine metabolism		
Biosynthesis of amino acids	Alanine, aspartate and glutamate metabolism		
Photosynthesis - antenna proteins	Aminoacyl-tRNA biosynthesis		
Microbial metabolism in diverse environments			
Carbon metabolism			
2-Oxocarboxylic acid metabolism			
Nicotinate and nicotinamide metabolism			
Valine, leucine and isoleucine biosynthesis			
Biosynthesis of secondary metabolites			

### Phosphoproteome analysis: phosphorylation patterns, protein-protein interaction networks and data validations

#### Phosphorylation patterns

There were 527 phosphoproteins that passed the high-stringency criteria cut-off, as shown in Box 3 of Fig. 1. The ratio of pS/pT/pY was 50.7%:40.1%:9.2%, which is different from that of *Synechocystis sp.* PCC6803 and *Synechococcus sp.* PCC7002 (42.3%:51.5%:6.2%). Phosphoserine was the majority p-site in *A. platensis* C1. After threshold filtering, 101 phosphoproteins were identified (Box 6, Fig. 1 and Additional file 3), and the ratio of the three p-sites was similar to that obtained after the high-stringency cut-off. The MS/MS spectra of some peptides of interest are shown in Additional file 4.

According to the phosphoproteome data (Box 3, Fig. 1) of *A. platensis* C1, eight phosphorylated Ser/Thr kinases were detected in their phosphorylated form. SPLC1_S032990 was the only Ser/Thr kinase after the threshold cut-off (Box 6 of Fig. 1). Notably, *(i)* dephosphorylation of the two p-sites of SPLC1_S032990 occurred upon a temperature upshift (see Additional file 5), and *(ii)* none of Ser/Thr kinases identified in the present study had orthologs in *Synechocystis* sp. PCC6803, for which the signal transduction systems under abiotic stresses have been extensively studied.

#### Protein-protein interaction networks

The PPI subnetwork of the 101 phosphoproteins was constructed using STRING (see Additional file 6). The network represents the connections of proteins involved in several pathways, and some, i.e., nucleotide excision repair, mismatch repair, glycine, serine and threonine metabolism, and selenocompound metabolism, were different from those of the group of differentially expressed proteins.

Among these proteins, three histidine kinases, i.e., SPLC1_S041070, SPLC1_S540750 and SPLC1_S081620, were found (see Additional file 3). SPLC1_S041070 or Hik28 contains eight p-sites, pT598, pT793, pT837, pS805, pS911, pS976, pS1136 and pS1482, all of which were detected only under low temperature. The PPI subnetwork of Hik28, NIES39_D04330, was constructed using STRING (see Additional file 6). It revealed the association of this low-temperature responsive Hik with other key proteins, i.e., several TCS proteins, response regulator receiver/diguanylate cyclase (PleD), serine/threonine kinase (SPLC1_S240280) and proteins in nitrogen assimilation. Moreover, the PPI subnetwork of the SPLC1_S041070-orthologous protein, Sll0474, in *Synechocystis* sp. PCC6803 presented its association with the proteins involved in fatty acid desaturation and N-metabolism (see Additional file 7).

#### Bi-level regulated proteins and their protein-protein interaction (PPI) networking

**Fig. 3:**
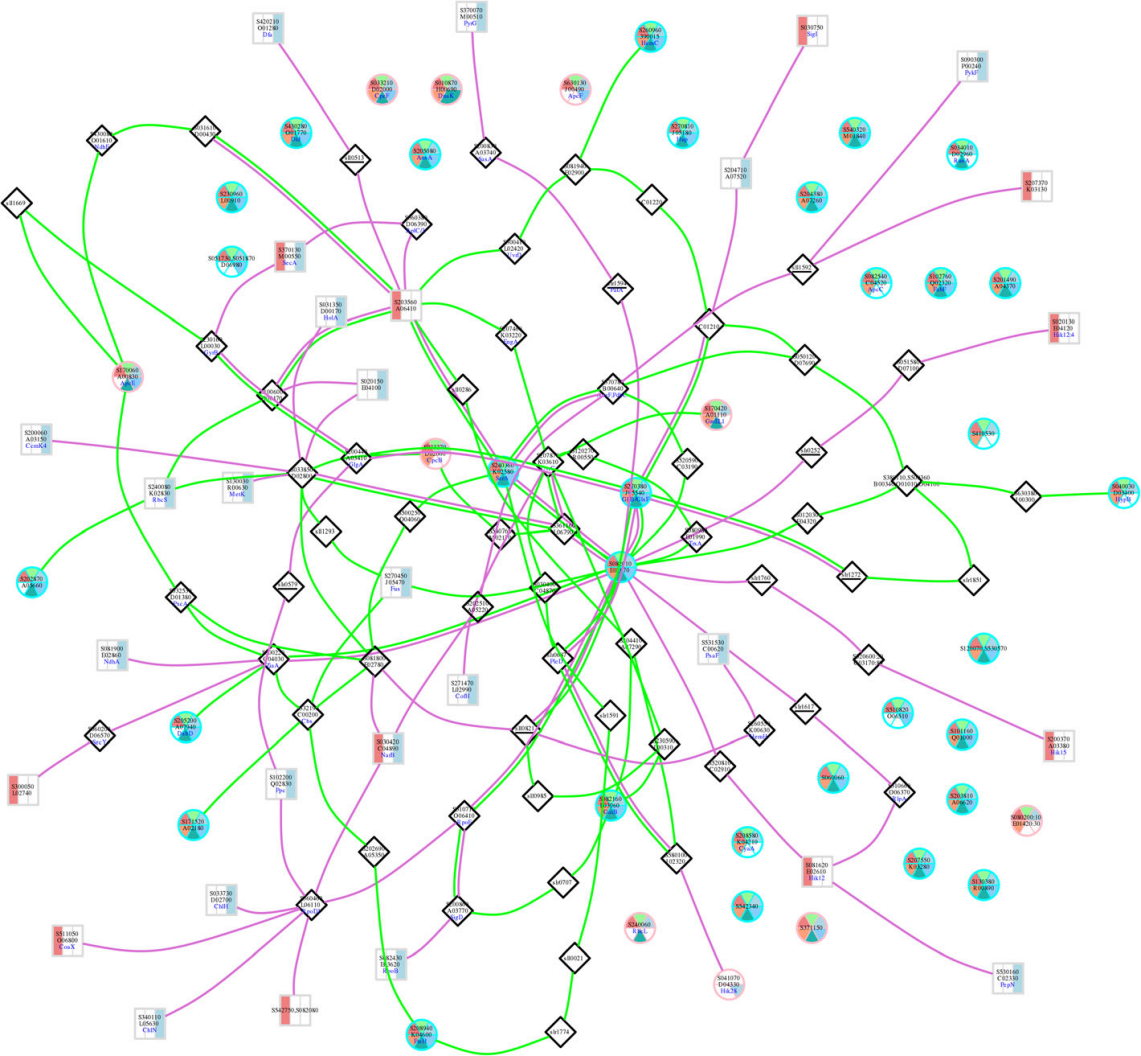
PPI network of bi-level regulated proteins constructed using an in-house tool. *Nodes:* The proteins with six-sector circle-shaped nodes were bi-level regulated proteins. In the circle nodes, the upper three sectors of the total six sectors represent the temperature condition that the protein was identified in the quantitative proteome study (from left to right; pink for high, light green for optimal, and blue for low temperature), and the lower three represent the temperature condition that the protein was identified in the phosphoproteome study (from left to right; orange for high, green for optimal, and cyan for low temperature). The cyan-bordered circle nodes indicate the up- or down regulated proteins with the expression level ≥ 1.5-fold, whereas the pink ones indicate the up- or down regulated proteins with the expression level < 1.5-fold compared to that of under the optimal condition. In the grey-bordered rectangular nodes, the leftmost pink strip indicates high temperature, and the rightmost blue strip indicates low temperature. All other proteins are indicated in black-bordered diamond-shaped nodes. *Edges:* The green edges indicate interaction-paths between bi-level regulated proteins (circle nodes), whereas the purple edges indicate interaction-paths between bi-level regulated proteins and differentially expressed proteins (rectangle nodes). *Note:* The protein SPLC1_S230960 does not have orthologous in *Synechocystis sp.* that is the template for PPI network construction of the in-house tool. Thus, there were no edges around its node, which is different from PPI network constructed by using STRING (see Additional file 12)

The 40 proteins (Box 7, Fig. 1) regulated at the protein expression and post-translational levels were identified. Among the 40 phosphorylated proteins, there were 31 proteins that were significantly up- or down regulated more than or equal to 1.5-fold in response to stress. The PPI subnetworks of these proteins were constructed using SVG graphics on the *Synechocystis* template validated by Y2H (Fig. 3) and STRING (see Additional file 8) [13, 14]. In Fig. 3, the network illustrates the direct interaction between a bi-level regulated protein, GlsF, and a multi-sensor hybrid histidine kinase (Hik), NIES39_D04330, in the same orthologous group as SPLC1_S041070 or Hik28 of *A. platensis* C1.

#### Data validation of *A. platensis* C1-Hik28, SPLC1_S041070, by site-directed mutagenesis and yeast two hybrid system

To validate the role in protein binding of Hik28, the Y2H technique was applied for the binding study of the wild-type (WT)- SPLC1_S041070, Hik28, and the site-directed-mutant S976A- SPLC1_S041070 with their possible client proteins, TCS proteins, PleD, Ser/Thr kinase and GlsF suggested by the STRING database (Table 2 and see Additional file 9). The positive results were observed between the WT-Hik28 and the client proteins, whereas the negative results were shown in case of the S976A-mutant and the clients. It is worth noting that the two TCS proteins in the PPI subnetwork, SPLC1_S540750 (Hik21) and SPLC1_S360070, also showed interaction with the response regulator, PleD (see Additional file 9).

**Table 2:**
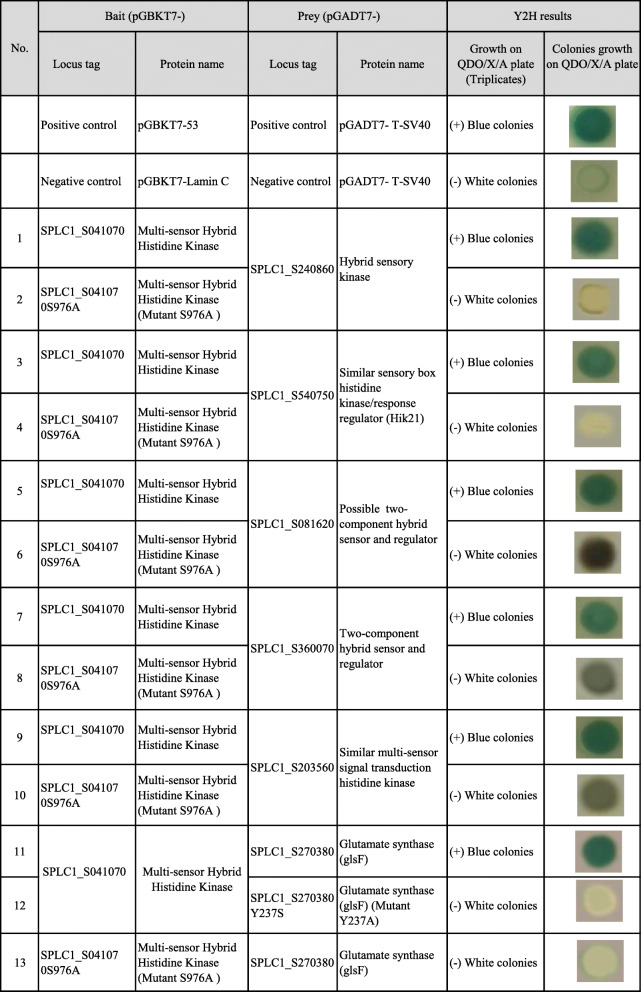
Protein-protein interaction analyses of the proteins of interest by using yeast two-hybrid system (Y2H)

#### Data validation of Hik28 by its orthologous gene deletion and analyses of fatty acid and photosynthetic activity in Synechocystis sp. PCC6803

To elucidate the possible role of SPLC1_S041070 or Hik28, the deletion mutant of its orthologous protein, Sll0474, was constructed in *Synechocystis* sp. PCC6803 (see Additional file 10) due to *(i)* the specific gene transfer is unavailable in *A. platensis* and *(ii)* the ortholog of SPLC1_S041070 is absent in the other model cyanobacteria, *Synechococcus* sp. PCC 7942.

After the fatty acid analysis of the wild type *Synechocystis* sp. PCC6803 and the Δsll0474 mutant grown under the three experimental temperatures, the data showed the accumulation of 16:1^Δ9^ in the mutant strain (Table 3 and Additional file 11). When compared between the two strains, the composition of 16:1^Δ9^ was significantly (*p* < 0.05) increased in the mutant, whereas, the change of the 16:1^Δ9^ composition in the mutant was temperature independent (*p* > 0.05) (Table 3).

**Table 3:** Fatty acid analysis of *Synechocystis* sp. PCC6803 WT and Δsll0474 mutant by using gas chromatography

Strain	Fatty acid (% Composition)							
	C16:0	C16:1	C18:0	C18:1 (Δ9)	C18:2 (Δ9,12)	C18:3 (Δ6,9,12)	C18:3 (Δ9,12,15)	C18:4 (Δ6,9,12,15)
**Cells grown at 30 °C**								
WT	47.7 + 2.4	3.1 + 0.8	1.0 + 0.5	6.8 + 3.0	19.1 + 4.4	18.9 + 2.5	1.9 + 0.09	1.4 + 0.06
MTΔsll0474	49.9 + 1.0	37.3 + 7.8	0.6 + 0.02	2.4 + 0.3	4.1 + 2.9	4.5 + 0.7	0.8 + 0.01	0.5 + 0.01
**Cells grown at 16 °C**								
WT	46.8 + 2.2	3.5 + 1.0	0.9 + 0.4	6.0 + 1.5	19.2 + 4.0	20.6 + 1.3	2.6 + 1.3	0.4 + 0.02
MTΔsll0474	49.9 + 1.1	32.6 + 9.5	0.7 + 0.04	2.4 + 0.1	5.8 + 1.9	6.6 + 1.7	1.3 + 0.05	0.6 + 0.02
**Cells grown at 45 °C**								
WT	48.9 + 2.8	2.9 + 0.6	1.1 + 0.6	7.7 + 3.4	18.9 + 4.1	17.6 + 2.9	1.6 + 0.03	1.2 + 0.04
MTΔsll0474–45	49.4 + 0.6	42.8 + 0.6	0.4 + 0.04	2.4 + 0.2	2.4 + 0.2	2.1 + 0.2	0.2 + 0.02	0.3 + 0.01

In case of photosynthetic activity measurement, the WT and the Δsll0474 cells were grown in the nitrogen depletion media under the optimal temperature and low temperature stress. The chlorophyll content and O_2_ evolution rate represented photosynthetic growth were measured. Their significant reduction after N-depletion in both WT and MT was observed under the optimal temperature, whereas the increasing degree of the reduction was found in the MT strain under the low temperature stress and vice versa in the WT strain (Table 4).

**Table 4:** Chlorophyll *a* content and oxygen evolution rate of *Synechocystis* sp. PCC 6803 WT and Δsll0474 mutant under nitrogen- and low temperature stress

Strain	+NO_**3**_			-NO_**3**_		
	Day0	Day1	Day2	Day0	Day1	Day2
**Cell grown at 30 °C**						
Chlorophyll content (mg/l)						
WT	5.9 + 0.02	6.4 + 0.03	6.69 + 0.01	5.9 + 0.13	5.9 + 0.07	4.91 + 0.01
MTΔsll0474	5.5 + 0.45	5.6 + 0.24	5.87 + 0.05	5.5 + 0.42	5.1 + 0.04	4.13 + 0.04
O_2_ evolution content (μmolO_2_mg^−1^Chl.hr.^− 1^)						
WT	373.1 + 1.6	380.4 + 12.9	390.9 + 2.9	372.6 + 2.3	261.6 + 10.5	252.2 + 6.1
MTΔsll0474	379.1 + 2.2	327.3 + 17.1	381.1 + 12.6	381.2 + 14.8	253.4 + 3.6	244.3 + 6.8
**Cell grown at 16 °C**						
Chlorophyll content (mg/l)						
WT	5.5 + 0.59	5.2 + 0.06	4.80 + 0.14	5.6 + 0.63	4.8 + 0.14	4.00 + 0.14
MTΔsll0474	5.4 + 0.07	4.8 + 0.01	4.22 + 0.03	5.4 + 0.07	4.3 + 0.13	2.30 + 0.12
O_2_ evolution content (μmolO_2_mg^−1^Chl.hr.^−1^)						
WT	368.9 + 24.9	354.5 + 10.7	309.5+ 2.9	368.9 + 24.9	306.3 + 4.9	303.9 + 1.1
MTΔsll0474	379.3 + 20.3	270.7 + 14.3	203.2 + 3.7	378.2 + 6.9	265.6 + 7.1	144.3 + 20.4

#### Data validation of *A. platensis* C1-serine/threonine kinase, SPLC1_S240280, by site-directed mutagenesis and yeast two hybrid system

To examine the role in PPI of pS254 of the Ser/Thr kinase, the Y2H experiments of the WT- and the S254A mutant- Ser/Thr kinase with the histidine kinase, SPLC1_S081620, were carried out. The WT protein showed positive result with the histidine kinase in contrast to the S254A mutant protein (see Additional file 9).

## Discussion

### Histidine kinases obtained from phosphoproteome analysis

#### Phosphorylation sites

Three histidine kinases, i.e., SPLC1_S041070, SPLC1_S540750 and SPLC1_S081620, were found (see Additional file 3). SPLC1_S041070, or Hik28, can be classified by COG-category into the regulatory function group, and the kinase contains eight p-sites, all of which were detected only under low temperature. pT598, pT793, pT837 and pS805 are located in the membrane spanning domain, whereas pS1482 is in the SsrA domain. The two p-sites, pS911 and pS976, are located in the Bae domain of the protein. The BaeS superfamily domain is involved in the signal transduction mechanism [15], was found on NifC, a negative regulator of nitrogen fixation, and has also been reported to play a role in binding with NifA [16]. Thus, S976 was chosen for further study on its possible role in protein-protein interaction using the Y2H system.

#### Protein-protein interaction networks

According to the PPI subnetwork of Hik28, NIES39_D04330 or SPLC1_S041070, constructed using STRING (see Additional file 6), the association of this low-temperature responsive Hik with other key proteins, i.e., several TCS proteins, response regulator receiver/diguanylate cyclase (PleD), serine/threonine kinase (SPLC1_S240280) and proteins in nitrogen assimilation was revealed. The evidence indicated the possible role of the key TCS protein, Hik28, in the protein binding.

#### Validations of the possible role of Hik28

##### (i) Role in protein binding

The Y2H technique was applied for the binding study of the wild-type (WT)- SPLC1_S041070 and the site-directed-mutant S976A- SPLC1_S041070 with their possible client proteins, TCS proteins, PleD, Ser/Thr kinase and GlsF suggested by the STRING database (Table 2). The positive results were observed between the WT-Hik28 and the client proteins, whereas the negative results were shown in case of the S976A-mutant and the clients. The evidence indicated the role of Hik28 in the protein binding, most likely at the S976 residue. It is also worth noting that the two TCS proteins in the PPI subnetwork, SPLC1_S540750 (Hik21) and SPLC1_S360070, also showed interaction with the response regulator, PleD (see Additional file 9).

##### (ii) role involved fatty acid desaturation

The PPI subnetwork of the orthologous protein of SPLC1_S041070, Sll0474, a signal transduction response regulator protein containing receiver domain showed its connections to a group of proteins in the two-component system including the ones involved in chemotaxis (see Additional file 7). Furthermore, the protein relates to the glycerolipid metabolism, including delta-9 desaturase encoded by *desC* gene, via the proteins in the two-component system, Sll0041 and Sll0043. Therefore, the ΔSll0474 mutant was constructed in *Synechocystis* sp. PCC6803 to examine its role involved the fatty acid desaturation. In cyanobacteria, the fatty acids, C18:0 and C16:0, are at the *sn-1* and *sn-2* positions of glycerolipids. The data in Table 3 clearly showed the accumulation of 16:1^Δ9^ in the mutant strain.

The cyanobacteria, *A. platensis* and *Synechocystis* sp. PCC6803 are classified into group 3 and 4, respectively [17]. *A. platensis* have only one *desC* gene, unlike *Synechocystis* sp. PCC6803, which *desC1* and *desC2* that specific to Δ9-desaturation at the *sn-1* and *sn-2* positions are present [18]. It should be noted that *A. platensis*-*desC* contains the same conserved domains as the *Synechocystis* sp. PCC6803-*desC1* [17]. Thus, the accumulation of 16:1^Δ9^ at the *sn-2* position in the mutant strain suggests that Sll0474 or Hik28 plays a role in the activity of *desC1* that specific to the Δ9-desaturation of C18:0 at the *sn-1* position. The data leads to the possibility that in *A. platensis*, the specificity of the *desC* gene is controlled by signaling proteins instead of having two isoforms of the gene. Thus, the result strongly supports the protein networking in Additional file 7.

##### (iii) role involved N-metabolism

The association of Sll0474 and N-metabolism via nitrogen regulatory protein PII (GlnB), which is well known as signal perceiving protein for the C/N balance, was also shown in the PPI subnetwork (see Additional file 7) [19]. To elucidate the possible role of Hik28 involved in N-metabolism, the photosynthetic activity of the WT and the Δsll0474 cells grown under nitrogen depletion at 35 °C and 16 °C were measured. The significant reduction of the photosynthetic activity after N-depletion in both WT and MT was detected under the optimal temperature. Moreover, the increasing of the reduction level observed in the MT strain and vice versa in the WT strain after the temperature downshift suggests the possible roles of Hik28 in low temperature response and N-assimilation (Table 4).

### Other phosphoproteins

In addition to the group of phosphorylation histidine kinases, a group of four phycocyanin proteins, ApcC, ApcE, ApcF, and CpcB, were identified in their phosphorylated forms. Phosphorylation of phycocyanin has been found earlier by several research groups [20, 21]. Recently, Chen et al. reported the p-sites S22, S49, T94 and S154 on CpcB of *Synechocystis sp.* PCC 6803 and proposed that the p-sites were involved in the energy transfer and state transition of photosynthesis in the model cyanobacteria [22]. According to our phosphoproteome data, CpcB of *A. platensis* C1 was phosphorylated at S46 and T50 after exposure to 40 °C. Interestingly, S46 and T50 are not conserved between *Arthrospira* and *Synechocystis*; however, they are conserved between *Phormidium* and *Lyngbya* spp., which are marine cyanobacteria. Moreover, the S46 and S49 are located exactly at the dodecamer interface (polypeptide binding sites) of CpcB. Therefore, it is possible that pS46 plays a role in the formation of dodecamers under high-temperature stress.

### Bi-level regulated proteins: their protein-protein interaction (PPI) networking and possible role in PPI under stress temperatures

The bi-level regulated proteins (Box 7, Fig. 1) were identified due to the hypothesis that these proteins and their PPI network should play crucial roles in responses to temperature stresses because of their tight regulation. Thus, the networking of this group of proteins was used as the point of origin to follow and, subsequently, reach the affected points in biological pathways.

Accordingly, an important enzyme in nitrogen metabolism, SPLC1_S270380 or ferredoxin-dependent glutamate synthase (GlsF, GltB or Fd-GOGAT), was found in this group. GlsF is an enzyme at the inter-connection of carbon and nitrogen metabolism. It is a complex iron-sulfur flavoprotein that catalyzes the reductive synthesis of L-glutamate from 2-oxoglutarate and L-glutamine via intramolecular channeling of ammonia [23]. The enzyme was drastically upregulated upon a low-temperature shift. Its phosphorylated form at T234 was detected at 35 °C and 22 °C, whereas the form at Y237 was found at the three experimental growth temperatures. The phosphorylated residue pY237 of GlsF is located in the glutamine amidotransferase class-II (GATase) domain (Table 5). The GATase domain catalyzes the amide nitrogen transfer from glutamine to the appropriate substrate. In this process, glutamine is hydrolyzed to glutamic acid and ammonia [24]; therefore, this domain is important for the catalytic activity of the enzyme. Thus, it is likely that phosphorylation of Y237 under all growth temperatures is required for the catalytic activity. In contrast, dephosphorylation of T234 occurred at 40 °C and was possibly related to the high-temperature response mechanism.

**Table 5:** List of bi-level regulated proteins and the experimental conditions under which the proteins were identified

No.	Locus_tag	Locus_tag of ortholog in *A. platensis* group	Phosphoprotein name	Peptide sequence	P-sites	#Phosphopeptides	#Unique phosphopeptides	#P-sites				Temp.			Regulation ([22oC] vs [35oC])	Regulation ([40oC] vs [35oC])
								pS	pT	pY	Total	22°C	35°C	40°C		
**1**	SPLC1_S204380	NIES39_A07260	16S rRNA (uracil(1498)-N(3))-methyltransferase	TGQGKGyVYNSLLLAIGPEGGWtTPEVEEAINRR	Y192y T208t	5	2		1	1	5	✓			up	up
	SPLC1_S204380	NIES39_A07260	16S rRNA (uracil(1498)-N(3))-methyltransferase	TGQGKGyVYNSLLLAIGPEGGWTtPEVEEAINRR	Y192y T209t				1				✓			
	SPLC1_S204380	NIES39_A07260	16S rRNA (uracil(1498)-N(3))-methyltransferase	tGQGKGyVYNSLLLAIGPEGGWTTPEVEEAINRR	T186t Y192y				1					✓		
	SPLC1_S204380	NIES39_A07260	16S rRNA (uracil(1498)-N(3))-methyltransferase	TGQGKGYVYNSLLLAIGPEGGWttPEVEEAINRR	T208t T209t							✓	✓	✓		
	SPLC1_S204380	NIES39_A07260	16S rRNA (uracil(1498)-N(3))-methyltransferase	GYVYNSLLLAIGPEGGWTTPEVEEAINRRFQPVSLGsR	S227s			1						✓		
**2**	SPLC1_S101160	NIES39_Q01000	adenylate cyclase	SITNTVLEtGNAILTSDAHVDER	T215t	2	1		1		2	✓	✓		down	down
	SPLC1_S101160	NIES39_Q01000	adenylate cyclase	SITNTVLETGNAILTsDAHVDER	S222s			1						✓		
**3**	SPLC1_S208580	NIES39_K04210	adenylate/guanylate cyclase domain-containing protein	LWQSQNLPVIEMRVGIFtGPIVAGSLGsR	T556t S566s	1	1	1	1		2			✓	down	up
**4**	SPLC1_S082160	NIES39_E03060	aspartyl/glutamyl-tRNA amidotransferase subunit B	VLEYAVKAARALNCEIAPYsK	S82s	9	8	1			11	✓			up	up
	SPLC1_S082160	NIES39_E03060	aspartyl/glutamyl-tRNA amidotransferase subunit B	IGItRLHMEEDAGKLVHGGsDR	T132t S148s			1	1			✓				
	SPLC1_S082160	NIES39_E03060	aspartyl/glutamyl-tRNA amidotransferase subunit B	LVHGGsDRLAGSTYsMVDFNR	S148s S157s			1					✓			
	SPLC1_S082160	NIES39_E03060	aspartyl/glutamyl-tRNA amidotransferase subunit B	LVHGGSDRLAGstYSMVDFNR	S154s T155t			1	1			✓				
	SPLC1_S082160	NIES39_E03060	aspartyl/glutamyl-tRNA amidotransferase subunit B	LHMEEDAGKLVHGGSDRLAGStySMVDFNR	T155t Y156y					1				✓		
	SPLC1_S082160	NIES39_E03060	aspartyl/glutamyl-tRNA amidotransferase subunit B	RIVRYLGVsDGNMQEGSLR	S200s			1				✓	✓	✓		
	SPLC1_S082160	NIES39_E03060	aspartyl/glutamyl-tRNA amidotransferase subunit B	YLGVsDGNMQEGSLR	S200s							✓				
	SPLC1_S082160	NIES39_E03060	aspartyl/glutamyl-tRNA amidotransferase subunit B	CDVNISVRPVGQKEFGtK	T227t				1			✓				
	SPLC1_S082160	NIES39_E03060	aspartyl/glutamyl-tRNA amidotransferase subunit B	VLTDDRtVAQYFEATVAAGADtK	T342t T357t				2				✓			
**5**	SPLC1_S102760	NIES39_Q02320	beta-ketoacyl-[acyl-carrier-protein] synthase II	ALStRNDDPLHACRPFDVGR	T214t	1	1		1		1	✓			up	down
**6**	SPLC1_S208940	NIES39_K04600	cell division protein FtsH	LAEEIVFGEEEVTTGAsNDLQQVTRVAR	S486s	2	1	1			2	✓			up	down
	SPLC1_S208940	NIES39_K04600	cell division protein FtsH	LAEEIVFGEEEVTTGASNDLQQVtRVAR	T493t				1			✓				
**7**	SPLC1_S034010	NIES39_D02960	crossover junction endodeoxyribonuclease RuvA	LFGNHRTNsDIDNLAGACLDALTLQGAGVLMDDR	S231s	2	1	1			2		✓		down	down
	SPLC1_S034010	NIES39_D02960	crossover junction endodeoxyribonuclease RuvA	LFGNHRTNSDIDNLAGACLDALtLQGAGVLMDDR	T245t				1				✓			
**8**	SPLC1_S205200	NIES39_A07940	cytochrome c biogenesis protein	MSETLQTQLYEIAQFANtLVR	T18t	1	1		1		1	✓	✓		up	down
**9**	SPLC1_S540320	NIES39_M01840	diguanylate cyclase	LRHSAsHDSLTDLWNR	S755s	3	2	1			4		✓		down	down
	SPLC1_S540320	NIES39_M01840	diguanylate cyclase	LtDSVDsHILARLGGDEFTILLENIRDIQEAIDVAER	T821t S826s			1	1					✓		
	SPLC1_S540320	NIES39_M01840	diguanylate cyclase	LTDsVDsHILARLGGDEFTILLENIRDIQEAIDVAER	S823s S826s			1						✓		
**10**	SPLC1_S270810	NIES39_J05180	DNA-binding protein	AILDLAFSAISQEIEsGQPVVVRGLGK	S38s	1	1	1			1		✓		up	up
**11**	SPLC1_S430280	NIES39_O01770	D-tyrosyl-tRNA(Tyr) deacylase	sVQDIGGELLVVSQFTLYGDCR	S68s	3	1	1			3		✓	✓	down	up
	SPLC1_S430280	NIES39_O01770	D-tyrosyl-tRNA(Tyr) deacylase	SVQDIGGELLVVsQFTLYGDCR	S80s			1				✓				
	SPLC1_S430280	NIES39_O01770	D-tyrosyl-tRNA(Tyr) deacylase	SVQDIGGELLVVSQFTLyGDCR	Y85y					1		✓		✓		
**12**	SPLC1_S270380	NIES39_J05540	glutamate synthase	tIVYKGMVRSAVLGEFYR	T234t	2	1		1		2	✓	✓		up	down
	SPLC1_S270380	NIES39_J05540	glutamate synthase	TIVyKGMVRSAVLGEFYR	Y237y					1		✓	✓	✓		
**13**	SPLC1_S082010	NIES39_E02970	histidine kinase	LVHAPSSSSIFHItKLMSR	T206t	2	1		1		2		✓		down	down
	SPLC1_S082010	NIES39_E02970	histidine kinase	LVHAPSSSSIFHITKLMsR	S210s			1					✓	✓		
**14**	SPLC1_S230960	NIES39_L00910	histidine kinase	IGLVSAPPEVFLKtPsEMDEATQK	T320t S322s	4	2	1	1		6	✓	✓	✓	down	up
	SPLC1_S230960	NIES39_L00910	histidine kinase	IGLVSAPPEVFLKTPsEMDEAtQK	S322s T328t				1				✓			
	SPLC1_S230960	NIES39_L00910	histidine kinase	IVtYLQSsQQELISVNR	T215t S220s			1	1			✓				
	SPLC1_S230960	NIES39_L00910	histidine kinase	IVTYLQssQQELISVNR	S219s S220s			1				✓	✓			
**15**	SPLC1_S040030	NIES39_D03400	hydrogenase accessory protein HypB	LNsAVIVGDLETDNDAQRLR	S129s	1	1	1			1			✓	down	up
**16**	SPLC1_S260960	ARTHRO_390015	hydroxymethylbilane synthase	EDPADALVVHENHRDKQLDtLPPGAVVGTSSLR	T127t	1	1		1		1		✓		down	down
**17**	SPLC1_S051730	NIES39_D06980	hypothetical protein	FNGDGTLVWAQSIGGSDLDSGNGIAVDDAGNVYATGsFSSR	S285s	2	1	1			2	✓			up	down
	SPLC1_S051730	NIES39_D06980	hypothetical protein	FNGDGTLVWAQSIGGSDLDSGNGIAVDDAGNVYATGSFSsR	S288s			1				✓				
**18**	SPLC1_S130380	NIES39_R00890	hypothetical protein	KYSIADMDsTSGDLAVETYEIVAEMITKEVALGGQVASMR	S149s	3	1	1			3	✓		✓	down	down
	SPLC1_S130380	NIES39_R00890	hypothetical protein	KYSIADMDStSGDLAVETYEIVAEMITKEVALGGQVASMR	T150t				1				✓			
	SPLC1_S130380	NIES39_R00890	hypothetical protein	KYSIADMDSTSGDLAVETYEIVAEMItKEVALGGQVASMR	T167t				1			✓	✓	✓		
**19**	SPLC1_S171520	NIES39_A02180	hypothetical protein	FIPAFGSAIAASWAFAYTGALGEATCVyFGDLMGGK	Y398y	2	1			1	2	✓	✓	✓	down	up
	SPLC1_S171520	NIES39_A02180	hypothetical protein	FIPAFGsAIAASWAFAYTGALGEATCVYFGDLMGGK	S377s			1					✓			
**20**	SPLC1_S202870	NIES39_A05660	hypothetical protein	IWTFEQVQGILyVVVPIRMtVIR	Y56y T64t	4	2		1	1	5	✓			up	down
	SPLC1_S202870	NIES39_A05660	hypothetical protein	LDMGGLLVyAPVAPTPECIRLVNELVAEYGEVR	Y76y					1		✓	✓			
	SPLC1_S202870	NIES39_A05660	hypothetical protein	LDMGGLLVYAPVAPtPECIRLVNELVAEYGEVR	T82t				1			✓				
	SPLC1_S202870	NIES39_A05660	hypothetical protein	LDMGGLLVYAPVAPTPECIRLVNELVAEyGEVR	Y96y					1			✓			
**21**	SPLC1_S120070	SPLC1_S530570	hypothetical protein	QLVDLTDAKIVyPITFEERK	Y36y	2	1			1	2	✓	✓	✓	up	down
	SPLC1_S120070	SPLC1_S530570	hypothetical protein	QLVDLtDAKIVYPITFEERK	T30t				1				✓			
**22**	SPLC1_S510820	NIES39_O06510	IMP dehydrogenase	LGVTVDIIADLHPINPFNFVMESYAERyPFIYEEQLGR	Y102y	2	1			1	2	✓			up	down
	SPLC1_S510820	NIES39_O06510	IMP dehydrogenase	LGVTVDIIADLHPINPFNFVMESYAERYPFIyEEQLGR	Y106y					1		✓				
**23**	SPLC1_S205080	NIES39_A07830	isoaspartyl peptidase	GGVEtVRKSLYQVITEVYGLLEK	T27t	1	1		1		1		✓		up	up
**24**	SPLC1_S060060	NIES39_D06730	lysophospholipase	MLRLEHISKIYPtGtILK	T13t T15t	3	2		2		4	✓	✓		up	down
	SPLC1_S060060	NIES39_D06730	lysophospholipase	IIAGEVEPTSGEVIKPSSLHIAYLTQEFEVDPARtVREEFWR	T80t				1			✓		✓		
	SPLC1_S060060	NIES39_D06730	lysophospholipase	IIAGEVEPTSGEVIKPSSLHIAYLtQEFEVDPARTVREEFWR	T70t				1				✓			
**25**	SPLC1_S207550	NIES39_K03280	membrane protein	IVSLSSIAGAITIAALMIItGQPLPYQIFAIAAGtYVIWRHR	T180t T195t	2	1		2		3	✓		✓	up	down
	SPLC1_S207550	NIES39_K03280	membrane protein	IVSLSSIAGAITIAALMIItGQPLPyQIFAIAAGTYVIWRHR	T180t Y186y					1		✓		✓		
**26**	SPLC1_S410530		MULTISPECIES: HNH endonuclease	QVIAAQLPDGsALVEFVR	S61s	1	1	1			1				down	up
**27**	SPLC1_S542340		peptidase	VVLVtGIAGFIGSAVARLLISKNQK	T13t	3	2		1		4			✓	down	down
	SPLC1_S542340		peptidase	LASENYMKIysHQYNISSVALR	Y163y S164s			1		1				✓		
	SPLC1_S542340		peptidase	LASENYMKIYsHQyNISSVALR	S164s Y167y					1		✓	✓			
**28**	SPLC1_S201490	NIES39_A04370	phosphate permease	LsGRTFFHRWQLAEALAEESESWQFQPPITR	S445s	2	1	1			2			✓	down	up
	SPLC1_S201490	NIES39_A04370	phosphate permease	LSGRTFFHRWQLAEALAEESESWQFQPPItR	T473t				1					✓		
**29**	SPLC1_S240360	NIES39_K02580	phosphoglycerate dehydrogenase	SAVNIPGLYPDALEQLKPYLQLAETLGNLVsQLVGGR	S353s	1	1	1			1			✓	up	up
**30**	SPLC1_S082540	NIES39_C04520	CpcD phycobilisome linker domain protein	VTACVPSQTRIRtQR	T18t	1	1		1		1			✓	down	down
**31**	SPLC1_S203810	NIES39_A06620	type I restriction endonuclease subunit R	LQAVRyKQsFDK	Y606y S609s	1	1	1		1	2	✓	✓	✓	down	down
						**70**	**44**	**30**	**36**	**15**	**81**					

In terms of signaling proteins, two multi-sensor histidine kinases, SPLC1_S082010 and SPLC1_S230960, were bi-level regulated under the temperature stresses, and the p-sites were detected on serine, threonine and tyrosine residues. The signal transduction mechanism is the first response mechanism when cells encounter stresses; thus, the mechanism is also focused on in this study. According to the hypothesis that the proteins tightly regulated at the translational and post-translational levels could possibly play critical roles in the response mechanism, attention was drawn to the two multi-sensor histidine kinases. First, the SPLC1_S082010 (orthologous of NIES39_M02160) was in its phosphorylated form, pS210, at 35 °C and 40 °C. This p-site is located in the CBS domain known to play a role in ligand binding [25]. Moreover, the expression level of the TCS was upregulated in response to low temperatures. In contrast, SPLC1_S230960 (ortholog of NIES39_L00910) was upregulated at 40 °C, and the protein contained several p-sites. However, there were two specific sites found only after the low-temperature shift, pT215 and pS220, which are located in the sensory domain-DICT superfamily. In contrast, pT320 and pS322, located in the DUF domain of the protein, were detected under all experimental temperatures.

In accord with the domain analysis mentioned above, the regulation pattern of the TCS proteins under temperature stress was revealed by identification of the bi-level regulated proteins. The two key TCS proteins presented different levels of regulation in response to the two stress temperatures. Moreover, PPI sub-networking revealed the connections between the two TCS proteins and the nitrogen regulatory protein PII (GlnB) that is directly connected to the ammonium transporter, which is associated with glutamate synthase (see Additional file 12). In addition to the protein in the nitrogen assimilation pathway, the networks of the two TCS proteins (see Additional file 12) also showed a link with proteins in chemotaxis, CheY and CheW. The evidence clearly indicated the key TCS proteins in the temperature stress response mechanism of *S. platensis* C1 and their first target pathway, nitrogen assimilation, at the point involving regulation of the C/N balance in the cells.

Using STRING, the interactions between the PleD-like GGDEF-domain containing protein, GlsF, and the two TCS proteins, including SPLC1_S041070, were revealed (see Additional file 13). PPI analysis using STRING suggested that PleD might act as a connector between TCS and N-assimilation. There are several GGDEF domain-containing proteins in *A. platensis* C1, which can be annotated as PleD-like proteins. SPLC1_S490280 is a PleD-like protein containing a GGDEF domain at the C-terminus, and it showed a high identity with the PleD protein, SPLC1_S531000. The GGDEF domain is widely present in bacteria and is involved with a variety of cell signaling proteins [26, 27]. The sequence alignments of PleD and PleD-like proteins of *A. platensis* C1 and their orthologs are shown in Additional file 14. It should be noted that a PleD-like protein, SPLC1_S490280, was previously detected as an upregulated protein found in common under the two temperature stresses [10]. Thus, the PleD-like protein SPLC1_S490280 was selected to represent PleD-like proteins to validate the PPI.

The protein pairs suggested by STRING were examined for their interactions using the Y2H technique (see Additional file 9). The positive results were observed between PleD and glutamate synthase and TCS proteins, suggesting the possible role of PleD-like proteins in signal transduction between TCS and GlsF in N-metabolism. Furthermore, protein sequences in the same orthologous group as SPLC1_S082010, or TCS, SPLC1_S540750 and SPLC1_S360070 were also identified using CyanoCOG (http://www2.sbi.kmutt.ac.th/orthoCOG/cyanoCOGnew/home) and examined for their PPI subnetworks using STRING (see Additional file 15A-C).

### Evidence of the cross-phosphorylation of Hik and Ser/Thr kinases

Rapid responses to environmental changes are important for cell survival. Proteins in signal transduction mechanisms are the first group of proteins regulated upon sensing external stimuli. TCS is a well-known signal transduction system in prokaryotes, including cyanobacteria, in which His/Asp residues are phosphorylated. Recently, increasing attention has been drawn to Ser/Thr kinases, which undergo autophosphorylation and/or transphosphorylation by another kinase. These kinases are activated via autophosphorylation of the activation loop, which is induced by ligand binding [28]. A previous study showed that the pT on the activation loop of Ser/Thr kinases is preferentially involved in interactions with proteins containing FHA domains, contributing to the physiological roles of this group of kinases [29].

In the *A. platensis* C1 phosphoproteome, the pT of the activation loop was not identified. However, upon temperature stress exposure, SPLC1_S240280 contained p-site in the p-loop, whereas the p-sites were located in the αG- and αH- loops of SPLC1_S032990 and the αF- loop of SPLC1_S280030 and SPLC1_S541370 (see Additional file 5A-B). The p-loop plays an important role in phosphoryl transfer and ATP/ADP exchange during enzyme catalysis. The conformational changes allow the transfer of γ-phosphate from ATP to the phospho-acceptor Ser or Thr residue in the substrate protein [30, 31]. The αF- loop is involved in protein-protein interactions with regulatory proteins, while the αG-loop located on the interface of the front-to-front dimer plays a role in dimerization of this kinase [32, 33]. This dimerization creates an allosterically activated kinase that could phosphorylate and, thereby, activate other kinases [33].

Moreover, previous reports have shown that bacterial Ser/Thr kinases show homology in their catalytic domains with eukaryotic Ser/Thr kinases, eSTKs [33]. The eight phosphorylated Ser/Thr kinases identified in the present study showed homology with the eSTKs, as shown in Additional file 5A-B. The consensus catalytic domain, HRDLKxxN, was first reported as a eukaryotic Ser/Thr kinase [34], and later, its homologs, HRDVKxxN and YRDLKxxN, were revealed in prokaryotic cells [33]. According to *A. platensis* C1 phosphoproteome data, all of the identified phosphorylated Ser/Thr kinases represented HRDIKPxN or YRDIKPxN. Both pS and pT were detected on these kinases, whereas pY was only detected on SPLC1_S580170 after a temperature downshift (see Additional file 16). The p + 1 loop was reported as a critical point for the binding of these kinases and their substrates, leading to distinct substrate specificity between Ser/Thr and tyrosine kinases [28, 35]. However, the p-sites detected on these two kinases were not located in the p + 1 loop.

Notably, cross-phosphorylation of Ser/Thr kinase (donor) and TCS (recipient) was revealed in both Gram-negative and Gram-positive bacteria [33]. Accordingly, in the present study, evidence of PPI between the Ser/Thr kinase and the TCS was also observed using Y2H experiments (Table 2). Thus, the presence of pS and pT on the TCS proteins in the present phosphoproteome data might reflect cross-phosphorylation. Although the roles of pS and pT on TCS proteins have not been studied as extensively as phosphorylated histidine, there is some evidence showing roles in functional regulation and ligand binding [36]. This evidence emphasizes the critical role of phosphorylation in regulation at the interactome level.

In addition to the cross-phosphorylation event, another possible cause of the presence of pS and pT on the multi-sensor histidine kinase proteins is non-canonical TCS. In plants, significant evidences of non-canonical TCS have been reported. Phosphorylated serine residues of Hik, produced by phosphotransfer from histidine residues of Hik, have been found, suggesting that histidine kinases can evolve into kinases that exhibit serine/threonine kinase activity [37, 38]. However, there was a report on the autophosphorylation of the serine residue in Hik-based ethylene signal transduction in plants that did not involve the conserved histidine on the Hik-domain, leading to the conclusion that the signal transduction did not occur by a phosphorylation mechanism [39].

To validate the protein interactions obtained from PPI network, the SPLC1_S240280 Ser/Thr kinase was chosen as the representative of the group for PPI analysis because it contained a p-site at the critical loop for phosphoryl transfer. Y2H analysis showed that the kinase could interact with several TCS proteins, i.e., Hik21, Hik28, and SPLC1_S081620. This suggested that cross-phosphorylation events most likely occurred in *A. platensis* C1 under the experimental conditions (see Additional file 9).

### Integration of bi-level regulated proteins, their PPI network and differentially expressed proteins: revealing low- and high-temperature response mechanisms

**Fig. 4:**
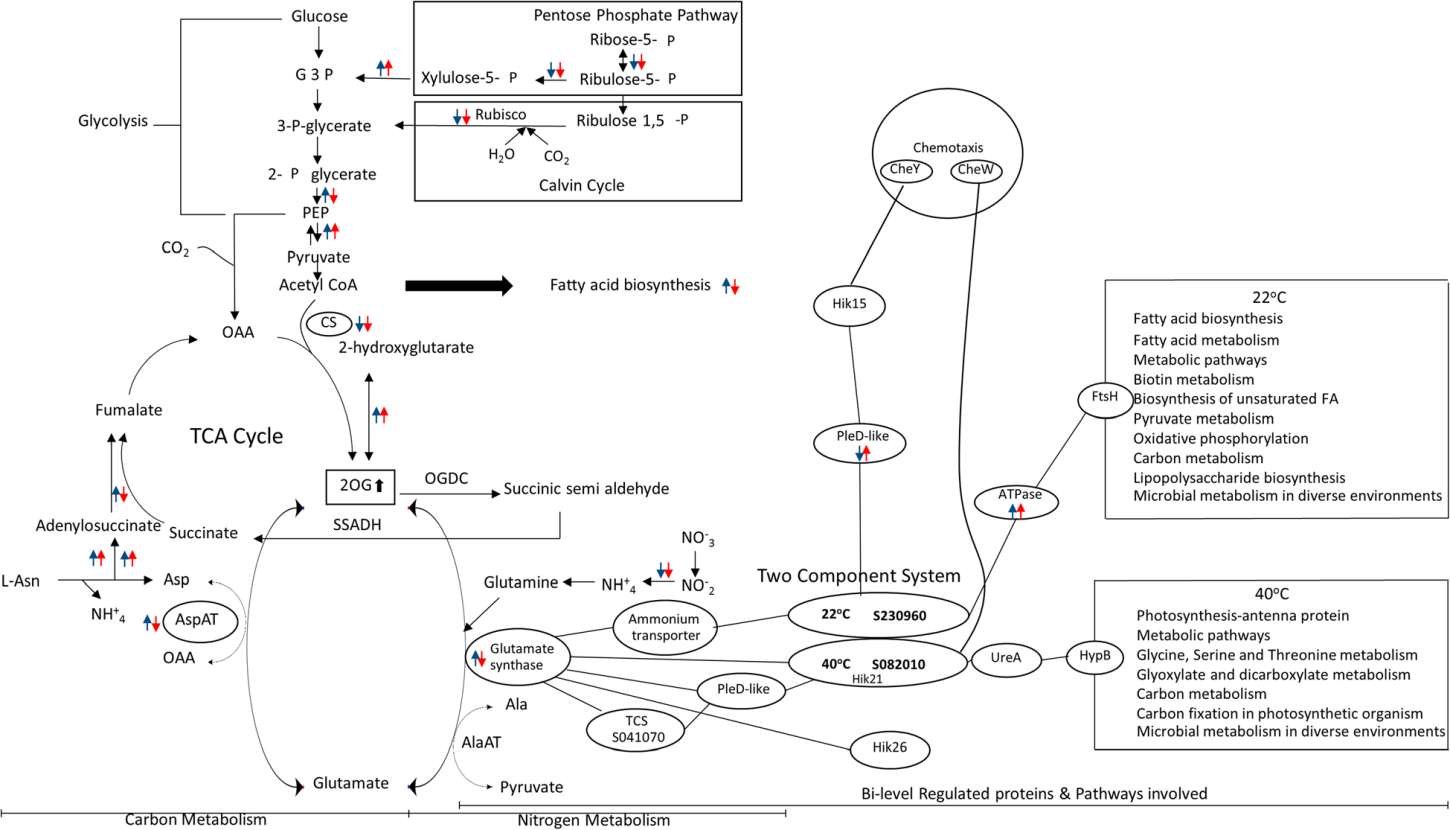
Schematic representation of the potential temperature stress response mechanism of *A. platensis* C1 with the two key TCS proteins at the center of the response process, and the proteins in metabolic pathways that being affected by the stress. The edge represents protein-protein relationships, interactions, co-occurrences, gene-neighbors or text mining. The oval-node represents a protein. The blue and red arrows indicate the up- or down-regulation of the proteins identified at 22 °C and 40 °C, respectively. The “Sxxxxxx” in the oval node represents the accession number of *A. platensis* C1, SPLC1_Sxxxxxx. Abbreviation; G-3-P: glyceraldehyde-3-phosphate, 2OG: 2-oxoglutarate, OAA: oxaloacetate, CS: citrate synthase, SSADH: succinic semi-aldehyde dehydrogenase, OGDC: Oxoglutarate decarboxylase, AspAT: aspartate aminotransferase, AlaAT: alanine aminotransferase

When the data on the bi-level regulated proteins and their PPI networks were integrated with the differentially expressed proteins, the effect of temperature stresses on enzymes and proteins in the pathways of *A. platensis* C1 could be simplified as shown in Fig. 4. First, the two component system proteins in the signal transduction pathway were examined due to their direct role in sensing and transducing signals. As mentioned earlier, SPLC1_S230960 and SPLC1_S082010 were bi-level regulated in response to low- and high-temperature stress, respectively. Both TCS proteins associated with glutamate synthase were also bi-level regulated. It should be addressed that phosphoglycerate dehydrogenase catalyzing 2-oxoglutarate (2-OG) biosynthesis from 2-hydroxyglutarte was upregulated approximately 1.5-fold at the protein-expression level under both stresses. This suggests an increasing level of 2-OG, which is known as the metabolite that senses the C/N balance of cyanobacteria [23]. Moreover, several enzymes at the interconnection of C- and N-metabolism were also differentially expressed upon temperature stresses (Fig. 4). Thus, this evidence supports that the C/N ratio is tightly regulated in the temperature-stress response mechanism.

In addition to controlling the C/N balance, pathways affected by bi-level regulated proteins were determined by following the interaction paths in the PPI network of the bi-level regulated proteins detected under low- and high-temperature stress (Fig. 4). Fatty acids and unsaturated fatty acids biosynthesis, lipopolysaccharide biosynthesis, pyruvate metabolism and oxidative phosphorylation were affected only by the low temperature, whereas the high temperature response of the cells acted on photosynthesis, carbon fixation, glycine/serine/threonine metabolism and glyoxylate/dicarboxylate metabolism.

Regarding energy metabolism under the temperature stresses, the enzyme ATP synthase was upregulated under both stress temperatures, whereas the enzyme adenylate kinase, which catalyzes the reversible reaction of 2ADP production from ATP and AMP, was upregulated only upon temperature upshift. Its function is to maintain a constant concentration and fixed ratio of adenine nucleotides and to monitor the cellular energy state through nucleotide sensing and signaling [40]. The enzyme was reported to keep a high ATP level in mitochondria by recycling ADP as the phosphate acceptor in oxidative phosphorylation, and a similar process was found in cyanobacteria [40, 41]. This evidence from the present comparative proteome analysis shows the possibility of the energy production via ATP synthase under both stresses and via combined action of ATPase and adenylate kinase only under high-temperature stress. The finding was supported by the report of Burkart et al., which indicated that the induction of the unfolded protein response, which is known to occur upon high-temperature exposure [42], is linked to mitochondrial energy metabolism via adenylate kinase [41].

## Conclusions

The present study shows the integration of multi-level analyses to explore the temperature stress response mechanisms of *A. platensis* C1. The identification of bi-level regulated proteins obtained from quantitative proteome and phosphoproteome analyses could reveal the key TCS proteins for low- and high-temperature stresses, together with evidence that supported the cross-phosphorylation of the two-component system and Ser/Thr kinases. Moreover, the comparative pattern profiling of phosphoproteins could imply switching between functional- and non-functional forms of the proteins via their phosphorylation patterns. The role of the p-site in the protein-protein interactions in the present work supported the previous reports on a critical role in protein-protein interaction of the p-sites, especially in the signal transduction mechanism [43, 44]. Taking the quantitative and phosphoproteome data together leads to the conclusion that in response to growth-temperature stress, the signaling mechanism can be linked to the metabolite that controls the C/N balance and the key enzyme in N-assimilation of the cells.

## Methods

### Culture conditions and temperature stresses

Axenic cultures of *A. platensis* strain C1, obtained from Prof. Avigad Vonshak, Algal Biotechnology, Ben-Gurion University of the Negev, Israel, were grown at the optimal growth temperature, 35 °C, under illumination by 100 μEm^− 2^ s^− 1^ fluorescent light with continuous stirring in 1.5 L of Zarrouk’s medium [10]. For exposure to temperature stresses, the culture was grown at the optimal temperature until the optical density at 560 nm reached 0.4 (mid-log phase), at which time a cell sample was harvested by filtration before shifting the growth temperature (t = 0 min). The growth temperature was immediately shifted from 35 °C to 22 °C for the low-temperature stress and from 35 °C to 40 °C for the high-temperature stress for 180 min, followed by cell harvesting using paper filtration (see Additional file 17). Three biological replicates were prepared for each condition. The time-point at 180 min after the stress exposure was chosen according to the data obtained in the previous studies [8–10], which showed the saturated expression of the majority of the stress-response proteins.

In case of *Synechocystis sp.* PCC 6803, wild-type (WT) and mutant (MT), the cultures were grown in BG-11 medium under the light intensity of 70 μEm^− 2^ s^− 1^ at 30 °C until the optical density at 750 nm reached 0.8–0.9 (mid-log phase). Then, the wild type and the deletion mutant cultures were immediately shifted from 30 °C to 16 °C and 45 °C for 60 min for the low- and high-temperature stress, respectively, before subjected to fatty acid analysis. In case of response to nitrogen depletion, after the WT and MT cells were grown until the mid-log phase was reached; the cells were then transferred to the nitrogen depletion BG-11 media and cultivated for 48 h at 30 °C and 16 °C. The chlorophyll content and O_2_-evolution of the cultures were determined [45, 46].

It should be noted that the critical temperature for low- and high temperature stress of *A. platensis* C1 and *Synechocystis sp.* PCC 6803 are different.

### Protein sample preparation and protein digestion

The freeze-dried harvested cells, approximately 120 mg, were dissolved in freshly prepared lysis buffer containing one tablet of complete protease inhibitor (Roche, USA), one tablet of phosSTOP phosphatase inhibitor cocktail (Roche, USA), 20 mM ammonium bicarbonate, pH 8.5, 6 M urea and 2 M thiourea. Subsequently, the cells were lysed by sonication on ice, and the supernatants were obtained by centrifugation at 10,300*×g*, under 4 °C for 30 min. The supernatants were subsequently purified and concentrated by ethanol precipitation (9 volumes of absolute ethanol were added to 1 volume of the supernatant) overnight at − 20 °C, followed by centrifugation. The pellets were washed with ice-cold absolute ethanol and subsequently dissolved in 20 mM ammonium bicarbonate, pH 8.5. The protein concentration was determined using the 2D-Quant kit (GE Healthcare Life Sciences, USA). Subsequently, 10 mM dithiothreitol (DTT) was added to the remaining supernatant and incubated at 60 °C for 10 min. The soluble proteins were purified and digested with trypsin using the FASP protein digestion kit (Expedeon Asia, Singapore). The iodoacetamide (IAA) solution contained in this kit was added to the sample solution, and the mixture was incubated for 20 min at room temperature in the dark. Subsequently, the trypsin stock solution (1 mg/ml) was mixed with the samples at a ratio of 1:75 (w/w). The mixture was incubated at 37 °C for 16 h. Subsequently, the peptide mixture was acidified to pH ≤ 3 using trifluoroacetic acid (TFA) and passed through a C_18_ column for desalting.

### Phosphopeptide enrichment using TiO_2_ and desalting

TiO_2_ beads were used to selectively capture and enrich the phosphopeptides. The phosphopeptides from *Spirulina platensis* C1 were only enriched in the first level using eight 10-μl TiO_2_-C_8_ tips (Titansphere Phos-TiO_2_ Tip, GL Sciences, Japan) for one-pot enrichment. Prior to phosphopeptide enrichment, the samples were prepared by adding buffer B containing 0.1% TFA and 50% acetonitrile and buffer C containing 300 mg/ml of lactic acid at a 1:1 ratio. Briefly, the TiO_2_-C_8_ tips were washed with 1% ammonia, preconditioned with buffer B and equilibrated with buffer C. Subsequently, the peptide samples were loaded into the TiO_2_-C_8_ tips and washed five times with buffer B and two times with buffer D (0.1% TFA in ultrapure water), respectively. The phosphopeptides were eluted with elution buffer containing 1% ammonia, pH 11.3. The eluents were acidified to pH ≤ 3 with trifluoroacetic acid (TFA) prior to desalting using C_18_ tips. The C_18_ tips were preconditioned with buffer B and equilibrated with buffer A containing 0.1% TFA and 5% acetonitrile. Subsequently, the samples were loaded into the C_18_ tips and washed with buffer A. The desalted phosphopeptides were eluted with buffer B. The resulting phosphopeptide eluents from the one-pot procedure were desalted using a 10-μl ZipTip (Millipore, USA) and subsequently dried almost to completeness using a speed-vacuum prior to liquid chromatography and tandem mass spectrometry (LC-MS/MS) analysis. The overall workflow is illustrated in Additional file 17.

It should be noted that at least three biological and technical replicates were carried out for each sample, which obtained from each experimental condition, subjected to proteome and phosphoproteome analyses. Moreover, at least two injections were performed on LC-MS/MS for each replicate.

### Liquid chromatography and tandem mass spectrometry (LC-MS/MS)

*Hardware:* All quantitative proteome and phosphoproteome experiments were performed on an Agilent 1260 infinity HPLC-chip/MS interfaced to the Agilent 6545 Q-TOF LC/MS system (Agilent Technologies, USA). In case of column, a Phosphochip II, a microfluidic HPLC-chip/MS technology (Part number G4240–62021, Agilent Technologies, Germany), comprising a 40 nLTiO_2_ enrichment column sandwiched between two high pH stable 100 mL reverse phase enrichment columns, was used in this HPLC-chip/MS system. The tryptic peptides were analyzed on a 75 μm × 150 mm separation column packed with C18-AQ (5 μm). It should be noted that the PhosphochipII can be used for both quantitative proteome and phosphoproteome in the same run.

*Mobile phase and LC condition:* The following mobile phases were employed in the studies: for the capillary pump, (A1/B1) 2% acetonitrile, 0.6% acetic acid and 2% formic acid in water; and for the nanopump, (A2) 0.6% acetic acid and 0.5% formic acid in water and (B2) 0.6% acetic acid and 0.5% formic acid in acetonitrile. The dried peptide samples were solubilized in 3% acetonitrile and 0.3% formic acid in water. The samples were loaded onto the enrichment column at a 4-μL/min-flow rate using 50% A1 and B1. For the non-phosphorylated peptide analysis, the 35-min gradient used for the analytical column was initiated at 40% B2, increased to 70% B2 at 33 min, maintained until 34 min and subsequently reduced to 3% B2 at 35 min. For the phosphorylated peptide analysis, the 36-min gradient used for the analytical column was initiated at 30% B2, increased to 60% B2 at 30 min and 70% B2 at 32 min, maintained until 35 min and subsequently reduced to 3% B2 at 36 min. The column was equilibrated using the polarity setting at the negative mode for 5 min prior to subsequent runs to help the column clean-up.

*MS/MS condition:* Q-TOF MS/MS operating in high resolution, 4 GHz, and positive ion mode was used for all the experiments. The following MS source conditions were used: source temperature 150 °C, capillary voltage 1950 V, fragmentor voltage 140 V and drying gas flow rate 6 L/min. Auto data acquisition mode was performed at a mass range of 100–1400 m/z for MS mode and 80–2000 m/z for MS/MS mode. The acquisition rate for MS and auto-MS/MS mode was 3 spectra/sec.

*Software:* Agilent MassHunter software version B.06.01 was used to control the LC-MS/MS system and to perform data acquisition. Then, the data was analyzed further by Spectrum Mill (version Rev. B.05.00.181 SP1) and Mass Profiler Professional, or MPP, (version B.14.00), which described below.

### Quantitative proteome and phosphoproteome data analyses

The data obtained from LC-MS/MS was analyzed as illustrated in Fig. 1. Data analyses were performed using Agilent Technologies software. LC/MS and LC-MS/MS data were acquired, extracted and evaluated using MassHunter (version B.06.01). Spectrum Mill (version Rev. B.05.00.181 SP1) was used for protein identification, peptide/phosphopeptide distribution and phosphorylated site (p-site) identification. Mass Profiler Professional, or MPP, (version B.14.00) was employed for analysis of protein expression levels and statistical evaluation of technical reproducibility.

The following data extraction criteria were used: modification, carbamidomethylation (C); precursor MH+, 600–6000 Da; and scan time range, 0–300 min. In the MS/MS search, the following criteria were high-stringency criteria: trypsin digestion, allowing up to 2 missed cleavages; fixed modification, carbamidomethylation (C); variable modifications, phosphorylated Ser (S), phosphorylated Thr (T) and phosphorylated Tyr (Y); precursor mass tolerance ±20 ppm; and product mass tolerance ±50 ppm. For the low-stringency criteria, a precursor mass tolerance ±50 ppm and product mass tolerance ±100 ppm were used. However, the high-stringency criteria were always used in the study unless otherwise indicated. The reverse database scores for % false discovery rate (% FDR) were calculated in the search mode. Samples from three different conditions (35 °C, 22 °C and 40 °C) were analyzed and searched individually with each result set. Peptide and protein identification were performed using the most updated *Arthrospira platensis* C1 database, October 2016, (http://www.sbi.kmutt.ac.th/arthrobase/).

Before statistical analysis of the proteome data, the data were filtered by signal threshold. Signals lower than 3 times the noise signals were removed. To identify the proteins and peptides significantly detected by LC-MS/MS, the data obtained was analyzed using the MPP version B.14.00 program. Quality control of all samples was performed by principal components analysis, or PCA, in the quality control mode of the MPP program. Then, significance analysis was performed using appropriate statistics methods, i.e., t-test against zero, unpaired t-test and one-way ANOVA for one group of experimental conditions (all samples under a designated temperature), two groups each with their replicate (comparing between the optimal and a stress temperature) and three groups each with their replicates (comparing among the three temperature conditions), respectively. The cut-off criterion was a *p*-value less than or equal to 0.05. The significantly expressed proteins detected by LC-MS/MS were then analyzed for their P-sites. For analysis of the protein expression level, proteins that passed the significance cut-off criterion were analyzed for fold-change level, and proteins with a differential expression level equal to or more than 1.5 were considered.

### Potential protein-protein interaction (PPI) network construction

Phosphoprotein and bi-level regulated protein networks presenting protein-protein interactions in response to temperature stresses in *A. platensis* C1 were constructed based on the prototype PPI data of *Synechocystis* PCC6803*.* Prototype construction was based on a graph in which the nodes and edges represented homologous proteins and their interactions, respectively. All homologous proteins were identified as COGs (cluster of orthologous groups of proteins) by reciprocal BLAST searches between two species below a threshold e-value of less than 1 × 10^− 10^. The COGs analysis was performed using the OrthoMCL software tool (v1.4) [47], and their group members were subsequently confirmed by similar structures of protein domains when compared against the Pfam database (v24) [48]. An edge was drawn from the bait protein, denoted by a diamond arrow, and targeted to its prey protein at the tail. The network graph demonstrates all *Synechocystis*-referable interactions between selected nodes containing homologous proteins. The phosphorylated proteins identified in the present study and differentially expressed proteins reported in previous studies [8–10] were mapped to their corresponding nodes.

To obtain the most updated details on the PPI network, STRING (http://string-db.org, last accessed: Oct, 2018) [13], a useful web-based tool with the most updated databases, was used. Thus, in the case of PPI-subnetwork construction, the *A. platensis* strain C1 proteins were inferred using their orthologous genes present in *A. platensis* strain NIES39 or *A. maxima*, for which the data are stored in the STRING database. The interactions/associations of interest in the subnetworks were further analyzed for protein-protein binding using Y2H.

### Site-directed mutagenesis and protein-protein interaction analysis using a yeast two-hybrid system (Y2H)

To validate the key interactions between the bi-level regulated proteins and their client proteins suggested by STRING and to determine the role of phosphorylation in protein binding, Y2H assays of these protein pairs were performed. Y2H is a technique widely used for protein-protein and/or domain-domain interaction assays [39, 49]. The role of p-sites in protein-protein interactions was elucidated by site-directed mutagenesis of the designated amino acid residues, and the binding was subsequently verified using Y2H.

*Cloning of TCS (SPLC1_S041070, _S540750, and _S360070), Ser/Thr Kinase (SPLC1_S240280), glutamate synthase (SPLC1_S270380) and PleD-Like (SPLC1_S490280) and site-directed mutagenesis of S976 of SPLC1_S041070:* A Phusion Site-Directed Mutagenesis Kit (Thermo Scientific, USA) was used for constructing target plasmid DNA with desired point mutations. The genomic DNA of *Spirulina* C1 was used as template DNA for PCR amplification of the designated genes and the mutants by using oligonucleotide primers containing single-point mutation, as shown in Additional file 18. After PCR amplification, the PCR products were cloned into pGBKT7 and pGAT7 (see Additional file 19), which are the vectors for cloning and transformation of bait- and prey proteins into *Saccharomyces cerevisiae.*

*Yeast Two-Hybrid System (Y2H):* The bait-containing plasmid system, Y2HGold, and the prey-containing plasmid system, Y187 (Clontech, USA), were used for the designated plasmid DNA transformation. Bait autoactivation was used to detect false positive results prior to the experiments**.** The bait- and prey-containing plasmids were mated in 300 μL of 2xYPDA broth. The positive control (plasmid pGBKT7-p53 in Y2HGold mated with plasmid pGADT7-T in Y187) and negative control (plasmid pGBKT7-Lam in Y2HGold mated with plasmid pGADT7-T in Y187) were also mated under identical conditions. The yeast mating cultures were incubated at 30 °C with shaking at 200 rpm for 24 h. The cultures were subsequently spread onto SD/−Leu/−Trp/X-α-gal/AbA dropout (DDO/X/A) and SD/−Leu/−Trp (DDO) plates and incubated at 30 °C for 3 days. The blue colonies were picked, streaked onto high-stringency SD/−Ade/−His/−Leu/−Trp/X-α-gal/AbA dropout (QDO/X/A) plates, and incubated at 30 °C for 3–5 days. Finally, to confirm the specific interactions between bait and prey proteins, the yeast strains were switched for the bait-containing plasmid and the prey-containing plasmid. The DNA probes used in this work for bait and prey gene cloning are listed in Additional file 18.

### Construction of the *sll0474* deletion mutant

The deletion mutant of Sll0474 (Δsll0474) was constructed in *Synechocystis sp.* PCC6803 to examine the effect of the designated protein, which is an orthologous of SPLC1_S041070. The recombinant plasmid was designed using pGEM-T Easy vector consisting of upstream and downstream regions of *sll0474* gene and a spectinomycin resistance gene inserted between the upstream and the downstream regions. PCR was used to amplify the upstream and the downstream sequences of *sll0474* gene and the spectinomycin resistance gene by using oligonucleotide primers as shown in Additional file 18. The deletion mutation occurred via homologous recombination.

### Fatty acid analyses of *Synechocystis sp.* PCC 6803 wild type and deletion mutant

*Synechocystis sp.* PCC 6803, WT and MT cells, were grown under the three experimental conditions as mentioned above. After the temperature shifts, the cells were then harvested by centrifugation at 8300*×g* for 10 min and washed by using 5 mM HEPES pH 8.0 before freeze dried. The freeze-dry cells were then subjected to transmethylation by using Heptadecanoic acid: C16H33COOH (Sigma, USA) as the internal standard. The transmethylated fatty acids were analyzed by using gas chromatography (GC) [50].

## Supplementary information

**Additional file 1.** List of up- and down- regulated proteins detected after 180 min after the temperature shift by using non-gel proteome analysis.

**Additional file 2. **PPI subnetworks of four groups of the differentially expressed proteins; **(A)** proteins upregulated at 22 °C and downregulated at 40 °C, **(B)** proteins downregulated at 22 °C and upregulated at 40 °C, **(C)** proteins upregulated at both temperatures **(D)** proteins downregulated at both temperatures. The subnetworks were constructed by using STRING. The *A. platensis* C1 proteins were inferred to that of the *A. platensis* NIES39 via orthologous group.

**Additional file 3.** List of 101 proteins after the threshold filtering.

**Additional file 4.** MS/MS spectra of some peptides of interest.

**Additional file 5. **Protein sequence alignment of the eukaryotic type Ser/Thr kinase, PkA, and the Ser/Thr kinases of *A. platensis* C1; **(A)** SPLC1_S240280, SPLC1_S032990 and SPLC1_S280030, and **(B)** SPLC1_S200190, SPLC1_S208550, SPLC1_S532860, SPLC1_S580170 and SPLC1_S541370. The conserved protein domains are labeled.

**Additional file 6. **PPI subnetwork of Hik28. The subnetwork was constructed by using STRING. The *A. platensis* C1 proteins were inferred to that of the *A. platensis* NIES39 via orthologous group.

**Additional file 7. **PPI subnetwork of Hik28 and enzymes involved in fatty acid desaturation. The subnetwork was constructed by using STRING. The *A. platensis* C1 proteins were inferred to that of the *Synechocystis* sp. PCC6803 via orthologous group.

**Additional file 8. **PPI subnetwork of the bi-level regulated proteins. The subnetwork was constructed by using STRING. The *A. platensis* C1 proteins were inferred to that of the *A. platensis* NIES39 via orthologous group.

**Additional file 9.** Protein-protein interaction analyses of the proteins of interest by using yeast two-hybrid system (Y2H).

**Additional file 10. **Construction of Δsll0474 mutant in *Synechocystis* sp. PCC6803; **(A)** Map of the recombinant plasmid containing the upstream (us) and the downstream (ds) regions of the sll0474 gene, and the spectinomycin resistant gene (Spec) cloned in pGEM T-Easy vector **(B)** Enzymatic digestion of the recombinant plasmids as shown in (A) represented the inserts (The recombinant plasmids in the last two lanes of the agarose gel were digested with EcoRI&HindIII and PstI, respectively.) **(C)** PCR products of *Synechocystis* sp. 6803WT and Δsll0474 mutant amplified by using US-sll0474ERIFW and DS-sll0474pstIRVprimers **(D)** PCR products of *Synechocystis* sp. 6803WT and Δsll0474 mutant amplified by using Sll0474ERIFW and Sll0474BaHIRV primers.

**Additional file 11. **Fatty acid analysis profile obtained by using gas chromatography of *Synechocystis* sp. PCC6803 WT and Δsll0474 mutant.

**Additional file 12. **PPI subnetworks of the two multi-sensor histidine kinases, SPLC1_S082010 (orthologous of NIES39_M02160) and SPLC1_S230960 (ortholog of NIES39_L00910), and the glutamate synthase, GlsF. The subnetworks were constructed by using STRING. The *A. platensis* C1 proteins were inferred to that of the *A. platensis* NIES39 via orthologous group.

**Additional file 13. **PPI subnetwork of the SPLC1_S531000: PleD-like GGDEF-domain containing protein and its client proteins, GlsF, SPLC1_S041070, SPLC1_S082010 and SPLC1_S230960. The subnetwork was constructed by using STRING. The *A. platensis* C1 proteins were inferred to that of the *A. platensis* NIES39 via orthologous group.

**Additional file 14. **Protein sequence alignment of PleD from *A. platensis* C1 and PleD-like proteins in the same orthologous group. It shows the conserved GGDEF domain in this group of proteins.

**Additional file 15. **PPI subnetworks of the proteins in the same orthologous group as **(A)** SPLC1_S082010, **(B)** SPLC1_S540750 and **(C)** SPLC1_S360070. The subnetworks were constructed by using STRING. The *A. platensis* C1 proteins were inferred to that of the *A. platensis* NIES39 via orthologous group.

**Additional file 16.** List of the 527 phosphorylated proteins after filtering by the high stringency criteria.

**Additional file 17. **Experimental design and workflow for the phosphoproteome analysis of *Spirulina* in response to immediate temperature elevation and reduction.

**Additional file 18.** List of primers used for gene cloning, site-directed mutagenesis and construction of Sll0474 deletion mutant.

**Additional file 19.** Pictures of pGBKT7 and pGADT7 AD vectors (Clontech Laboratories, Inc. USA).

## Data Availability

All data generated or analysed during this study are included in this published article and its supplementary information files. Some raw data has been deposited in a data repository and can be accessed at http://download.cyanopro.net/C1_data/.

## Abbreviations

2OG: 2-oxoglutarate
AbA: Aureobasidin A
Ade: Adenine
AlaAT: Alanine aminotransferase
AspAT: Aspartate aminotransferase
C-: Carbon
C16:0: Palmitic acid
16:1^Δ9^: Palmitoleic acid
C18:0: Stearic acid
CS: Citrate synthase
DDO: Double dropout medium
DDO/X/A: Double dropout/X-α-gal/Aureobasidin A medium
G-3-P: Glyceraldehyde-3-phosphate
Hik: Histidine kinase
His: Histidine
LC-MS/MS: Liquid chromatography and tandem mass spectrometry
Leu: Leucine
MT: Mutant
N-: Nitrogen
OAA: Oxaloacetate
OGDC: Oxoglutarate decarboxylase
PPI: Protein-protein interaction
pS/pT/pY: Phosphorylated-Serine/−Threonine/−Tyrosine
P-site: Phosphorylated site
QDO/X/A: Quadruple dropout/X-α-gal/Aureobasidin A medium
Q-TOF: Quadrupole time of flight
SD: Synthetically defined medium
*sn-*: Stereospecifically numbered
SSADH: Succinic semi-aldehyde dehydrogenase
TCS: Two-component system
TiO_2_: Titanium dioxide
Trp: Tryptophan
WT: Wild-type
Y2H: Yeast two-hybrid system
YPDA: Yeast extract/peptone/dextrose/adenine hemisulfate
