# Supplementary material for: Revealing the key point of the temperature stress response of Arthrospira platensis C1 at the interconnection of C- and N- metabolism by proteome analyses and PPI networking
Source: BMC Mol Cell Biol. 2020 Jun 12;21:43. doi: 10.1186/s12860-020-00285-y (PMC7291507; doi:10.1186/s12860-020-00285-y)
Supplement: Supplementary file 2 — Additional file 2. PPI subnetworks of four groups of the differentially expressed proteins; (A) proteins upregulated at 22 °C and downregulated at 40 °C, (B) proteins downregulated at 22 °C and upregulated at 40 °C, (C) proteins upregulated at both temperatures (D) proteins downregulated at both temperatures. The subnetworks were constructed by using STRING. The A. platensis C1 proteins were inferred to that of the A. platensis NIES39 via orthologous group. [file 12860_2020_285_MOESM2_ESM.docx]

**Additional file 2**

**
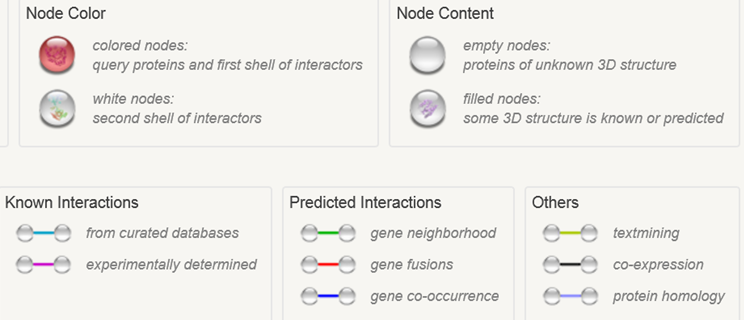
**

**(A)**

**
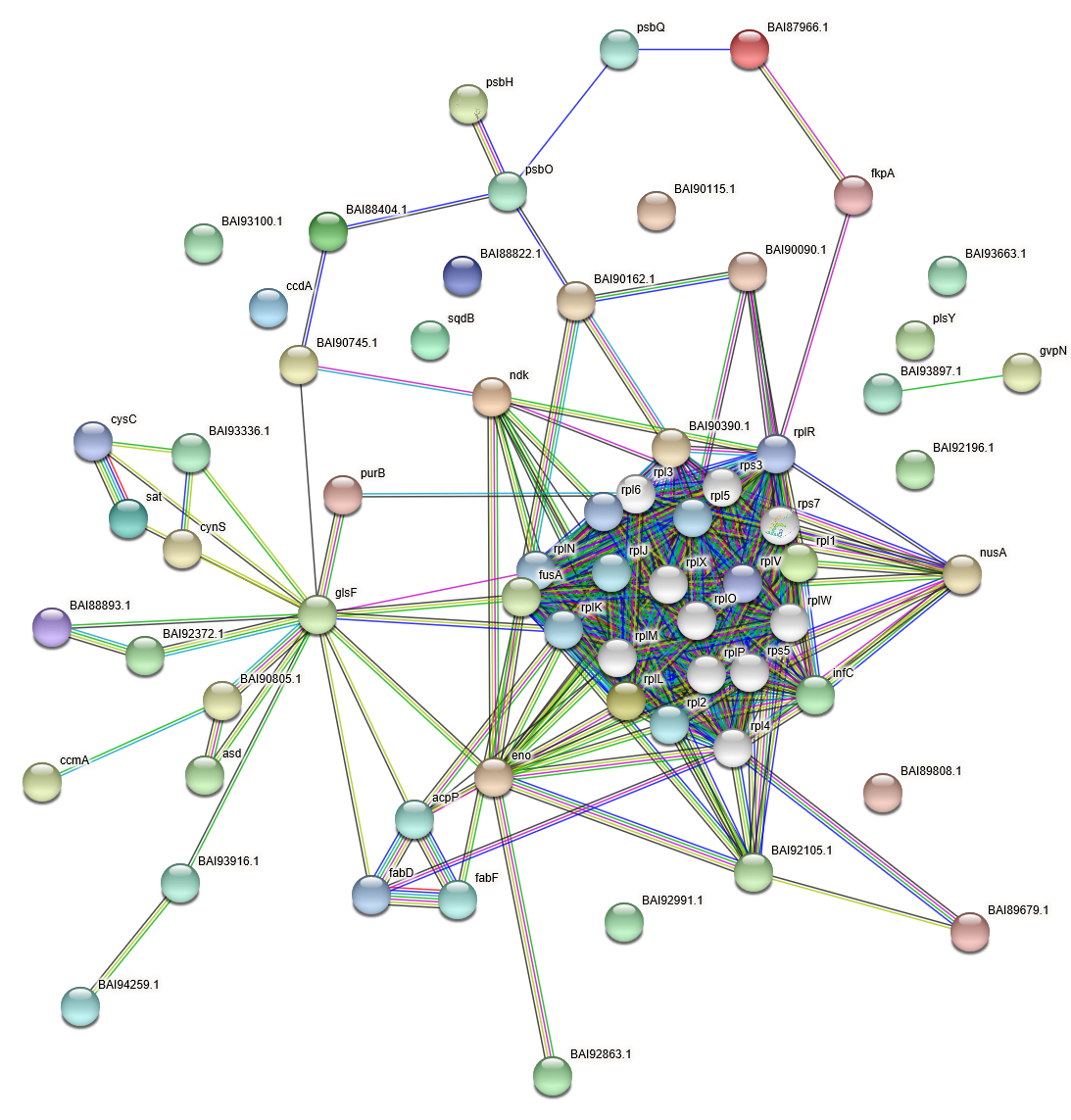
**

| **node** | **identifier** | **annotation** |
| --- | --- | --- |
| BAI87966.1 | NIES39_A01270 | Hypothetical protein |
| cysC | NIES39_A01770 | Adenylylsulfate kinase; Catalyzes the synthesis of activated sulfate |
| ndk | NIES39_A03300 | Nucleoside diphosphate kinase; Major role in the synthesis of nucleoside triphosphates other than ATP. The ATP gamma phosphate is transferred to the NDP beta phosphate via a ping-pong mechanism, using a phosphorylated active-site intermediate |
| rplL | NIES39_A03620 | 50S ribosomal protein L7/L12; Forms part of the ribosomal stalk which helps the ribosome interact with GTP-bound translation factors. Is thus essential for accurate translation |
| rplJ | NIES39_A03630 | 50S ribosomal protein L10; Forms part of the ribosomal stalk, playing a central role in the interaction of the ribosome with GTP-bound translation factors |
| rpl1 | NIES39_A03640 | 50S ribosomal protein L1; Binds directly to 23S rRNA. The L1 stalk is quite mobile in the ribosome, and is involved in E site tRNA release |
| rplK | NIES39_A03650 | 50S ribosomal protein L11; Forms part of the ribosomal stalk which helps the ribosome interact with GTP-bound translation factors |
| BAI88404.1 | NIES39_A05660 | Hypothetical protein |
| sqdB | NIES39_A06480 | Sulfolipid (UDP-sulfoquinovose) biosynthesis protein |
| sat | NIES39_A06770 | Sulfate adenylyltransferase |
| ccdA | NIES39_A07940 | Putative c-type cytochrome biogenesis protein CcdA |
| BAI88822.1 | NIES39_B00650 | Hypothetical protein |
| BAI88893.1 | NIES39_C00230 | Aminopeptidase P |
| fkpA | NIES39_D00650 | FKBP-type peptidyl-prolyl cis-trans isomerase |
| BAI89679.1 | NIES39_D02590 | ATP-dependent Clp protease proteolytic subunit; Cleaves peptides in various proteins in a process that requires ATP hydrolysis. Has a chymotrypsin-like activity. Plays a major role in the degradation of misfolded proteins |
| purB | NIES39_D02980 | Adenylosuccinate lyase |
| BAI89808.1 | NIES39_D03900 | Hypothetical protein |
| rpl3 | NIES39_D06390 | 50S ribosomal protein L3; One of the primary rRNA binding proteins, it binds directly near the 3'-end of the 23S rRNA, where it nucleates assembly of the 50S subunit |
| rpl4 | NIES39_D06400 | 50S ribosomal protein L4; Forms part of the polypeptide exit tunnel |
| rplW | NIES39_D06410 | 50S ribosomal protein L23; One of the early assembly proteins it binds 23S rRNA. One of the proteins that surrounds the polypeptide exit tunnel on the outside of the ribosome. Forms the main docking site for trigger factor binding to the ribosome |
| rpl2 | NIES39_D06420 | 50S ribosomal protein L2; One of the primary rRNA binding proteins. Required for association of the 30S and 50S subunits to form the 70S ribosome, for tRNA binding and peptide bond formation. It has been suggested to have peptidyltransferase activity; this is somewhat controversial. Makes several contacts with the 16S rRNA in the 70S ribosome |
| rplV | NIES39_D06440 | 50S ribosomal protein L22; The globular domain of the protein is located near the polypeptide exit tunnel on the outside of the subunit, while an extended beta-hairpin is found that lines the wall of the exit tunnel in the center of the 70S ribosome |
| rps3 | NIES39_D06450 | 30S ribosomal protein S3; Binds the lower part of the 30S subunit head. Binds mRNA in the 70S ribosome, positioning it for translation |
| rplP | NIES39_D06460 | 50S ribosomal protein L16; Binds 23S rRNA and is also seen to make contacts with the A and possibly P site tRNAs |
| rplN | NIES39_D06490 | 50S ribosomal protein L14; Binds to 23S rRNA. Forms part of two intersubunit bridges in the 70S ribosome |
| rplX | NIES39_D06500 | 50S ribosomal protein L24; One of the proteins that surrounds the polypeptide exit tunnel on the outside of the subunit |
| rpl5 | NIES39_D06510 | 50S ribosomal protein L5; This is 1 of the proteins that binds and probably mediates the attachment of the 5S RNA into the large ribosomal subunit, where it forms part of the central protuberance. In the 70S ribosome it contacts protein S13 of the 30S subunit (bridge B1b), connecting the 2 subunits; this bridge is implicated in subunit movement. Contacts the P site tRNA; the 5S rRNA and some of its associated proteins might help stabilize positioning of ribosome-bound tRNAs |
| rpl6 | NIES39_D06530 | 50S ribosomal protein L6; This protein binds to the 23S rRNA, and is important in its secondary structure. It is located near the subunit interface in the base of the L7/L12 stalk, and near the tRNA binding site of the peptidyltransferase center |
| rplR | NIES39_D06540 | 50S ribosomal protein L18; This is one of the proteins that binds and probably mediates the attachment of the 5S RNA into the large ribosomal subunit, where it forms part of the central protuberance |
| rps5 | NIES39_D06550 | 30S ribosomal protein S5; Located at the back of the 30S subunit body where it stabilizes the conformation of the head with respect to the body |
| rplO | NIES39_D06560 | 50S ribosomal protein L15; Binds to the 23S rRNA |
| rplM | NIES39_D06660 | 50S ribosomal protein L13; This protein is one of the early assembly proteins of the 50S ribosomal subunit, although it is not seen to bind rRNA by itself. It is important during the early stages of 50S assembly |
| BAI90090.1 | NIES39_D06730 | ABC transporter ATP-binding protein |
| BAI90115.1 | NIES39_D06980 | Hypothetical protein |
| eno | NIES39_D07330 | Enolase; Catalyzes the reversible conversion of 2- phosphoglycerate into phosphoenolpyruvate. It is essential for the degradation of carbohydrates via glycolysis |
| BAI90162.1 | NIES39_D07450 | Hypothetical protein |
| BAI90390.1 | NIES39_E01580 | RNA-binding protein |
| nusA | NIES39_E01730 | Transcription termination factor NusA; Participates in both transcription termination and antitermination |
| cynS | NIES39_E04340 | Cyanate lyase; Catalyzes the reaction of cyanate with bicarbonate to produce ammonia and carbon dioxide |
| BAI90745.1 | NIES39_F00520 | Hypothetical protein |
| BAI90805.1 | NIES39_G00210 | Aspartate aminotransferase |
| gvpN | NIES39_H01080 | Gas vesicle protein GvpN |
| ccmA | NIES39_J02310 | Phospho-2-dehydro-3-deoxyheptonate aldolase |
| psbH | NIES39_J03950 | Photosystem II PsbH protein, PSII-H |
| fusA | NIES39_J05470 | Translation elongation factor EF-G; Catalyzes the GTP-dependent ribosomal translocation step during translation elongation. During this step, the ribosome changes from the pre-translocational (PRE) to the post- translocational (POST) state as the newly formed A-site-bound peptidyl-tRNA and P-site-bound deacylated tRNA move to the P and E sites, respectively. Catalyzes the coordinated movement of the two tRNA molecules, the mRNA and conformational changes in the ribosome |
| rps7 | NIES39_J05480 | 30S ribosomal protein S7; One of the primary rRNA binding proteins, it binds directly to 16S rRNA where it nucleates assembly of the head domain of the 30S subunit. Is located at the subunit interface close to the decoding center, probably blocks exit of the E-site tRNA |
| glsF | NIES39_J05540 | Ferredoxin-dependent glutamate synthase |
| plsY | NIES39_K03280 | Hypothetical protein; Catalyzes the transfer of an acyl group from acyl- phosphate (acyl-PO(4)) to glycerol-3-phosphate (G3P) to form lysophosphatidic acid (LPA). This enzyme utilizes acyl-phosphate as fatty acyl donor, but not acyl-CoA or acyl-ACP |
| BAI92105.1 | NIES39_K04600 | Cell division protein FtsH; Acts as a processive, ATP-dependent zinc metallopeptidase for both cytoplasmic and membrane proteins. Plays a role in the quality control of integral membrane proteins |
| asd | NIES39_L00080 | Aspartate-semialdehyde dehydrogenase; Catalyzes the NADPH-dependent formation of L-aspartate- semialdehyde (L-ASA) by the reductive dephosphorylation of L- aspartyl-4-phosphate |
| BAI92196.1 | NIES39_L00350 | acetyl-CoA acetyltransferase |
| BAI92372.1 | NIES39_L02120 | Hypothetical protein |
| BAI92863.1 | NIES39_M00250 | Peroxiredoxin |
| infC | NIES39_M00310 | Translation initiation factor IF-3; IF-3 binds to the 30S ribosomal subunit and shifts the equilibrum between 70S ribosomes and their 50S and 30S subunits in favor of the free subunits, thus enhancing the availability of 30S subunits on which protein synthesis initiation begins |
| BAI92991.1 | NIES39_M01540 | Hypothetical protein |
| BAI93100.1 | NIES39_M02630 | Probable protein phosphatase |
| fabD | NIES39_N01200 | Malonyl coenzyme A-acyl carrier protein transacylase |
| BAI93336.1 | NIES39_O00850 | Probable nitrate ABC transport system ATP-binding protein |
| BAI93663.1 | NIES39_O04160 | Cytosine-specific methyltransferase |
| psbO | NIES39_O06150 | Photosystem II manganese-stabilizing protein precursor |
| BAI93897.1 | NIES39_O06510 | Transglutaminase-like domain |
| BAI93916.1 | NIES39_O06700 | Hypothetical protein |
| psbQ | NIES39_Q00210 | psbQ protein |
| acpP | NIES39_Q02310 | Acyl carrier protein; Carrier of the growing fatty acid chain in fatty acid biosynthesis |
| fabF | NIES39_Q02320 | 3-oxoacyl-[acyl-carrier-protein] synthase II; Catalyzes the condensation reaction of fatty acid synthesis by the addition to an acyl acceptor of two carbons from malonyl-ACP |
| BAI94259.1 | NIES39_Q02510 | NAD-dependent epimerase/dehydratase |

**(B)**


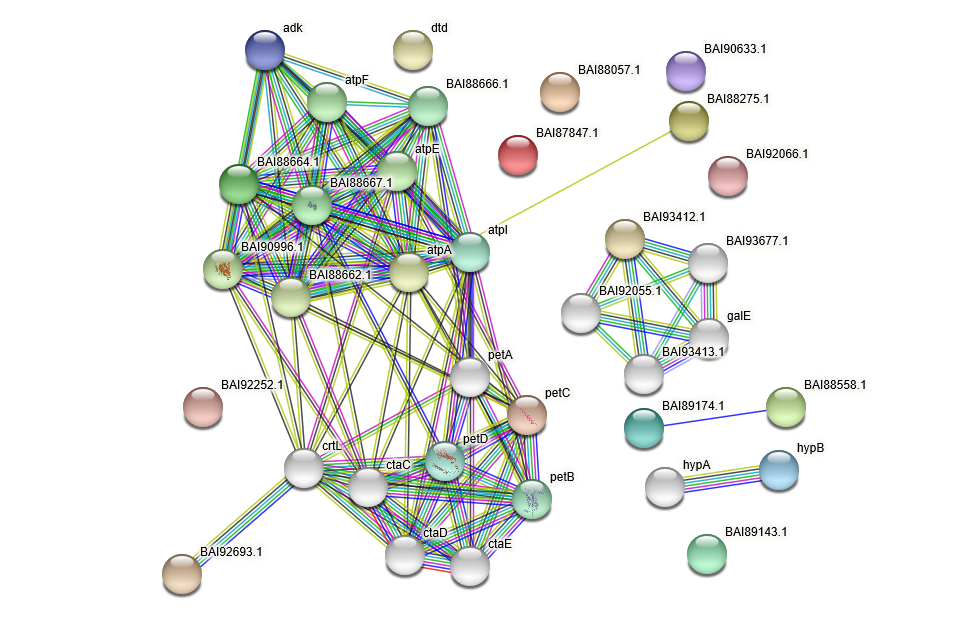


| **node** | **identifier** | **annotation** |
| --- | --- | --- |
| BAI87847.1 | NIES39_A00060 | Hypothetical protein |
| BAI88057.1 | NIES39_A02180 | Hypothetical protein |
| BAI88275.1 | NIES39_A04370 | Putative permease |
| BAI88558.1 | NIES39_A07200 | Hypothetical protein |
| BAI88662.1 | NIES39_A08240 | ATP synthase gamma chain; Produces ATP from ADP in the presence of a proton gradient across the membrane. The gamma chain is believed to be important in regulating ATPase activity and the flow of protons through the CF(0) complex |
| atpA | NIES39_A08250 | ATP synthase alpha chain; Produces ATP from ADP in the presence of a proton gradient across the membrane. The alpha chain is a regulatory subunit |
| BAI88664.1 | NIES39_A08260 | ATP synthase delta chain; F(1)F(0) ATP synthase produces ATP from ADP in the presence of a proton or sodium gradient. F-type ATPases consist of two structural domains, F(1) containing the extramembraneous catalytic core and F(0) containing the membrane proton channel, linked together by a central stalk and a peripheral stalk. During catalysis, ATP synthesis in the catalytic domain of F(1) is coupled via a rotary mechanism of the central stalk subunits to proton translocation |
| atpF | NIES39_A08270 | ATP synthase b chain; Component of the F(0) channel, it forms part of the peripheral stalk, linking F(1) to F(0) |
| BAI88666.1 | NIES39_A08280 | ATP synthase b' chain; Component of the F(0) channel, it forms part of the peripheral stalk, linking F(1) to F(0) |
| BAI88667.1 | NIES39_A08290 | ATP synthase c chain |
| atpI | NIES39_A08300 | ATP synthase a chain; Key component of the proton channel; it plays a direct role in the translocation of protons across the membrane |
| BAI89143.1 | NIES39_C02750 | Enoyl-[acyl-carrier-protein] reductase |
| BAI89174.1 | NIES39_C03060 | Hypothetical protein |
| crtL | NIES39_D01920 | Bifunctional lycopene cyclase/dioxygenase |
| hypB | NIES39_D03400 | Hydrogenase accessory protein HypB |
| hypA | NIES39_D03410 | Hydrogenase nickel insertion protein HypA; Probably plays a role in a hydrogenase nickel cofactor insertion step |
| galE | NIES39_D04350 | UDP-glucose 4-epimerase |
| adk | NIES39_D06580 | Adenylate kinase |
| BAI90633.1 | NIES39_E04060 | Hypothetical protein |
| atpE | NIES39_H00700 | ATP synthase epsilon chain; Produces ATP from ADP in the presence of a proton gradient across the membrane |
| BAI90996.1 | NIES39_H00710 | ATP synthase beta chain; Produces ATP from ADP in the presence of a proton gradient across the membrane. The catalytic sites are hosted primarily by the beta subunits |
| BAI92055.1 | NIES39_K04100 | UTP-glucose-1-phosphate uridylyltransferase |
| BAI92066.1 | NIES39_K04210 | Adenylate cyclase |
| BAI92252.1 | NIES39_L00910 | Hypothetical protein |
| petC | NIES39_L01870 | Cytochrome b6-f complex iron-sulfur subunit; Component of the cytochrome b6-f complex, which mediates electron transfer between photosystem II (PSII) and photosystem I (PSI), cyclic electron flow around PSI, and state transitions |
| petA | NIES39_L01880 | Apocytochrome f; Component of the cytochrome b6-f complex, which mediates electron transfer between photosystem II (PSII) and photosystem I (PSI), cyclic electron flow around PSI, and state transitions |
| BAI92693.1 | NIES39_L05360 | Hypothetical protein |
| petD | NIES39_M02410 | Cytochrome b6-f complex subunit IV; Component of the cytochrome b6-f complex, which mediates electron transfer between photosystem II (PSII) and photosystem I (PSI), cyclic electron flow around PSI, and state transitions |
| petB | NIES39_M02420 | Cytochrome b6; Component of the cytochrome b6-f complex, which mediates electron transfer between photosystem II (PSII) and photosystem I (PSI), cyclic electron flow around PSI, and state transitions |
| BAI93412.1 | NIES39_O01630 | UDP-glucose dehydrogenase |
| BAI93413.1 | NIES39_O01640 | Putative UDP-glucuronic acid decarboxylase |
| dtd | NIES39_O01770 | D-tyrosyl-tRNA(Tyr) deacylase; D-aminoacyl-tRNA deacylase with broad substrate specificity. By recycling D-aminoacyl-tRNA to D-amino acids and free tRNA molecules, this enzyme counteracts the toxicity associated with the formation of D-aminoacyl-tRNA entities in vivo |
| ctaE | NIES39_O03080 | Cytochrome c oxidase subunit III |
| ctaD | NIES39_O03090 | Cytochrome c oxidase subunit I; Cytochrome c oxidase is the component of the respiratory chain that catalyzes the reduction of oxygen to water. Subunits 1- 3 form the functional core of the enzyme complex. CO I is the catalytic subunit of the enzyme. Electrons originating in cytochrome c are transferred via the copper A center of subunit 2 and heme A of subunit 1 to the bimetallic center formed by heme A3 and copper B |
| ctaC | NIES39_O03100 | Cytochrome c oxidase subunit II; Subunits I and II form the functional core of the enzyme complex. Electrons originating in cytochrome c are transferred via heme a and Cu(A) to the binuclear center formed by heme a3 and Cu(B) |
| BAI93677.1 | NIES39_O04300 | Nucleotide sugar epimerase |

**(C)**

**
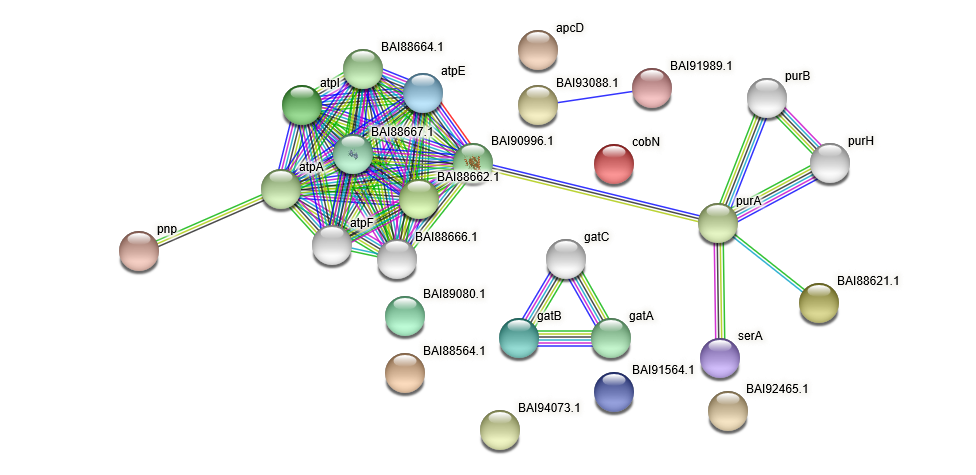
**

| **node** | **identifier** | **annotation** |
| --- | --- | --- |
| cobN | NIES39_A02240 | Cobalamin biosynthetic protein CobN |
| BAI88564.1 | NIES39_A07260 | Hypothetical protein; Specifically methylates the N3 position of the uracil ring of uridine 1498 (m3U1498) in 16S rRNA. Acts on the fully assembled 30S ribosomal subunit |
| BAI88621.1 | NIES39_A07830 | L-asparaginase |
| BAI88662.1 | NIES39_A08240 | ATP synthase gamma chain; Produces ATP from ADP in the presence of a proton gradient across the membrane. The gamma chain is believed to be important in regulating ATPase activity and the flow of protons through the CF(0) complex |
| atpA | NIES39_A08250 | ATP synthase alpha chain; Produces ATP from ADP in the presence of a proton gradient across the membrane. The alpha chain is a regulatory subunit |
| BAI88664.1 | NIES39_A08260 | ATP synthase delta chain; F(1)F(0) ATP synthase produces ATP from ADP in the presence of a proton or sodium gradient. F-type ATPases consist of two structural domains, F(1) containing the extramembraneous catalytic core and F(0) containing the membrane proton channel, linked together by a central stalk and a peripheral stalk. During catalysis, ATP synthesis in the catalytic domain of F(1) is coupled via a rotary mechanism of the central stalk subunits to proton translocation |
| atpF | NIES39_A08270 | ATP synthase b chain; Component of the F(0) channel, it forms part of the peripheral stalk, linking F(1) to F(0) |
| BAI88666.1 | NIES39_A08280 | ATP synthase b' chain; Component of the F(0) channel, it forms part of the peripheral stalk, linking F(1) to F(0) |
| BAI88667.1 | NIES39_A08290 | ATP synthase c chain |
| atpI | NIES39_A08300 | ATP synthase a chain; Key component of the proton channel; it plays a direct role in the translocation of protons across the membrane |
| BAI89080.1 | NIES39_C02120 | Hypothetical protein |
| purB | NIES39_D02980 | Adenylosuccinate lyase |
| purH | NIES39_D06170 | Bifunctional purine biosynthesis protein PurH |
| gatA | NIES39_E01380 | glutamyl-tRNA(Gln) amidotransferase subunit A; Allows the formation of correctly charged Gln-tRNA(Gln) through the transamidation of misacylated Glu-tRNA(Gln) in organisms which lack glutaminyl-tRNA synthetase. The reaction takes place in the presence of glutamine and ATP through an activated gamma-phospho-Glu-tRNA(Gln) |
| gatB | NIES39_E03060 | glutamyl-tRNA(Gln) amidotransferase subunit B; Allows the formation of correctly charged Asn-tRNA(Asn) or Gln-tRNA(Gln) through the transamidation of misacylated Asp- tRNA(Asn) or Glu-tRNA(Gln) in organisms which lack either or both of asparaginyl-tRNA or glutaminyl-tRNA synthetases. The reaction takes place in the presence of glutamine and ATP through an activated phospho-Asp-tRNA(Asn) or phospho-Glu-tRNA(Gln) |
| atpE | NIES39_H00700 | ATP synthase epsilon chain; Produces ATP from ADP in the presence of a proton gradient across the membrane |
| BAI90996.1 | NIES39_H00710 | ATP synthase beta chain; Produces ATP from ADP in the presence of a proton gradient across the membrane. The catalytic sites are hosted primarily by the beta subunits |
| BAI91564.1 | NIES39_J05180 | DNA-binding protein HU |
| serA | NIES39_K02580 | D-3-phosphoglycerate dehydrogenase |
| BAI91989.1 | NIES39_K03430 | Hypothetical protein |
| pnp | NIES39_L00330 | Polyribonucleotide nucleotidyltransferase; Involved in mRNA degradation. Catalyzes the phosphorolysis of single-stranded polyribonucleotides processively in the 3'- to 5'-direction |
| apcD | NIES39_L00400 | allophycocyanin-B alpha subunit |
| BAI92465.1 | NIES39_L03080 | Probable phosphoketolase |
| gatC | NIES39_L04400 | glutamyl-tRNA(Gln) amidotransferase subunit C; Allows the formation of correctly charged Asn-tRNA(Asn) or Gln-tRNA(Gln) through the transamidation of misacylated Asp- tRNA(Asn) or Glu-tRNA(Gln) in organisms which lack either or both of asparaginyl-tRNA or glutaminyl-tRNA synthetases. The reaction takes place in the presence of glutamine and ATP through an activated phospho-Asp-tRNA(Asn) or phospho-Glu-tRNA(Gln) |
| BAI93088.1 | NIES39_M02510 | DUF6 transmembrane protein |
| BAI94073.1 | NIES39_Q00650 | Phosphoenolpyruvate synthase; Catalyzes the phosphorylation of pyruvate to phosphoenolpyruvate |
| purA | NIES39_R00490 | Adenylosuccinate synthetase; Plays an important role in the de novo pathway of purine nucleotide biosynthesis. Catalyzes the first committed step in the biosynthesis of AMP from IMP |

**(D)**

**
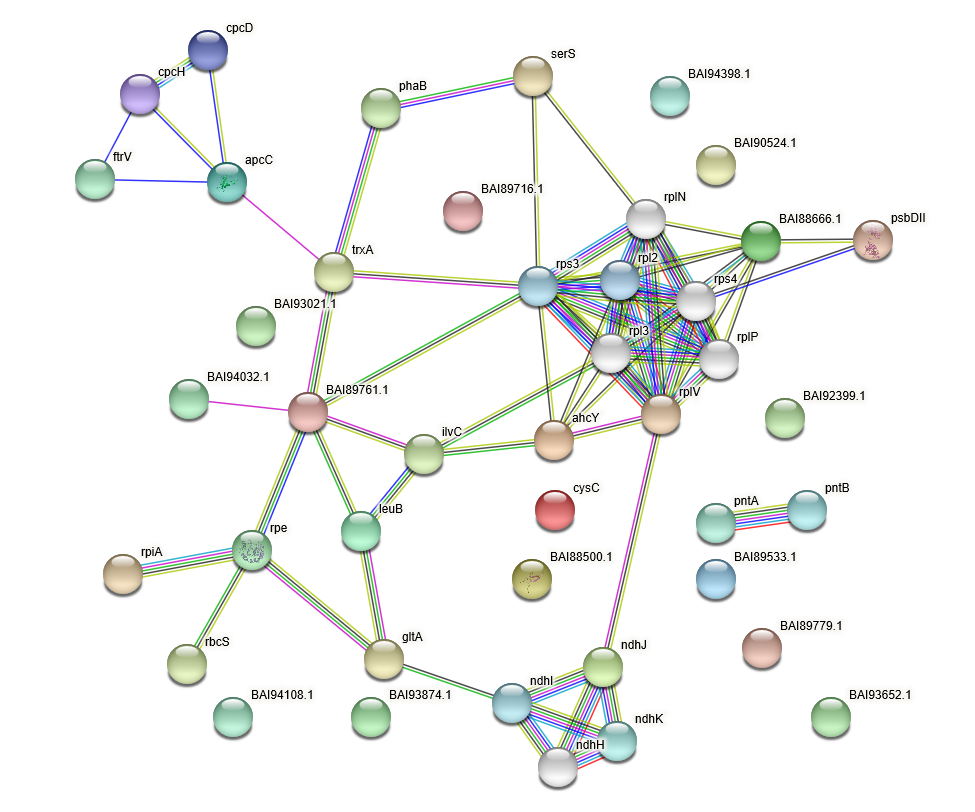
**

| **node** | **identifier** | **annotation** |
| --- | --- | --- |
| cysC | NIES39_A01770 | Adenylylsulfate kinase; Catalyzes the synthesis of activated sulfate |
| ahcY | NIES39_A04050 | Adenosylhomocysteinase; May play a key role in the regulation of the intracellular concentration of adenosylhomocysteine |
| BAI88500.1 | NIES39_A06620 | Type I restriction-modification system R subunit |
| ndhK | NIES39_A08130 | NADH dehydrogenase subunit K; NDH-1 shuttles electrons from an unknown electron donor, via FMN and iron-sulfur (Fe-S) centers, to quinones in the respiratory and/or the photosynthetic chain. The immediate electron acceptor for the enzyme in this species is believed to be plastoquinone. Couples the redox reaction to proton translocation, and thus conserves the redox energy in a proton gradient. Cyanobacterial NDH-1 also plays a role in inorganic carbon- concentration |
| ndhJ | NIES39_A08140 | NADH dehydrogenase subunit J; NDH-1 shuttles electrons from an unknown electron donor, via FMN and iron-sulfur (Fe-S) centers, to quinones in the respiratory and/or the photosynthetic chain. The immediate electron acceptor for the enzyme in this species is believed to be plastoquinone. Couples the redox reaction to proton translocation, and thus conserves the redox energy in a proton gradient. Cyanobacterial NDH-1 also plays a role in inorganic carbon- concentration |
| BAI88666.1 | NIES39_A08280 | ATP synthase b' chain; Component of the F(0) channel, it forms part of the peripheral stalk, linking F(1) to F(0) |
| leuB | NIES39_B00890 | 3-isopropylmalate dehydrogenase; Catalyzes the oxidation of 3-carboxy-2-hydroxy-4- methylpentanoate (3-isopropylmalate) to 3-carboxy-4-methyl-2- oxopentanoate. The product decarboxylates to 4-methyl-2 oxopentanoate |
| apcC | NIES39_C04520 | Phycobilisome small core linker polypeptide; Rod linker protein, associated with allophycocyanin. Linker polypeptides determine the state of aggregation and the location of the disk-shaped phycobiliprotein units within the phycobilisome and modulate their spectroscopic properties in order to mediate a directed and optimal energy transfer |
| BAI89533.1 | NIES39_D01130 | Hypothetical protein |
| cpcD | NIES39_D02020 | Phycocyanin associated linker protein |
| cpcH | NIES39_D02040 | Phycobilisome rod linker polypeptide CpcH |
| BAI89716.1 | NIES39_D02960 | Putative endodeoxyribonuclease |
| BAI89761.1 | NIES39_D03420 | Glyceraldehyde-3-phosphate dehydrogenase |
| BAI89779.1 | NIES39_D03600 | Hypothetical protein |
| psbDII | NIES39_D03780 | Photosystem II reaction center D2 protein; Photosystem II (PSII) is a light-driven water: plastoquinone oxidoreductase that uses light energy to abstract electrons from H(2)O, generating O(2) and a proton gradient subsequently used for ATP formation. It consists of a core antenna complex that captures photons, and an electron transfer chain that converts photonic excitation into a charge separation. The D1/D2 (PsbA/PsbA) reaction center heterodimer binds P680, the primary electron donor of PSII as well as several subsequent electron acceptors. D2 is needed for assembly of a stable PSII [...] |
| rpl3 | NIES39_D06390 | 50S ribosomal protein L3; One of the primary rRNA binding proteins, it binds directly near the 3'-end of the 23S rRNA, where it nucleates assembly of the 50S subunit |
| rpl2 | NIES39_D06420 | 50S ribosomal protein L2; One of the primary rRNA binding proteins. Required for association of the 30S and 50S subunits to form the 70S ribosome, for tRNA binding and peptide bond formation. It has been suggested to have peptidyltransferase activity; this is somewhat controversial. Makes several contacts with the 16S rRNA in the 70S ribosome |
| rplV | NIES39_D06440 | 50S ribosomal protein L22; The globular domain of the protein is located near the polypeptide exit tunnel on the outside of the subunit, while an extended beta-hairpin is found that lines the wall of the exit tunnel in the center of the 70S ribosome |
| rps3 | NIES39_D06450 | 30S ribosomal protein S3; Binds the lower part of the 30S subunit head. Binds mRNA in the 70S ribosome, positioning it for translation |
| rplP | NIES39_D06460 | 50S ribosomal protein L16; Binds 23S rRNA and is also seen to make contacts with the A and possibly P site tRNAs |
| rplN | NIES39_D06490 | 50S ribosomal protein L14; Binds to 23S rRNA. Forms part of two intersubunit bridges in the 70S ribosome |
| rpiA | NIES39_E02570 | Ribose-5-phosphate isomerase A; Catalyzes the reversible conversion of ribose-5- phosphate to ribulose 5-phosphate |
| serS | NIES39_E02670 | seryl-tRNA synthetase; Catalyzes the attachment of serine to tRNA(Ser). Is also able to aminoacylate tRNA(Sec) with serine, to form the misacylated tRNA L-seryl-tRNA(Sec), which will be further converted into selenocysteinyl-tRNA(Sec) |
| ndhI | NIES39_E02850 | NADH dehydrogenase subunit I; NDH-1 shuttles electrons from an unknown electron donor, via FMN and iron-sulfur (Fe-S) centers, to quinones in the respiratory and/or the photosynthetic chain. The immediate electron acceptor for the enzyme in this species is believed to be plastoquinone. Couples the redox reaction to proton translocation, and thus conserves the redox energy in a proton gradient |
| gltA | NIES39_E02870 | Citrate synthase |
| BAI90524.1 | NIES39_E02970 | Two-component hybrid histidine kinase |
| trxA | NIES39_G00780 | Thioredoxin |
| rbcS | NIES39_K02830 | Ribulose 1,5-bisphosphate carboxylase/oxygenase small subunit |
| ilvC | NIES39_K02910 | Ketol-acid reductoisomerase |
| phaB | NIES39_L00360 | PHA-specific acetoacetyl-CoA reductase |
| BAI92399.1 | NIES39_L02390 | Peptidyl-prolyl cis-trans isomerase |
| BAI93021.1 | NIES39_M01840 | Hypothetical protein |
| BAI93652.1 | NIES39_O04050 | Hypothetical protein |
| rps4 | NIES39_O05200 | 30S ribosomal protein S4; One of the primary rRNA binding proteins, it binds directly to 16S rRNA where it nucleates assembly of the body of the 30S subunit |
| BAI93874.1 | NIES39_O06280 | Probable peptidase |
| rpe | NIES39_O06990 | Ribulose-phosphate 3-epimerase |
| ndhH | NIES39_O07110 | NADH dehydrogenase subunit H; NDH-1 shuttles electrons from an unknown electron donor, via FMN and iron-sulfur (Fe-S) centers, to quinones in the respiratory and/or the photosynthetic chain. The immediate electron acceptor for the enzyme in this species is believed to be plastoquinone. Couples the redox reaction to proton translocation, and thus conserves the redox energy in a proton gradient. Cyanobacterial NDH-1 also plays a role in inorganic carbon- concentration |
| BAI94032.1 | NIES39_Q00240 | Hypothetical protein |
| ftrV | NIES39_Q00670 | Ferredoxin-thioredoxin reductase variable chain |
| BAI94108.1 | NIES39_Q01000 | Adenylate cyclase |
| pntB | NIES39_Q02580 | Putative nicotinamide nucleotide transhydrogenase beta subunit; The transhydrogenation between NADH and NADP is coupled to respiration and ATP hydrolysis and functions as a proton pump across the membrane |
| pntA | NIES39_Q02600 | Putative nicotinamide nucleotide transhydrogenase alpha subunit |
| BAI94398.1 | NIES39_R00890 | Hypothetical protein |
