# Supplementary material for: Revealing the key point of the temperature stress response of Arthrospira platensis C1 at the interconnection of C- and N- metabolism by proteome analyses and PPI networking
Source: BMC Mol Cell Biol. 2020 Jun 12;21:43. doi: 10.1186/s12860-020-00285-y (PMC7291507; doi:10.1186/s12860-020-00285-y)

Additional file 4

SPLC1_S230960 Histidine kinase

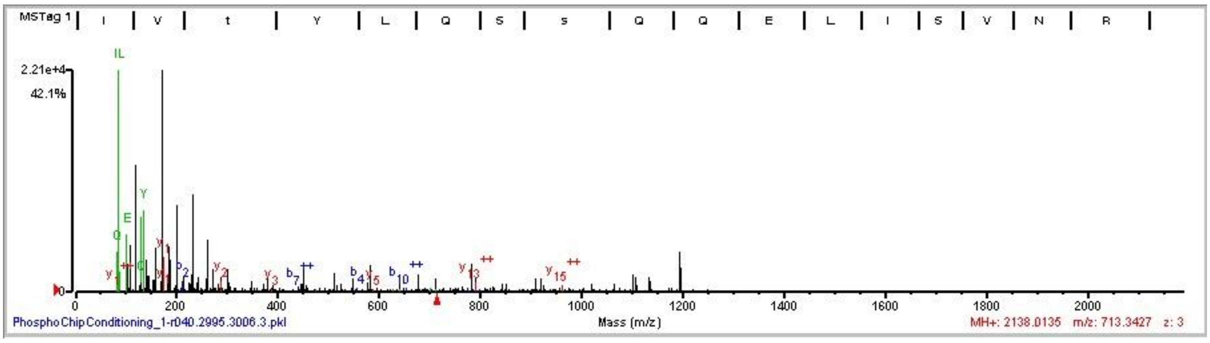

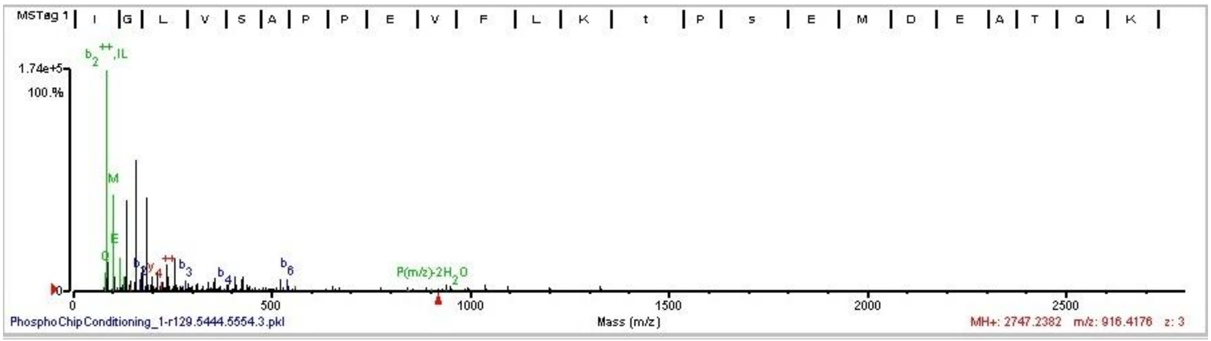

SPLC1_S082010 Histidine kinase

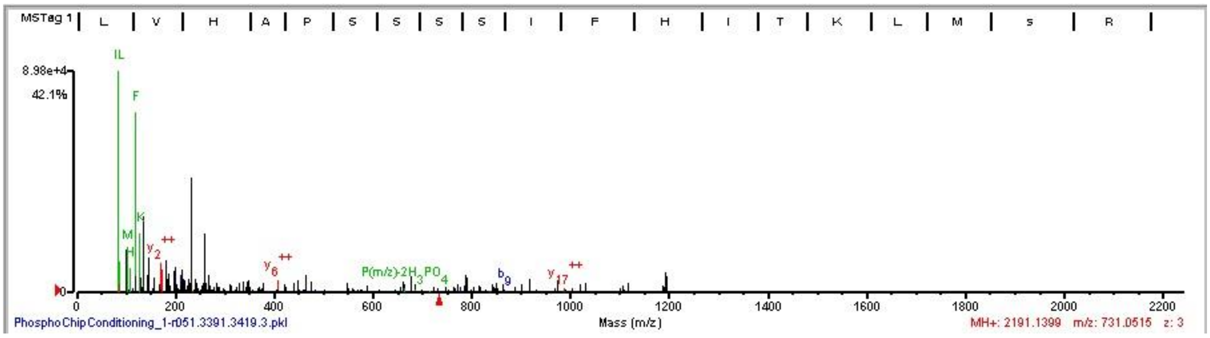

SPLC1_S041070 Histidine kinase

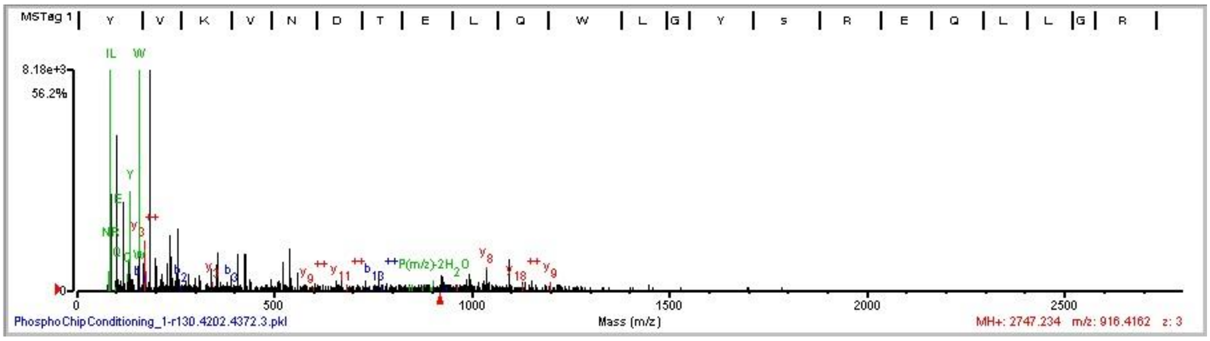

SPLC1_S520300 Histidine kinase

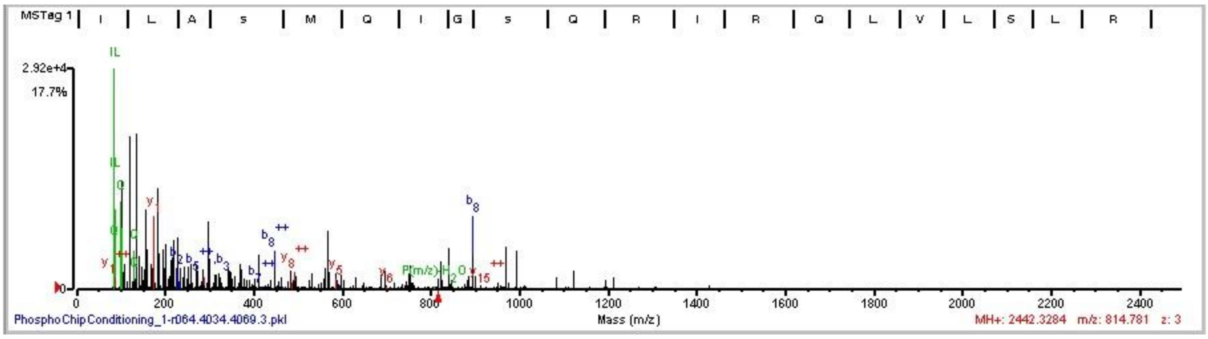

SPLC1_S270380 Glutamate synthase

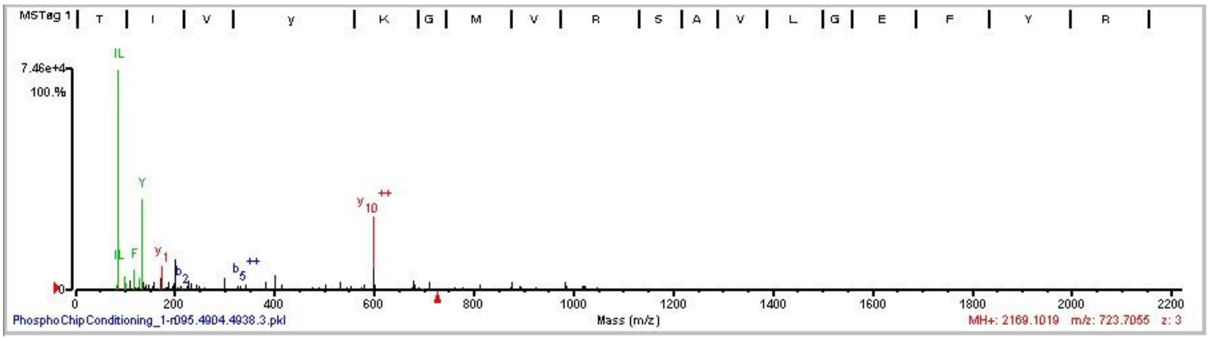

SPLC1_S240280 Serine/threonine protein kinase

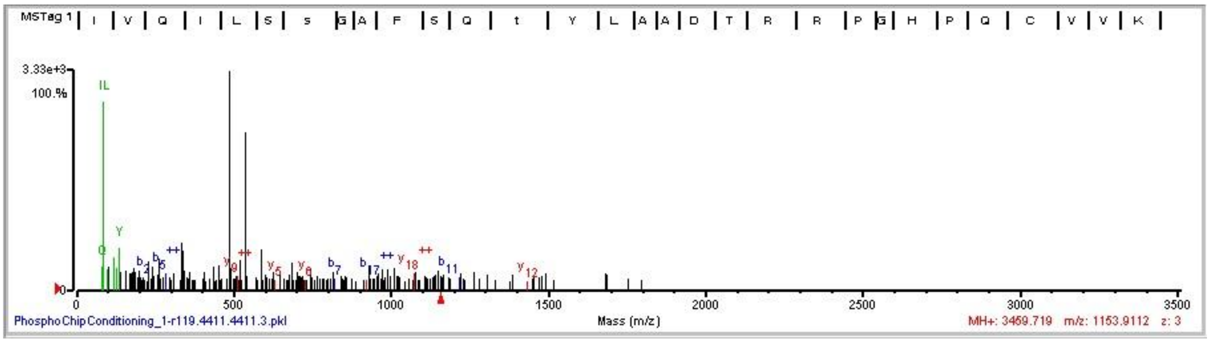

SPLC1_S032990 Serine/threonine protein kinase

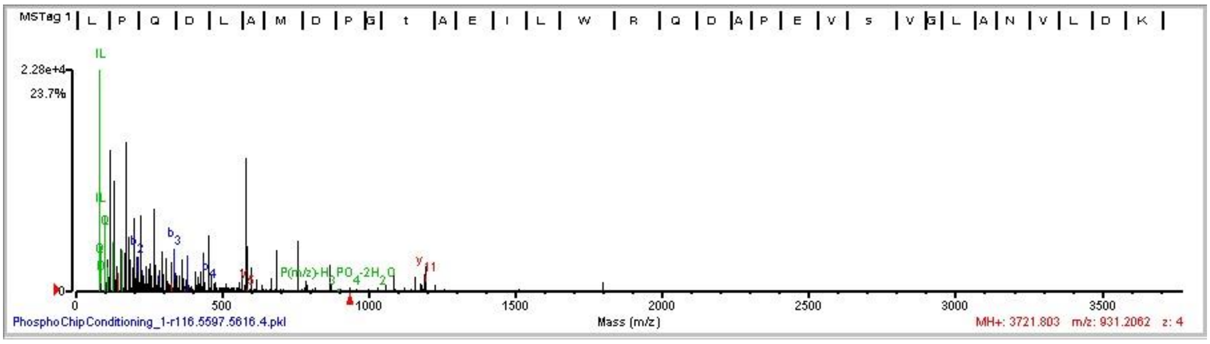

SPLC1_S240060 Ribulose-bisphosphate carboxylase large subunit

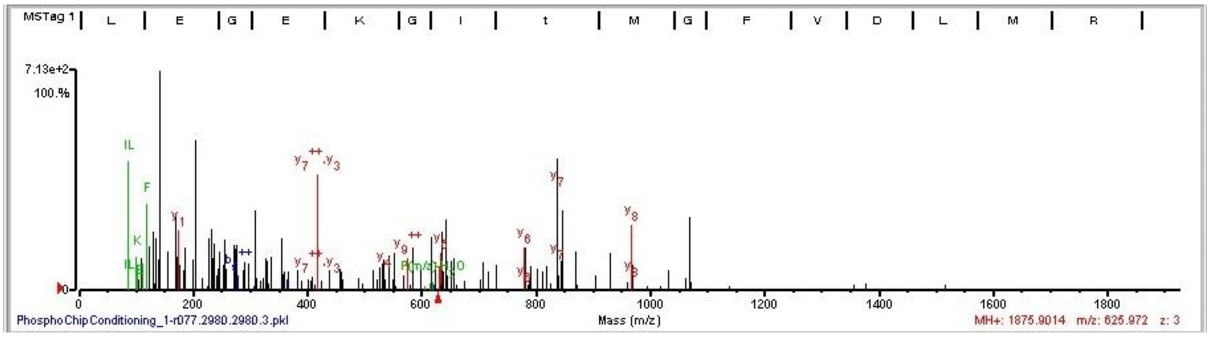

Supplement: Supplementary file 4 — Additional file 4. MS/MS spectra of some peptides of interest. [file 12860_2020_285_MOESM4_ESM.pdf]
