# Supplementary material for: Revealing the key point of the temperature stress response of Arthrospira platensis C1 at the interconnection of C- and N- metabolism by proteome analyses and PPI networking
Source: BMC Mol Cell Biol. 2020 Jun 12;21:43. doi: 10.1186/s12860-020-00285-y (PMC7291507; doi:10.1186/s12860-020-00285-y)
Supplement: Supplementary file 5 — Additional file 5. Protein sequence alignment of the eukaryotic type Ser/Thr kinase, PkA, and the Ser/Thr kinases of A. platensis C1; (A) SPLC1_S240280, SPLC1_S032990 and SPLC1_S280030, and (B) SPLC1_S200190, SPLC1_S208550, SPLC1_S532860, SPLC1_S580170 and SPLC1_S541370. The conserved protein domains are labeled. [file 12860_2020_285_MOESM5_ESM.pdf]

Additional file 5
(A)

```
. . . . . . . . . 10 . . . . . . . . . 20 . . . . . . . . . 30 . . . . . . . . . 40 . . . . . . . . . 50
                                       β1      P-loop      β2                 β3
PkA__PDBID_CHAI  ---PSQN-TA Q LD-QFDRIK TLGTGSFGRV MLVKHK--ES GNHYAMKILD
SPLC1_S240280    RQMEII--GQ L LDGRYRIVQ ILSSGAFSQT YLAADTRRPG HPQCVVKQLR
SPLC1_S032990    MQTPLNNQHQ L INGRYQVIQ SLGTGSFGEV FLAQDTQMPL QQHYVIKQLK
SPLC1_S280030    MLWQNG--QK L NNGNYMVDR ILGQGRFSVT YLAKEV--RT NGYVAIKTLN
Consistency      2214321025 6 465583556 4*75*5*636 5*75540043 325367*3*4

. . . . . . . . . 60 . . . . . . . . . 70 . . . . . . . . . 80 . . . . . . . . . 90 . . . . . . . . . 100
                 αB                     αC                          β4
PkA__PDBID_CHAI  KQK---V--- - -VKLKQIEH TLNEKRILQA VN-FPFLVKL EFSFKDN---
SPLC1_S240280    PPSNNSK--- - -ILKTALRL FRQEAEILEK LGRNDRIPLL LAYFEDN---
SPLC1_S032990    R-QVQPQ--- - -FNNVVKER FRQEAATLQT LGQHNQIPRL YAYFEQL---
SPLC1_S280030    PDASMLKDLK P SEREKYATK FYDEALKLSQ CQ-HPHIVKV REIFDEAVRE
Consistency      4141114000 0 033343343 635*633*64 5404438458 233*663000

. . . . . . . . . 110 . . . . . . . . . 120 . . . . . . . . . 130 . . . . . . . . . 140 . . . . . . . . . 150
                       β5                αD                 αE
PkA__PDBID_CHAI  ------SNLY M VMEYVAGGE MFSHLRRIGR FSEPHARFYA AQIVLTFEYL
SPLC1_S240280    ------NHFY L VEEFIEGHP LHKELIPGHP WKEDLVIELL EEILNILVFV
SPLC1_S032990    ------NKFY L VQEYIEGET LEAKVQREGK LSESAVREIL TSLLGVLQYI
SPLC1_S280030    GWFANTRYPC I VMDYIDGVS LGQRGEP--Q LPEKIALGYI QQIGSALISV
Consistency      0000005335 7 *58895*23 8244334024 44*3364256 4683257347

. . . . . . . . . 160 . . . . . . . . . 170 . . . . . . . . . 180 . . . . . . . . . 190 . . . . . . . . . 200
                 αE  β6  Catalytic loop  β7    β8  Mg-binding loop  β9  Activation loop
                         *                                                         *
PkA__PDBID_CHAI  HSLDLIYRDL K PENLLIDQQ -GYIQVTDFG FAKRVKG--- ------RTWT
SPLC1_S240280    HQNGVIHRDV N PSNLIRRKS DQALVLIDFG SVKAVTSQLA EENNQPMRTI
SPLC1_S032990    HSKQIVHRDI K PDNIILRQE DGKPVLIDFG AVKEAMSTQL NTQGNAVNSI
SPLC1_S280030    HQRGLLHRDL K PGNIMIRAG KQEAILIDFG LARSFDH--- ------PLTQ
Consistency      *634787**7 7 *4*874654 2433586*** 3684424000 0000002343

. . . . . . . . . 210 . . . . . . . . . 220 . . . . . . . . . 230 . . . . . . . . . 240 . . . . . . . . . 250
                 P+1 loop    αEF                 αF                         αG
PkA__PDBID_CHAI  LCGTPEYLAP E IILSKGYN- KAVDWWALGV LIYEMAAGYP PFFADQPIQI
SPLC1_S240280    ATGTPSYMPI E QFQGNPQF- N-SDLYAVGM IAIQALTGLE GSDLPKLQDP
SPLC1_S032990    IIGTPGFMAS E QAIGRPVY- S-SDLYSLGL TAIYMLTGKL PQDLAMDPGT
SPLC1_S280030    QITVDGFAPF E LYSHLEPKG PWTDVYSLAA TLYVLITGKK PESALDRHDT
Consistency      3467647552 * 433433220 305*477875 5553567*32 5335231144

. . . . . . . . . 260 . . . . . . . . . 270 . . . . . . . . . 280 . . . . . . . . . 290 . . . . . . . . . 300
                 αG                      αH
PkA__PDBID_CHAI  YEKIVSGKVR F PS-HFSSDL KDLLRNLLQV DLTKRFGNL- ----------
SPLC1_S240280    SLSISGEIAW R QRARVSNEL ASIIDKMVHH YYGKRYKSAI DAIAELNQLT
SPLC1_S032990    AEIL----WR Q DAPEVSVGL ANVLDKAVRS HAGDRYSSAQ EM--------
SPLC1_S280030    DSDVHL--TP P PVCTIPRLA KRLIRRSF-- MPWL------ ----------
Consistency      3428110022 2 431366326 5478454521 2233531320 1000000000

. . . . . . . . . 310 . . . . . . . . . 320 . . . . . . . . . 330 . . . . . . . . . 340 . . . . . . . . . 350
PkA__PDBID_CHAI  ---------- K NGVNDIKN- ---------- --------HK WFATTDWIAI
SPLC1_S240280    GKTGLVNPTS P NHPPYKTHK PRRDISLNRL WTVVGIAATG LVGLIVIFGL
SPLC1_S032990    ---------- L DALKSLTDR YIMDPNNTGL APQTKRGLLV LSTIVSLVVL
```

```
SPLC1_S280030    ---------- - --------- ---------- ---------- ----------
Consistency      0000000000 1 311111221 0001000001 0000000010 2112211214

                 .........360 .........370.........380.........390.........400
PkA__PDBID_CHAI  YQRKVEAP-- F IPKFKGPGD TSNFDDYEEE EIR-----VS IN---EKCGK
SPLC1_S240280    FQVLSRPDPV K SEAALKRGV ERLEAGDPEA AIKAFTRSIQ LFPDNSEAFR
SPLC1_S032990    GLGWRSLPYL W KPKVQAQSL TIGSLSNPKY NTDLVDYLRQ ELIPSNFIDF
SPLC1_S280030    ---------- - --------- ---------- ---------- ----------
Consistency      1200121201 0 122111131 2101011231 1210000013 1100021101

                 .........410 .........420.........430.........440.........450
PkA__PDBID_CHAI  EFTEF----- - --------- ---------- ---------- ----------
SPLC1_S240280    KRANAYYDLQ K YEQAIADYT QAIKLDPTNP DIYFNRSLAY HQMRDFGNAI
SPLC1_S032990    FLGKKVDLAI D GDHNLPYQE AKDRI----A KKEWDVVFTL SPIISVAAKD
SPLC1_S280030    ---------- - --------- ---------- ---------- ----------
Consistency      1112100000 0 010010000 0001100000 0000000000 0000000000

                 .........460 .........470.........480.........490.........500
PkA__PDBID_CHAI  ---------- - --------- ---------- ---------- ----------
SPLC1_S240280    NDLNQVIRLN P EDTDAFYQR GLAHYSQENY EAAILDYTEV IRRQPNNSEA
SPLC1_S032990    NSYTFAALMF P EGPPYYYSA LYVR-----A DSPIQSINDI TPSTVIALGG
SPLC1_S280030    ---------- - --------- ---------- ---------- ----------
Consistency      1000000010 1 100001100 0000000000 1101000011 0000000000

                 .........510 .........520.........530.........540.........550
PkA__PDBID_CHAI  ---------- - --------- ---------- ---------- ----------
SPLC1_S240280    YRARGSAHVK S GNLQAGMAD YTEAIRLNPE SAAAYYNRGR ARFHLGDYQG
SPLC1_S032990    FNSASSFYMP V --YDLYGKT LTVDMGHRGQ NIREMVRTGK ADLGAGALGD
SPLC1_S280030    ---------- - --------- ---------- ---------- ----------
Consistency      1010010000 0 000000000 0100000001 0000000011 1000010000

                 .........560 .........570.........580.........590.........600
PkA__PDBID_CHAI  ---------- - --------- ---------- ---------- ----------
SPLC1_S240280    ALADYNQVIS W EPDNAEAYG NRCSTYINLG NYEAAIESCS RSIQLNPTAM
SPLC1_S032990    TVKNYPDLRI I H--LSRAIP GAGVYLSPEL SESDRKVIER VLLNAPPDIQ
SPLC1_S280030    ---------- - --------- ---------- ---------- ----------
Consistency      0100100100 0 000010100 0000000000 0000000000 0010001000

                 .........610 .........620.........630.........640.........650
PkA__PDBID_CHAI  ---------- - --------- ---------- ---------- ----------
SPLC1_S240280    DYNNRCIAHL - NVQNYDAAI GDCTKAIELE -----PNNSK AHSNRGLVHS
SPLC1_S032990    KQANYGLGSE P DYTNFRGII RRTEEILVCS NFRQNPVNFF CGTGVGTVPT
SPLC1_S280030    ---------- - --------- ---------- ---------- ----------
Consistency      0001001000 0 000110001 0000001000 0000010100 0000010100

                 .........660 .........670.........680.........690.........700
PkA__PDBID_CHAI  ---------- - --------- ---------- ---------- ----------
SPLC1_S240280    LAENYEAAIA D YSQAISLNP NDAESYSNRA QAHAELGNYS EAIADYAQAI
SPLC1_S032990    TVNHGGNEIR G RVNGWRR-P TVDTVWLNLM AEGN----QL YRVVVSSQIL
SPLC1_S280030    ---------- - --------- ---------- ---------- ----------
Consistency      0000000010 0 000000001 0000000100 0000000000 0010001101

                 .........710 .........720.........730.........740.........750
PkA__PDBID_CHAI  ---------- - --------- ---------- ---------- ----------
SPLC1_S240280    RIRPNLAGAF Y GRGMV-RAS LGDRRGAISD FEQAGKLFLE QGLTGGFRDS
SPLC1_S032990    NQVPG-ATNL L ELQNKEIKV MGVVPNQIG- -DGILEL--- ------NIQN
SPLC1_S280030    ---------- - --------- ---------- ---------- ----------
Consistency      0001001000 0 000000000 1100000100 0100001000 0000000000
```

	. . . .
PkA__PDBID_CHAI	- - - - - - - -
SPLC1_S240280	Q Y Q I Q R L Q
SPLC1_S032990	S M E L E V L -
SPLC1_S280030	- - - - - - - -
**Consistency**	0 0 1 1 1 0 1 0

Additional file 5

(B)

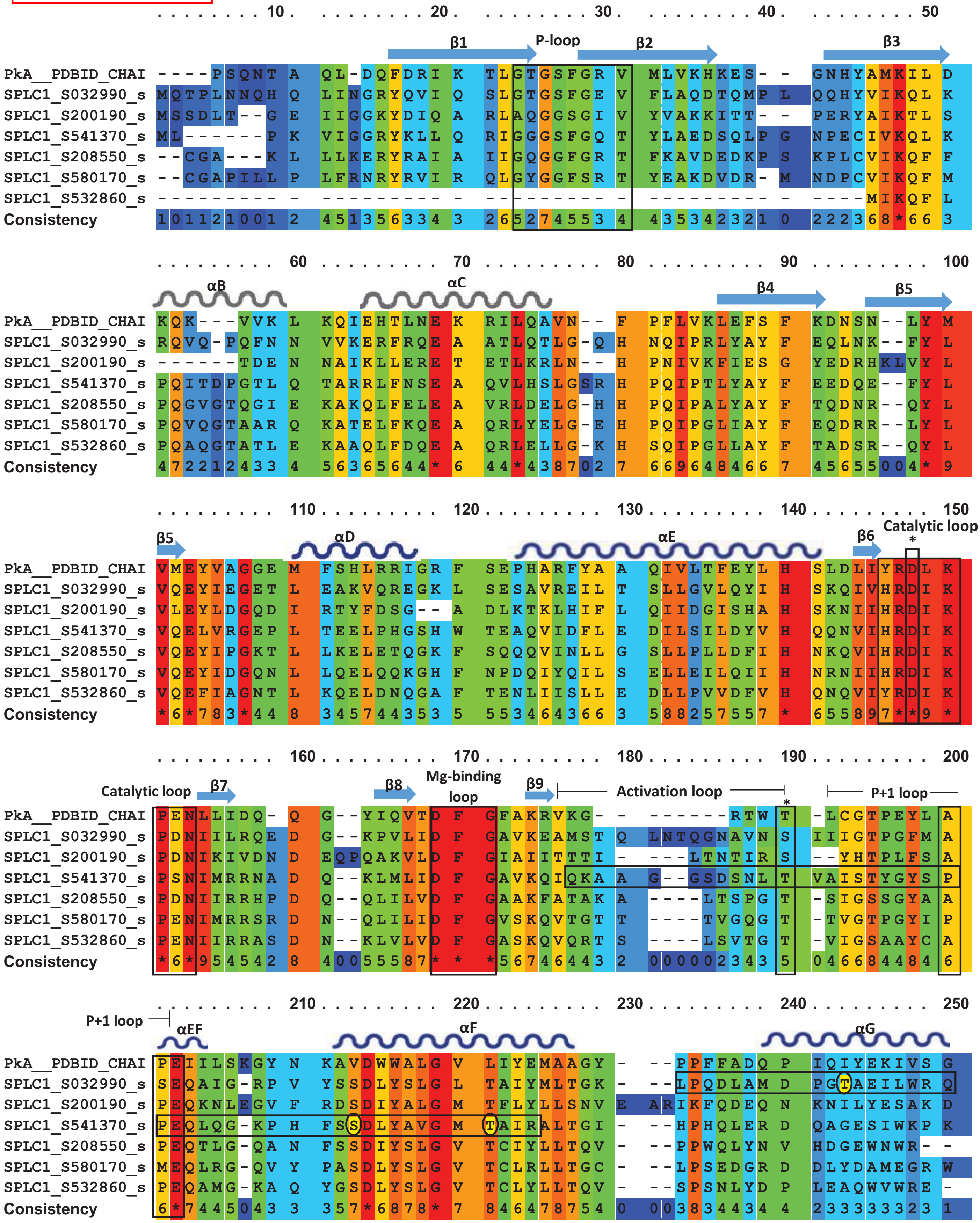

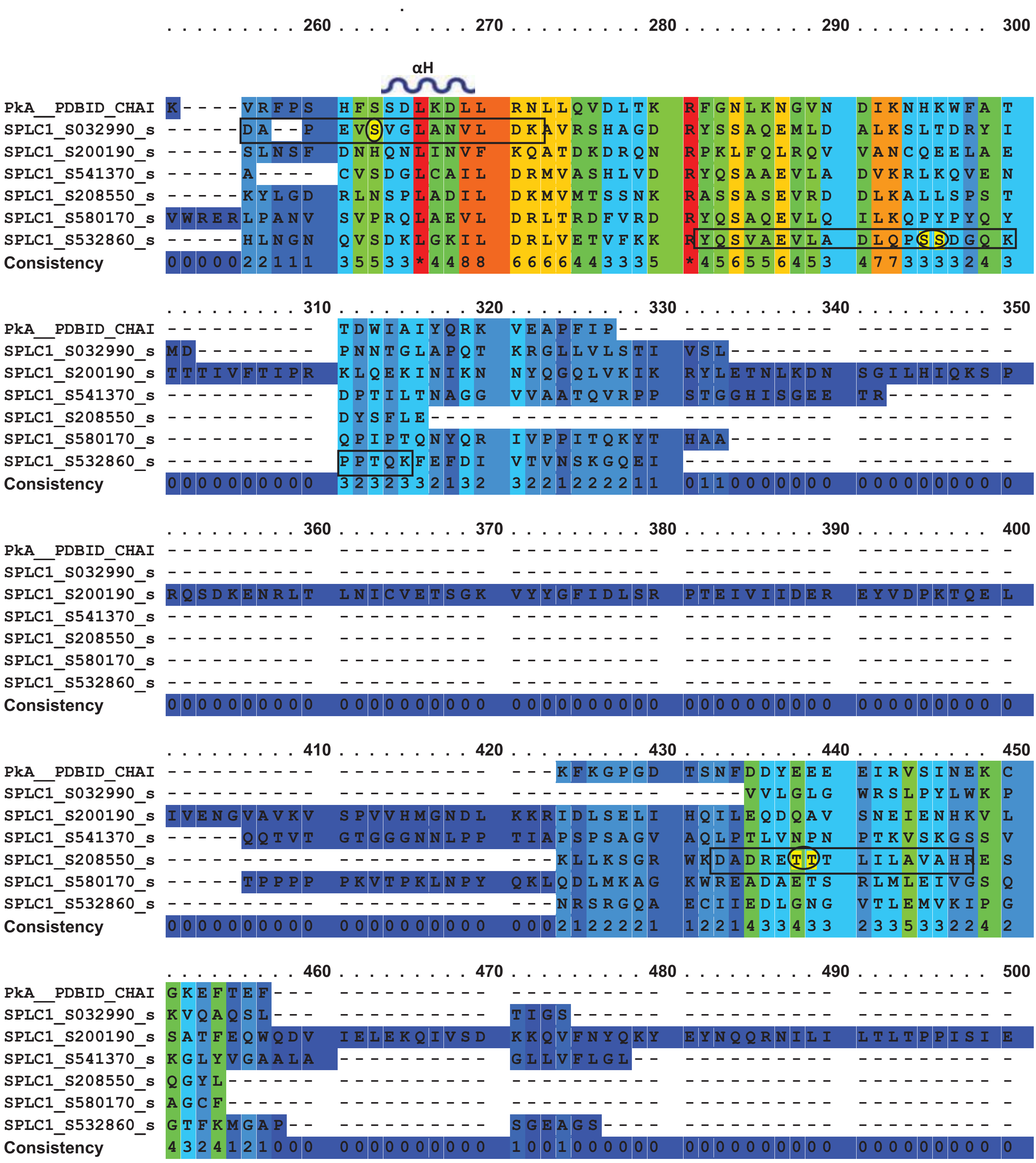
αH
.... 260 .... 270 .... 280 .... 290 300
PkA__PDBID_CHAI K----VRFPS HFSSDLKDLL RNLLQVDLTK RFGNLKNGVN DIKNHKWFAT
SPLC1_S032990_s -----DA--P EVSVGLANVL DKAVRSHAGD RYSSAQEMLD ALKSLTDRYI
SPLC1_S200190_s -----SLNSF DNHQNLINVF KQATDKDRQN RPKLFQLRQV VANCQEELAE
SPLC1_S541370_s -----A---- CVSDGLCAIL DRMVASHLVD RYQSAAEVLA DVKRLKQVEN
SPLC1_S208550_s -----KYLGD RLNSPLADIL DKMVMTSSNK RASSASEVRD DLKALLSPST
SPLC1_S580170_s VWRERLPANV SVPRQLAEVL DRLTRDFVRD RYQSAQEVLQ ILKQPYPYQY
SPLC1_S532860_s -----HLNGN QVSDKLGKIL DRLVETVFKK RYQSVAEVLA DLQPSSDGQK
Consistency 0000022111 35533*4488 6666443335 *456556453 4773333243
.... 310 .... 320 .... 330 .... 340 350
PkA__PDBID_CHAI ---------- TDWIAIYQRK VEAPFIP--- ---------- ----------
SPLC1_S032990_s MD-------- PNNTGLAPQT KRGLLVLSTI VSL------- ----------
SPLC1_S200190_s TTTIVFTIPR KLQEKINIKN NYQGQLVKIK RYLETNLKDN SGILHIQKSP
SPLC1_S541370_s ---------- DPTILTNAGG VVAATQVRPP STGGHISGEE TR--------
SPLC1_S208550_s ---------- DYSFLE---- ---------- ---------- ----------
SPLC1_S580170_s ---------- QPIPTQNYQR IVPPITQKYT HAA------- ----------
SPLC1_S532860_s ---------- PPTQKFEFDI VTVNSKGQEI ---------- ----------
Consistency 0000000000 3232332132 3221222211 0110000000 0000000000
.... 360 .... 370 .... 380 .... 390 400
PkA__PDBID_CHAI ---------- ---------- ---------- ---------- ----------
SPLC1_S032990_s ---------- ---------- ---------- ---------- ----------
SPLC1_S200190_s RQSDKENRLT LNICVETSGK VYYGFIDLSR PTEIVIIDER EYVDPKTQEL
SPLC1_S541370_s ---------- ---------- ---------- ---------- ----------
SPLC1_S208550_s ---------- ---------- ---------- ---------- ----------
SPLC1_S580170_s ---------- ---------- ---------- ---------- ----------
SPLC1_S532860_s ---------- ---------- ---------- ---------- ----------
Consistency 0000000000 0000000000 0000000000 0000000000 0000000000
.... 410 .... 420 .... 430 .... 440 450
PkA__PDBID_CHAI ---------- ---------- ---KFKGPGD TSNFDDYEEE EIRVSINEKC
SPLC1_S032990_s ---------- ---------- ---------- ----VVLGLG WRSLPYLWKP
SPLC1_S200190_s IVENGVAVKV SPVVHMGNDL KKRIDLSELI HQILEQDQAV SNEIENHKVL
SPLC1_S541370_s -----QQTVT GTGGGNNLPP TIAPSPSAGV AQLPTLVNPN PTKVSKGSSV
SPLC1_S208550_s ---------- ---------- ---KLLKSGR WKDADRETTT LILAVAHRES
SPLC1_S580170_s -----TPPPP PKVTPKLNPY QKLQDLMKAG KWREADAETS RLMLEIVGSQ
SPLC1_S532860_s ---------- ---------- ---NRSRGQA ECIIEDLGNG VTLEMVKIPG
Consistency 0000000000 0000000000 0002122221 1221433433 2335332242
.... 460 .... 470 .... 480 .... 490 500
PkA__PDBID_CHAI GKEFTEF--- ---------- ---------- ---------- ----------
SPLC1_S032990_s KVQAQSL--- ---------- TIGS------ ---------- ----------
SPLC1_S200190_s SATFEQWQDV IELEKQIVSD KKQVFNYQKY EYNQQRNILI LTLTPPISIE
SPLC1_S541370_s KGLYVGAALA ---------- GLLVFLGL-- ---------- ----------
SPLC1_S208550_s QGYL------ ---------- ---------- ---------- ----------
SPLC1_S580170_s AGCF------ ---------- ---------- ---------- ----------
SPLC1_S532860_s GTFKMGAP-- ---------- SGEAGS---- ---------- ----------
Consistency 4324121000 0000000000 1001000000 0000000000 0000000000

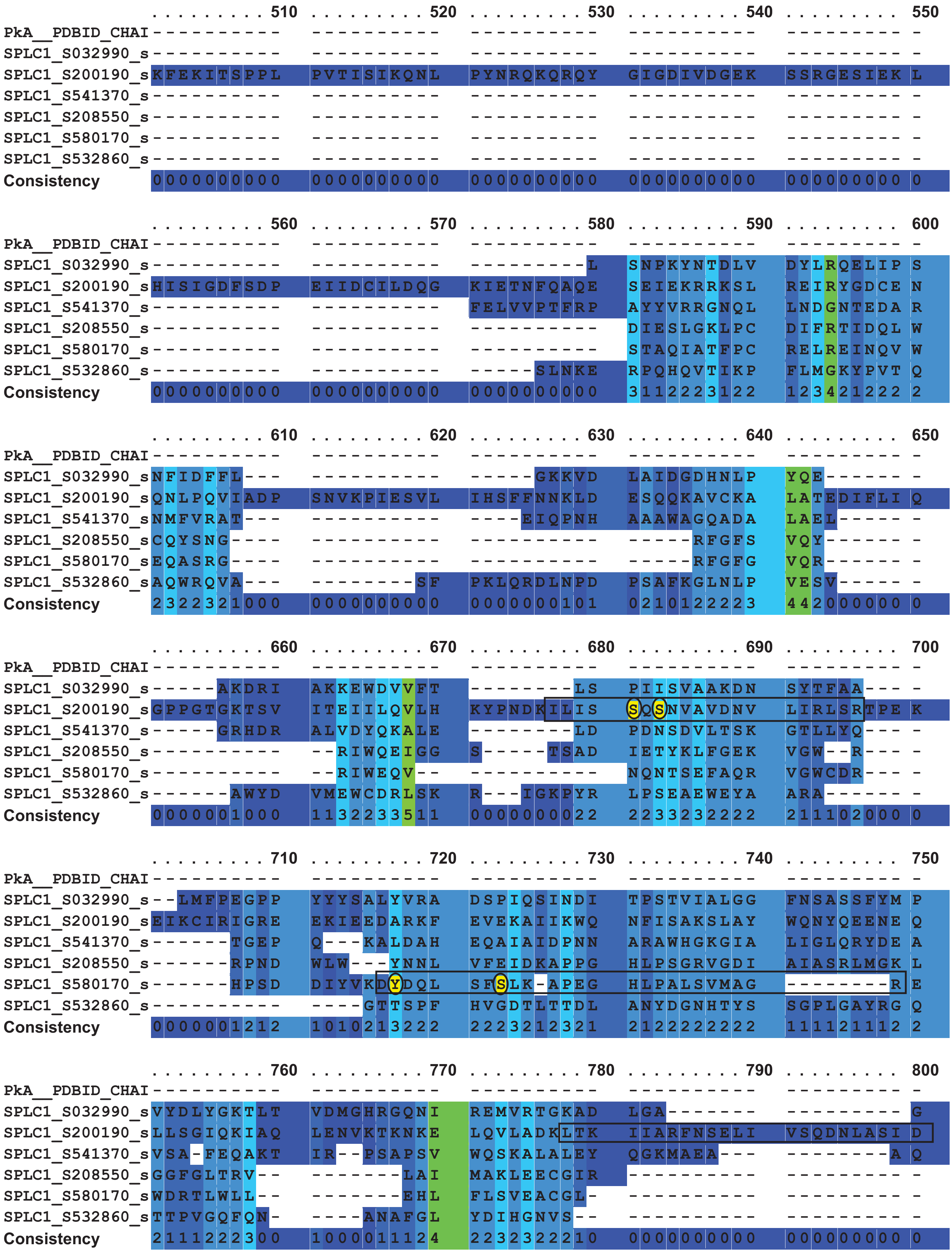
510 520 530 540 550
PkA__PDBID_CHAI ---------- ---------- ---------- ---------- ---------- -
SPLC1_S032990_s ---------- ---------- ---------- ---------- ---------- -
SPLC1_S200190_s KFEKITSPPL PVTISIKQNL PYNRQKQRQY GIGDIVDGEK SSRGESIEK L
SPLC1_S541370_s ---------- ---------- ---------- ---------- ---------- -
SPLC1_S208550_s ---------- ---------- ---------- ---------- ---------- -
SPLC1_S580170_s ---------- ---------- ---------- ---------- ---------- -
SPLC1_S532860_s ---------- ---------- ---------- ---------- ---------- -
Consistency 0000000000 0000000000 0000000000 0000000000 000000000 0
560 570 580 590 600
PkA__PDBID_CHAI ---------- ---------- ---------- ---------- ---------- -
SPLC1_S032990_s ---------- ---------- ---------L SNPKYNTDLV DYLRQELIP S
SPLC1_S200190_s HISIGDFSDP EIIDCILDQG KIETNFQAQE SEIEKRRKSL REIRYGDCE N
SPLC1_S541370_s ---------- ---------- FELVVPTFRP AYYVRRGNQL LNDGNTEDA R
SPLC1_S208550_s ---------- ---------- ---------- DIESLGKLPC DIFRTIDQL W
SPLC1_S580170_s ---------- ---------- ---------- STAQIATFPC RELREINQV W
SPLC1_S532860_s ---------- ---------- -----SLNKE RPQHQVTIKP FLMGKYPVT Q
Consistency 0000000000 0000000000 0000000000 3112223122 123421222 2
610 620 630 640 650
PkA__PDBID_CHAI ---------- ---------- ---------- ---------- ---------- -
SPLC1_S032990_s NFIDFFL--- ---------- -----GKKVD LAIDGDHNLP YQE------ -
SPLC1_S200190_s QNLPQVIADP SNVKPIESVL IHSFFNNKLD ESQQKAVCKA LATEDIFLI Q
SPLC1_S541370_s NMFVRAT--- ---------- ----EIQPNH AAAWAGQADA LAEL----- -
SPLC1_S208550_s CQYSNG---- ---------- ---------- -----RFGFS VQY------ -
SPLC1_S580170_s EQASRG---- ---------- ---------- -----RFGFG VQR------ -
SPLC1_S532860_s AQWRQVA--- --------SF PKLQRDLNPD PSAFKGLNLP VESV----- -
Consistency 2322321000 0000000000 0000000101 0210122223 442000000 0
660 670 680 690 700
PkA__PDBID_CHAI ---------- ---------- ---------- ---------- ---------- -
SPLC1_S032990_s -----AKDRI AKKEWDVVFT --------LS PIISVAAKDN SYTFAA--- -
SPLC1_S200190_s GPPGTGKTSV ITEIILQVLH KYPNDKILIS SQSNVAVDNV LIRLSRTPE K
SPLC1_S541370_s -----GRHDR ALVDYQKALE --------LD PDNSDVLTSK GTLLYQ--- -
SPLC1_S208550_s ---------- --RIWQEIGG S-----TSAD IETYKLFGEK VGW--R--- -
SPLC1_S580170_s ---------- --RIWEQV-- ---------- NQNTSEFAQR VGWCDR--- -
SPLC1_S532860_s ------AWYD VMEWCDRLSK R---IGKPYR LPSEAEWEYA ARA------ -
Consistency 0000001000 1132233511 0000000022 2233232222 211102000 0
710 720 730 740 750
PkA__PDBID_CHAI ---------- ---------- ---------- ---------- ---------- -
SPLC1_S032990_s --LMFPEGPP YYYSALYVRA DSPIQSINDI TPSTVIALGG FNSASSFYM P
SPLC1_S200190_s EIKCIRIGRE EKIEEDARKF EVEKAIIKWQ NFISAKSLAY WQNYQEENE Q
SPLC1_S541370_s ------TGEP Q---KALDAH EQAIAIDPNN ARAWHGKGIA LIGLQRYDE A
SPLC1_S208550_s ------RPND WLW---YNNL VFEIDKAPPG HLPSGRVGDI AIASRLMGK L
SPLC1_S580170_s ------HPSD DIYVKDYDQL SFSLK-APEG HLPALSVMAG --------R E
SPLC1_S532860_s ---------- ----GTTSPF HVGDTLTTDL ANYDGNHTYS SGPLGAYRG Q
Consistency 0000001212 1010213222 2223212321 2122222222 111121112 2
760 770 780 790 800
PkA__PDBID_CHAI ---------- ---------- ---------- ---------- ---------- -
SPLC1_S032990_s VYDLYGKTLT VDMGHRGQNI REMVRTGKAD LGA------- --------- G
SPLC1_S200190_s LLSGIQKIAQ LENVKTKNKE LQVLADKLTK IIARFNSELI VSQDNLASI D
SPLC1_S541370_s VSA-FEQAKT IR--PSAPSV WQSKALALEY QGKMAEA--- --------A Q
SPLC1_S208550_s GGFGLTRV-- -------LAI MAKLEECGIR ---------- --------- -
SPLC1_S580170_s WDRTLWLL-- -------EHL FLSVEACGL- ---------- --------- -
SPLC1_S532860_s TTPVGQFQN- ----ANAFGL YDIHGNVS-- ---------- --------- -
Consistency 2111222300 1000011124 2232322210 0000000000 000000000 0

```
                . . . . . . . . . 810 . . . . . . . . . 820 . . . . . . . . . 830 . . . . . . . . . 840 . . . . . . . . . 850
PkA__PDBID_CHAI ----------  ----------  ----------  ----------  ---------  -
SPLC1_S032990_s ALGDTVKNYP  DLRIIH--LS  RAIPGAGVYL  SPE-LSESDR  KVIERV---  -
SPLC1_S200190_s FADIAIELIS  EKLELEQKIL  KLVEQYTNNF  GVEYPGQKQL  SNWIEEEYK  V
SPLC1_S541370_s VYSEALATYD  DILREQPRRA  EIWVERGSVL  SKLGRHEQAL  ESYQKA---  -
SPLC1_S208550_s ----------  ----------  ----------  ----------  ---------  -
SPLC1_S580170_s ----------  ----------  ----------  ----------  ---------  -
SPLC1_S532860_s --EWCADPWH  DSYNGAPSDG  GVWDYDNDIR  YQKPIEYLVN  F--------  -
Consistency     0010111010  2010010001  1110001001  1010001101  000000000  0

                . . . . . . . . . 860 . . . . . . . . . 870 . . . . . . . . . 880 . . . . . . . . . 890 . . . . . . . . . 900
PkA__PDBID_CHAI ----------  ----------  ----------  ----------  ---------  -
SPLC1_S032990_s ----LLNAPP  DIQKQANYGL  GSEPDYTNFR  GIIRRTEEIL  VCSNFRQNP  V
SPLC1_S200190_s LHSILGDAQE  NYQKFINLQN  LNQEWNERLK  RKQQNLVPLF  IDEIDVVGA  T
SPLC1_S541370_s ----LEINPQ  HFQALLQKGN  VLFSPLGRTE  EAVRISDRAI  EVQPESHLA  W
SPLC1_S208550_s ----------  ----------  ----------  ----------  ---------  -
SPLC1_S580170_s ----------  ----------  ----------  ----------  ---------  -
SPLC1_S532860_s --------LE  SQERRVLRGG  SWLFNPVVCR  CANRDRG---  -----GSDL  L
Consistency     0000100001  1021011010  0000000101  0102010000  000000001  0

                . . . . . . . . . 910 . . . . . . . . . 920 . . . . . . . . . 930 . . . . . . . . . 940 . . . . . . . . . 950
PkA__PDBID_CHAI ----------  ----------  ----------  ----------  ---------  -
SPLC1_S032990_s NFFCGTGVGT  VPTTVNHGGN  EIRGRVNGWR  R---------  --------P  T
SPLC1_S200190_s CLGVARFKER  NFDWVIIDEA  GRSTASETFV  PMSKGKKIIL  VGDHRQLPP  I
SPLC1_S541370_s -HNRGSILAG  GRGDF----E  GAIAAYDRAI  ELR-------  --------P  S
SPLC1_S208550_s ----------  ----------  ----------  ----------  ---------  -
SPLC1_S580170_s ----------  ----------  ----------  ----------  ---------  -
SPLC1_S532860_s YFSCGFRVAC  AL--------  ----------  ----------  ---------  -
Consistency     0100200110  0000000000  0000000000  0000000000  000000001  0

                . . . . . . . . . 960 . . . . . . . . . 970 . . . . . . . . . 980 . . . . . . . . . 990 . . . . . . . . . 1000
PkA__PDBID_CHAI ----------  ----------  ----------  ----------  ---------  -
SPLC1_S032990_s V--DTVWLN-  ---------L  MAEGNQL---  ----------  ----YRVVV  S
SPLC1_S200190_s IEQELQERAF  SEKEIHKRLL  ETSLFEYLYD  KLPAHNKITL  NNQYRMHPN  I
SPLC1_S541370_s F--VPALRD-  ---------R  GFALSQWSQA  L------RAE  GNTSMANAK  I
SPLC1_S208550_s ----------  ----------  ----------  ----------  ---------  -
SPLC1_S580170_s ----------  ----------  ----------  ----------  ---------  -
SPLC1_S532860_s ----------  ----------  ----------  ----------  ---------  -
Consistency     0000000000  0000000000  0000010000  0000000000  000000000  0

                . . . . . . . . . 1010 . . . . . . . . . 1020 . . . . . . . . . 1030 . . . . . . . . . 1040 . . . . . . . . . 1050
PkA__PDBID_CHAI ----------  ----------  ----------  ----------  ---------  -
SPLC1_S032990_s SQ--ILNQVP  GA--------  ----------  ----------  ---------  -
SPLC1_S200190_s GNLVSALFYD  NQVASESVNI  PEKQHSLTIF  EQSVYWISTS  DEPETEKKE  R
SPLC1_S541370_s NE--ALESF-  ----------  ----------  ----------  ---------  -
SPLC1_S208550_s ----------  ----------  ----------  ----------  ---------  -
SPLC1_S580170_s ----------  ----------  ----------  ----------  ---------  -
SPLC1_S532860_s ----------  ----------  ----------  ----------  ---------  -
Consistency     0000000000  0000000000  0000000000  0000000000  000000000  0

                . . . . . . . . . 1060 . . . . . . . . . 1070 . . . . . . . . . 1080 . . . . . . . . . 1090 . . . . . . . . . 1100
PkA__PDBID_CHAI ----------  ----------  ----------  ----------  ---------  -
SPLC1_S032990_s --------TN  LLELQNKEIK  VM------GV  VPNQIG----  -DGILELNI  Q
SPLC1_S200190_s QNGKSRSNPY  EAKVIKEVLS  KIQENCELNN  LHKEVGVIAA  YRSQISIL-  E
SPLC1_S541370_s --------DR  ALNINPNDHQ  SLVGR---AI  AFSHQG---R  YDESLNAF-  D
SPLC1_S208550_s ----------  ----------  ----------  ----------  ---------  -
SPLC1_S580170_s ----------  ----------  ----------  ----------  ---------  -
SPLC1_S532860_s ----------  ----------  ----------  ----------  ---------  -
Consistency     0000000000  0001000000  0000000000  0000010000  000010000  0
```

```
                 .........1110.........1120.........1130.........1140.........1150
PkA__PDBID_CHAI ---------- ---------- ---------- ---------- --------- -
SPLC1_S032990_s NSMELEVL-- ---------- ---------- ---------- --------- -
SPLC1_S200190_s SAIAPNDQQF WQNLHIIIHT VDAFQGGECD IIIYDLVRSN RDNKLGFTS D
SPLC1_S541370_s KAQEIQ---- ---------- -PQDP----- ---------- --------- -
SPLC1_S208550_s ---------- ---------- ---------- ---------- --------- -
SPLC1_S580170_s ---------- ---------- ---------- ---------- --------- -
SPLC1_S532860_s ---------- ---------- ---------- ---------- --------- -
Consistency     0100000000 0000000000 0000000000 0000000000 000000000 0

                 .........1160.........1170.........1180.........1190.........1200
PkA__PDBID_CHAI ---------- ---------- ---------- ---------- --------- -
SPLC1_S032990_s ---------- ---------- ---------- ---------- --------- -
SPLC1_S200190_s DRRLNVALSR AKQLLIIVGD DNMAYQGRTP RNITNPFKP- LIEYIDSNN N
SPLC1_S541370_s LILVNRGLVL ERMGRY---- NEAI---DAY DEALKIQPGF PPAVRS--- -
SPLC1_S208550_s ---------- ---------- ---------- ---------- --------- -
SPLC1_S580170_s ---------- ---------- ---------- ---------- --------- -
SPLC1_S532860_s ---------- ---------- ---------- ---------- --------- -
Consistency     0000000000 0000000000 0000000000 0000000000 000000000 0

                 .........1210.
PkA__PDBID_CHAI ---------- -
SPLC1_S032990_s ---------- -
SPLC1_S200190_s SCCRLISSKF S
SPLC1_S541370_s --KNQLQQRL -
SPLC1_S208550_s ---------- -
SPLC1_S580170_s ---------- -
SPLC1_S532860_s ---------- -
Consistency     0000000000 0
```
