# Supplementary material for: Revealing the key point of the temperature stress response of Arthrospira platensis C1 at the interconnection of C- and N- metabolism by proteome analyses and PPI networking
Source: BMC Mol Cell Biol. 2020 Jun 12;21:43. doi: 10.1186/s12860-020-00285-y (PMC7291507; doi:10.1186/s12860-020-00285-y)
Supplement: Supplementary file 6 — Additional file 6. PPI subnetwork of Hik28. The subnetwork was constructed by using STRING. The A. platensis C1 proteins were inferred to that of the A. platensis NIES39 via orthologous group. [file 12860_2020_285_MOESM6_ESM.docx]

**Additional file 6**


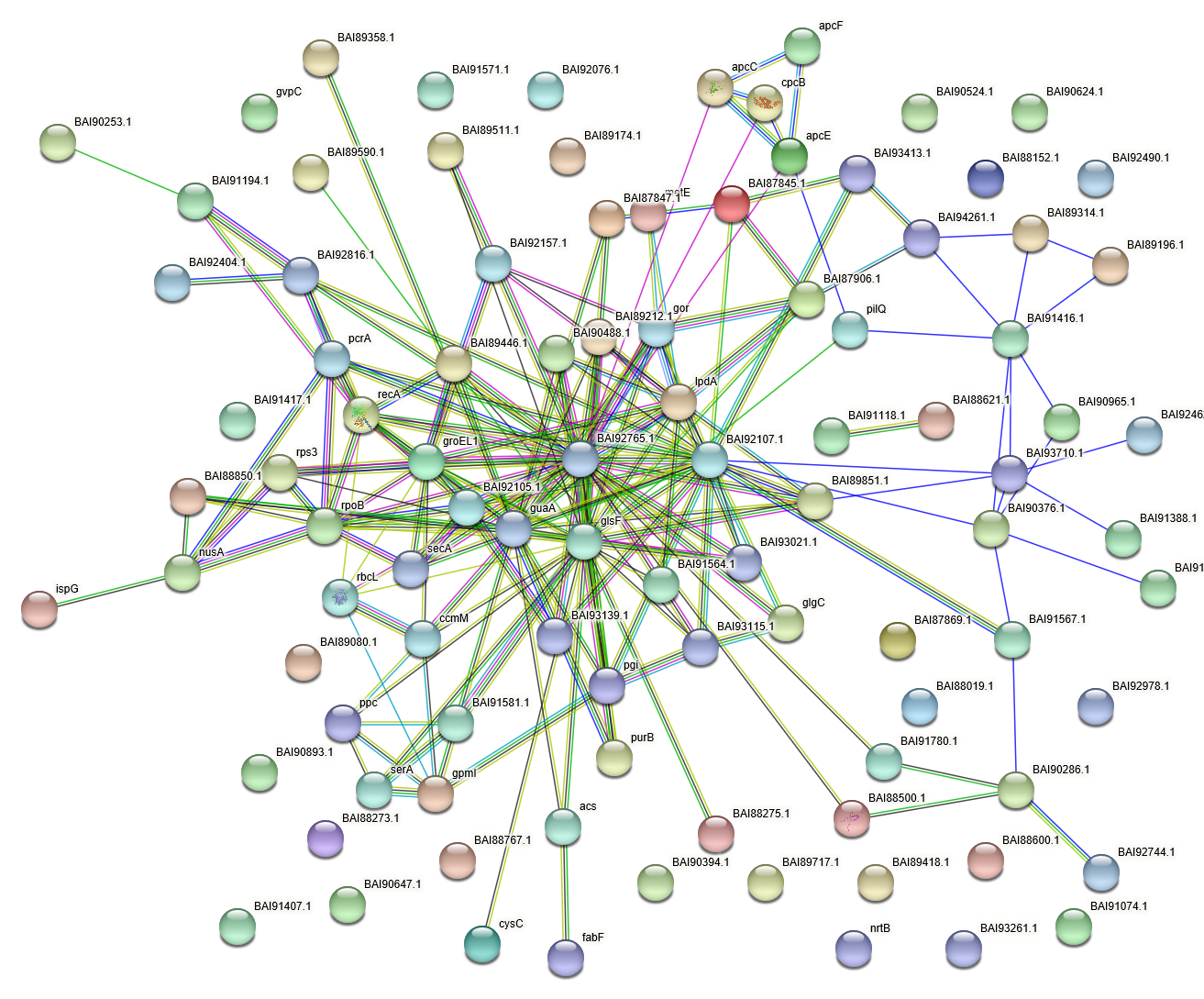


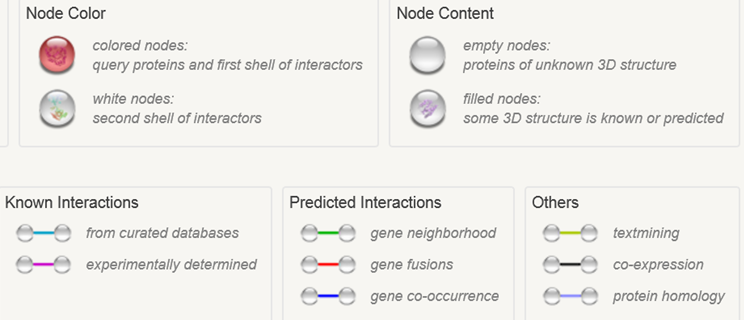


| **node** | **identifier** | **annotation** |
| --- | --- | --- |
| BAI87845.1 | NIES39_A00040 | Hypothetical protein |
| BAI87847.1 | NIES39_A00060 | Hypothetical protein |
| BAI87869.1 | NIES39_A00280 | TPR domain protein |
| BAI87906.1 | NIES39_A00650 | Putative short-chain dehydrogenase |
| apcE | NIES39_A00830 | Phycobilisome core-membrane linker polypeptide |
| groEL1 | NIES39_A01110 | Chaperonin GroEL; Prevents misfolding and promotes the refolding and proper assembly of unfolded polypeptides generated under stress conditions |
| cysC | NIES39_A01770 | Adenylylsulfate kinase; Catalyzes the synthesis of activated sulfate |
| BAI88019.1 | NIES39_A01800 | UDP-glucose:tetrahydrobiopterin glucosyltransferase |
| BAI88152.1 | NIES39_A03130 | Hypothetical protein |
| BAI88273.1 | NIES39_A04350 | Competence protein ComM homolog |
| BAI88275.1 | NIES39_A04370 | Putative permease |
| BAI88455.1 | NIES39_A06170 | Two-component sensor histidine kinase |
| BAI88500.1 | NIES39_A06620 | Type I restriction-modification system R subunit |
| metE | NIES39_A07560 | 5-methyltetrahydropteroyltriglutamate--homocysteine S-methyltransferase; Catalyzes the transfer of a methyl group from 5- methyltetrahydrofolate to homocysteine resulting in methionine formation |
| BAI88600.1 | NIES39_A07620 | Hypothetical protein |
| ispG | NIES39_A07760 | 4-hydroxy-3-methylbut-2-en-1-yl diphosphate synthase; Converts 2C-methyl-D-erythritol 2,4-cyclodiphosphate (ME-2,4cPP) into 1-hydroxy-2-methyl-2-(E)-butenyl 4-diphosphate |
| BAI88621.1 | NIES39_A07830 | L-asparaginase |
| BAI88767.1 | NIES39_B00100 | Hypothetical protein |
| BAI88780.1 | NIES39_B00230 | polyA polymerase |
| BAI88850.1 | NIES39_B00930 | GTP-binding protein TypA homolog |
| gpmI | NIES39_B01040 | 2,3-bisphosphoglycerate-independent phosphoglycerate mutase; Catalyzes the interconversion of 2-phosphoglycerate and 3-phosphoglycerate |
| BAI89080.1 | NIES39_C02120 | Hypothetical protein |
| BAI89174.1 | NIES39_C03060 | Hypothetical protein |
| BAI89196.1 | NIES39_C03290 | Hypothetical protein |
| lpdA | NIES39_C03380 | Dihydrolipoamide dehydrogenase |
| BAI89212.1 | NIES39_C03450 | Two-component hybrid sensor and regulator |
| BAI89314.1 | NIES39_C04480 | Hypothetical protein |
| apcC | NIES39_C04520 | Phycobilisome small core linker polypeptide; Rod linker protein, associated with allophycocyanin. Linker polypeptides determine the state of aggregation and the location of the disk-shaped phycobiliprotein units within the phycobilisome and modulate their spectroscopic properties in order to mediate a directed and optimal energy transfer |
| BAI89358.1 | NIES39_C04920 | Putative cytosine deaminase |
| BAI89418.1 | NIES39_C05520 | Hypothetical protein |
| BAI89446.1 | NIES39_D00260 | ATP-dependent Clp protease ATP-binding subunit ClpC |
| BAI89511.1 | NIES39_D00910 | CRP/FNR family transcriptional regulator |
| BAI89590.1 | NIES39_D01700 | Serine/threonine protein kinase |
| cpcB | NIES39_D02060 | C-phycocyanin beta subunit |
| BAI89717.1 | NIES39_D02970 | Hypothetical protein |
| purB | NIES39_D02980 | Adenylosuccinate lyase |
| recA | NIES39_D04210 | Recombination protein RecA; Can catalyze the hydrolysis of ATP in the presence of single-stranded DNA, the ATP-dependent uptake of single-stranded DNA by duplex DNA, and the ATP-dependent hybridization of homologous single-stranded DNAs. It interacts with LexA causing its activation and leading to its autocatalytic cleavage |
| BAI89851.1 | NIES39_D04330 | Two-component hybrid sensor and regulator |
| rpl2 | NIES39_D06420 | 50S ribosomal protein L2; One of the primary rRNA binding proteins. Required for association of the 30S and 50S subunits to form the 70S ribosome, for tRNA binding and peptide bond formation. It has been suggested to have peptidyltransferase activity; this is somewhat controversial. Makes several contacts with the 16S rRNA in the 70S ribosome |
| rplV | NIES39_D06440 | 50S ribosomal protein L22; The globular domain of the protein is located near the polypeptide exit tunnel on the outside of the subunit, while an extended beta-hairpin is found that lines the wall of the exit tunnel in the center of the 70S ribosome |
| rps3 | NIES39_D06450 | 30S ribosomal protein S3; Binds the lower part of the 30S subunit head. Binds mRNA in the 70S ribosome, positioning it for translation |
| glgC | NIES39_D06800 | Glucose-1-phosphate adenylyltransferase; Catalyzes the synthesis of ADP-glucose, a sugar donor used in elongation reactions on alpha-glucans |
| BAI90253.1 | NIES39_E00180 | Hypothetical protein |
| BAI90286.1 | NIES39_E00520 | Hypothetical protein |
| BAI90376.1 | NIES39_E01430 | Peptidoglycan-binding domain 1 protein |
| BAI90394.1 | NIES39_E01620 | Putative TRAP dicarboxylate transporter DctP subunit; Part of the tripartite ATP-independent periplasmic (TRAP) transport system |
| nusA | NIES39_E01730 | Transcription termination factor NusA; Participates in both transcription termination and antitermination |
| BAI90488.1 | NIES39_E02610 | Two-component hybrid histidine kinase |
| BAI90524.1 | NIES39_E02970 | Two-component hybrid histidine kinase |
| BAI90542.1 | NIES39_E03150 | Hypothetical protein |
| rpoB | NIES39_E03620 | RNA polymerase beta subunit; DNA-dependent RNA polymerase catalyzes the transcription of DNA into RNA using the four ribonucleoside triphosphates as substrates |
| rpoC1 | NIES39_E03630 | RNA polymerase gamma subunit; DNA-dependent RNA polymerase catalyzes the transcription of DNA into RNA using the four ribonucleoside triphosphates as substrates |
| rpoC2 | NIES39_E03640 | RNA polymerase beta prime subunit; DNA-dependent RNA polymerase catalyzes the transcription of DNA into RNA using the four ribonucleoside triphosphates as substrates |
| BAI90624.1 | NIES39_E03970 | HlyD family secretion protein |
| BAI90647.1 | NIES39_E04200 | Hypothetical protein |
| guaB | NIES39_G00770 | Inositol-5-monophosphate dehydrogenase |
| BAI90893.1 | NIES39_G01100 | Glutathione S-transferase C-terminal domain protein |
| BAI90965.1 | NIES39_H00400 | Probable glycosyl transferase |
| gvpC | NIES39_H01070 | Gas vesicle protein GvpC |
| BAI91074.1 | NIES39_J00220 | Pentapeptide repeat-containing protein |
| apcF | NIES39_J00490 | Allophycocyanin beta-18 subunit |
| BAI91118.1 | NIES39_J00660 | ABC transporter ATP-binding protein |
| BAI91125.1 | NIES39_J00730 | Hypothetical protein |
| BAI91194.1 | NIES39_J01420 | single-stranded-DNA-specific exonuclease |
| BAI91388.1 | NIES39_J03410 | Hypothetical protein |
| BAI91407.1 | NIES39_J03600 | TM1812 family CRISPR-associated protein |
| BAI91416.1 | NIES39_J03690 | Hypothetical protein |
| BAI91417.1 | NIES39_J03700 | Pentapeptide repeat-containing protein |
| BAI91564.1 | NIES39_J05180 | DNA-binding protein HU |
| BAI91567.1 | NIES39_J05210 | Hypothetical protein |
| BAI91571.1 | NIES39_J05250 | TPR domain protein |
| BAI91581.1 | NIES39_J05350 | Aminotransferase; Involved in the synthesis of meso-diaminopimelate (m-DAP or DL-DAP), required for both lysine and peptidoglycan biosynthesis. Catalyzes the direct conversion of tetrahydrodipicolinate to LL-diaminopimelate, a reaction that requires three enzymes in E.coli |
| glsF | NIES39_J05540 | Ferredoxin-dependent glutamate synthase |
| acs | NIES39_J05970 | Acetyl-coenzyme A synthetase; Catalyzes the conversion of acetate into acetyl-CoA (AcCoA), an essential intermediate at the junction of anabolic and catabolic pathways. AcsA undergoes a two-step reaction. In the first half reaction, AcsA combines acetate with ATP to form acetyl-adenylate (AcAMP) intermediate. In the second half reaction, it can then transfer the acetyl group from AcAMP to the sulfhydryl group of CoA, forming the product AcCoA |
| BAI91780.1 | NIES39_K01310 | Type I restriction-modification system endonuclease, fragment |
| serA | NIES39_K02580 | D-3-phosphoglycerate dehydrogenase |
| rbcL | NIES39_K02850 | Ribulose-1,5-bisphosphate carboxylase/oxygenase large subunit |
| pilQ | NIES39_K03570 | Bifunctional pilus assembly protein PilQ/type II secretion pathway protein D |
| BAI92076.1 | NIES39_K04310 | Hypothetical protein |
| BAI92105.1 | NIES39_K04600 | Cell division protein FtsH; Acts as a processive, ATP-dependent zinc metallopeptidase for both cytoplasmic and membrane proteins. Plays a role in the quality control of integral membrane proteins |
| BAI92107.1 | NIES39_K04620 | Probable glycosyl transferase |
| ccmM | NIES39_K04800 | Carbon dioxide concentrating mechanism protein CcmM |
| BAI92157.1 | NIES39_K05120 | Peptidase, M16 family |
| gor | NIES39_L00870 | Glutathione reductase |
| pcrA | NIES39_L02420 | ATP-dependent DNA helicase PcrA |
| BAI92404.1 | NIES39_L02440 | Probable transglycosylase |
| BAI92462.1 | NIES39_L03050 | Hypothetical protein |
| BAI92490.1 | NIES39_L03330 | Hypothetical protein |
| BAI92744.1 | NIES39_L05870 | Hypothetical protein |
| BAI92765.1 | NIES39_L06080 | Thioredoxin reductase |
| guaA | NIES39_L06160 | GMP synthase (glutamine-hydrolyzing); Catalyzes the synthesis of GMP from XMP |
| BAI92816.1 | NIES39_L06590 | Hypothetical protein |
| secA | NIES39_M00550 | Preprotein translocase SecA subunit; Part of the Sec protein translocase complex. Interacts with the SecYEG preprotein conducting channel. Has a central role in coupling the hydrolysis of ATP to the transfer of proteins into and across the cell membrane, serving as an ATP-driven molecular motor driving the stepwise translocation of polypeptide chains across the membrane |
| BAI92978.1 | NIES39_M01410 | Hypothetical protein |
| BAI93021.1 | NIES39_M01840 | Hypothetical protein (Orthologous of SPLC1_S540320: Diguanylate cyclase (PleD) in *A. platensis* C1) |
| BAI93115.1 | NIES39_M02780 | Phosphoglucomutase |
| BAI93139.1 | NIES39_N00220 | ABC transporter ATP-binding protein |
| BAI93147.1 | NIES39_N00300 | Hypothetical protein |
| BAI93261.1 | NIES39_O00100 | Hypothetical protein |
| nrtB | NIES39_O00860 | Nitrate ABC-type transport permease protein |
| BAI93413.1 | NIES39_O01640 | Putative UDP-glucuronic acid decarboxylase |
| BAI93710.1 | NIES39_O04630 | FHA domain containing protein |
| rps4 | NIES39_O05200 | 30S ribosomal protein S4; One of the primary rRNA binding proteins, it binds directly to 16S rRNA where it nucleates assembly of the body of the 30S subunit |
| pgi | NIES39_Q00590 | Glucose-6-phosphate isomerase |
| fabF | NIES39_Q02320 | 3-oxoacyl-[acyl-carrier-protein] synthase II; Catalyzes the condensation reaction of fatty acid synthesis by the addition to an acyl acceptor of two carbons from malonyl-ACP |
| BAI94261.1 | NIES39_Q02530 | Probable oxidoreductase |
| ppc | NIES39_Q02830 | Phosphoenolpyruvate carboxylase; Forms oxaloacetate, a four-carbon dicarboxylic acid source for the tricarboxylic acid cycle |
