# Supplementary material for: Revealing the key point of the temperature stress response of Arthrospira platensis C1 at the interconnection of C- and N- metabolism by proteome analyses and PPI networking
Source: BMC Mol Cell Biol. 2020 Jun 12;21:43. doi: 10.1186/s12860-020-00285-y (PMC7291507; doi:10.1186/s12860-020-00285-y)
Supplement: Supplementary file 7 — Additional file 7. PPI subnetwork of Hik28 and enzymes involved in fatty acid desaturation. The subnetwork was constructed by using STRING. The A. platensis C1 proteins were inferred to that of the Synechocystis sp. PCC6803 via orthologous group. [file 12860_2020_285_MOESM7_ESM.docx]

**Additional file 7**

**
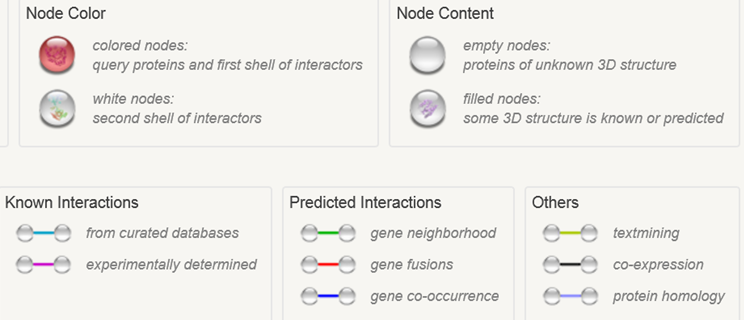
**

**
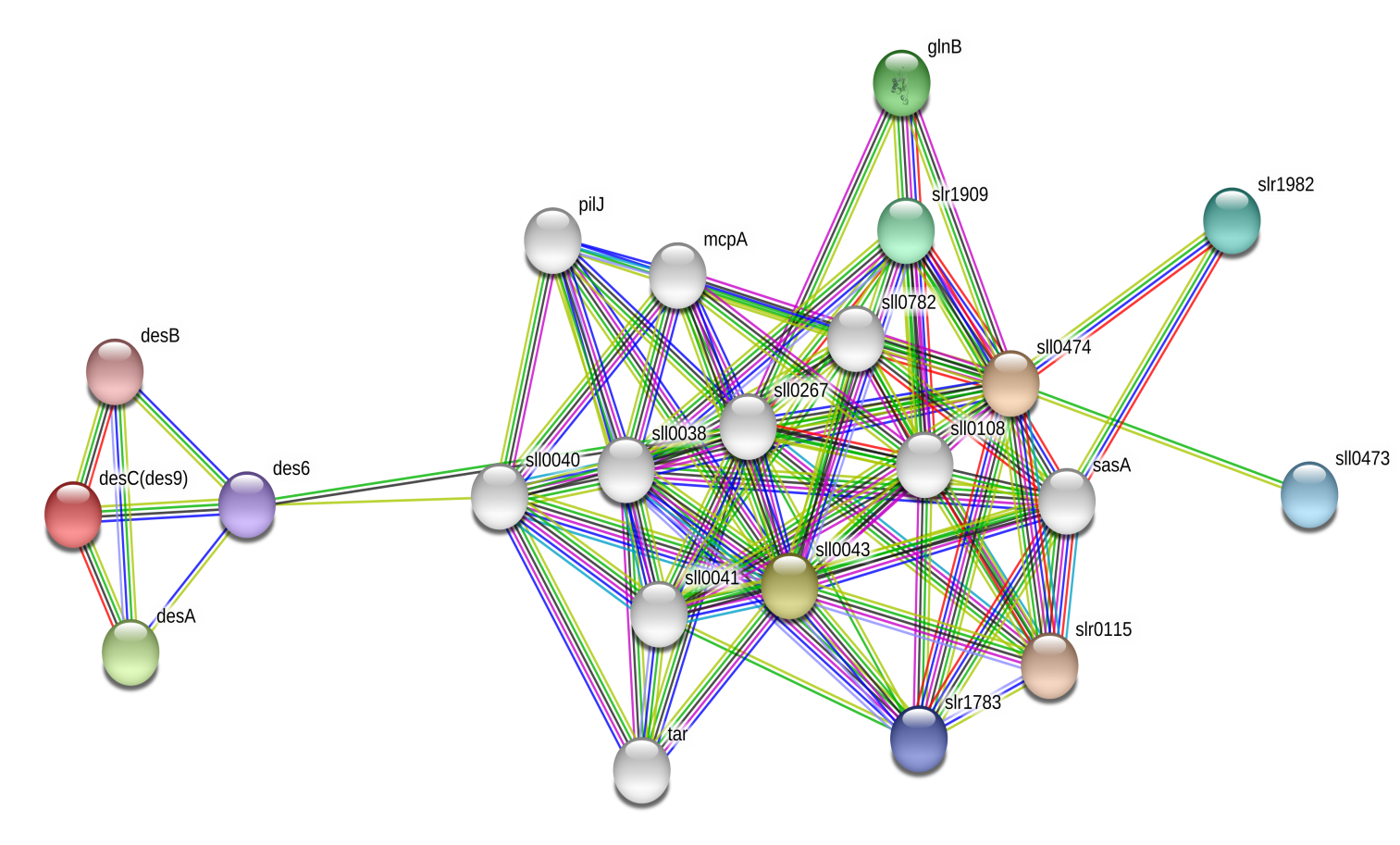
**

| **node** | **identifier** | **annotation** |
| --- | --- | --- |
| slr1783 | SYNGTS_0220 | NarL subfamily protein Rre1 |
| mcpA | SYNGTS_0452 | Methyl-accepting chemotaxis protein |
| slr1909 | SYNGTS_0555 | NarL subfamily protein Rre9 |
| pilJ | SYNGTS_0624 | Methyl-accepting chemotaxis protein(MCP) homolog |
| desA | SYNGTS_1594 | Fatty acid desaturase |
| slr1982 | SYNGTS_1649 | CheY subfamily protein Rre21 |
| desB | SYNGTS_1727 | Delta 15 desaturase |
| sll0267 | SYNGTS_1926 | Hypothetical protein |
| des6 | SYNGTS_1931 | Delta-6 desaturase |
| glnB | SYNGTS_1962 | Nitrogen regulatory protein P-II |
| sasA | SYNGTS_2178 | Sensory transduction histidine kinase SasA/Hik8 |
| desC(des9) | SYNGTS_2538 | Delta-9 desaturase |
| sll0474 | SYNGTS_2632 | Sensory transduction histidine kinase Hik28 |
| sll0473 | SYNGTS_2633 | Hypothetical protein |
| sll0108 | SYNGTS_2669 | Ammonium/methylammonium permease |
| slr0115 | SYNGTS_2690 | OmpR subfamily protein RpaA/Ycf27/Crr31/Rre31 |
| sll0782 | SYNGTS_2757 | Putative protein kinase |
| sll0043 | SYNGTS_2822 | CheA like protein Hik18 |
| tar | SYNGTS_2823 | Methyl-accepting chemotaxis protein II |
| sll0041 | SYNGTS_2824 | Phytochrome-like photoreceptor protein for positive phototaxis |
| sll0040 | SYNGTS_2825 | Hypothetical protein |
| sll0038 | SYNGTS_2827 | PatA subfamily protein PixG/PisG/TaxP1/Rer1/Rre36 |
