# Supplementary material for: Revealing the key point of the temperature stress response of Arthrospira platensis C1 at the interconnection of C- and N- metabolism by proteome analyses and PPI networking
Source: BMC Mol Cell Biol. 2020 Jun 12;21:43. doi: 10.1186/s12860-020-00285-y (PMC7291507; doi:10.1186/s12860-020-00285-y)
Supplement: Supplementary file 8 — Additional file 8. PPI subnetwork of the bi-level regulated proteins. The subnetwork was constructed by using STRING. The A. platensis C1 proteins were inferred to that of the A. platensis NIES39 via orthologous group. [file 12860_2020_285_MOESM8_ESM.docx]

**Additional file 8**

­
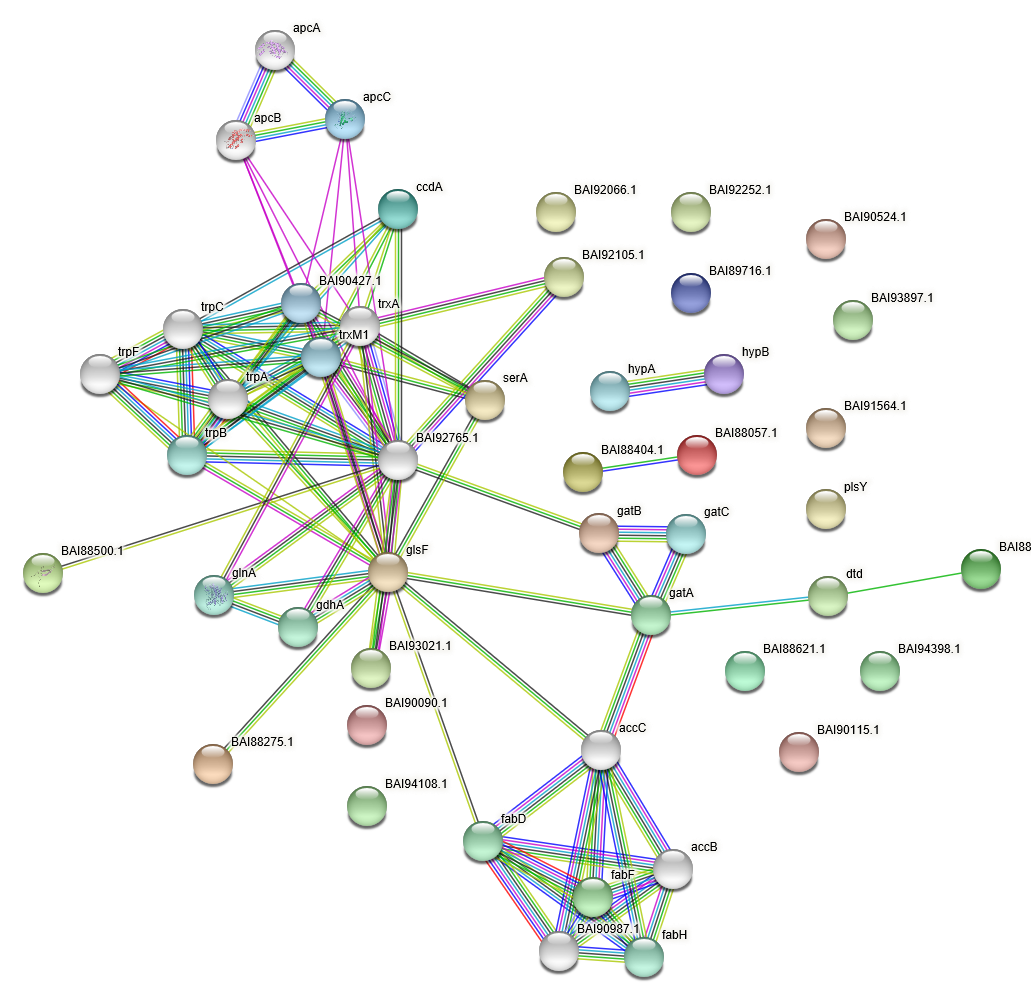


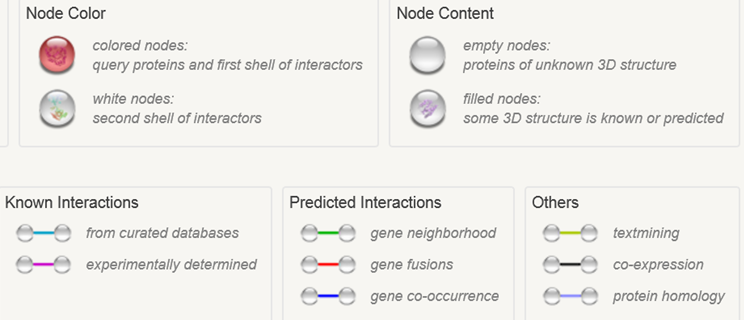


| **node** | **identifier** | **annotation** |
| --- | --- | --- |
| BAI88057.1 | NIES39_A02180 | Hypothetical protein |
| BAI88275.1 | NIES39_A04370 | Putative permease |
| BAI88404.1 | NIES39_A05660 | Hypothetical protein |
| BAI88500.1 | NIES39_A06620 | Type I restriction-modification system R subunit |
| BAI88564.1 | NIES39_A07260 | Hypothetical protein; Specifically methylates the N3 position of the uracil ring of uridine 1498 (m3U1498) in 16S rRNA. Acts on the fully assembled 30S ribosomal subunit |
| BAI88621.1 | NIES39_A07830 | L-asparaginase |
| ccdA | NIES39_A07940 | Putative c-type cytochrome biogenesis protein CcdA |
| apcC | NIES39_C04520 | Phycobilisome small core linker polypeptide; Rod linker protein, associated with allophycocyanin. Linker polypeptides determine the state of aggregation and the location of the disk-shaped phycobiliprotein units within the phycobilisome and modulate their spectroscopic properties in order to mediate a directed and optimal energy transfer |
| apcB | NIES39_C04530 | Allophycocyanin beta subunit |
| apcA | NIES39_C04540 | Allophycocyanin alpha subunit |
| BAI89716.1 | NIES39_D02960 | Putative endodeoxyribonuclease |
| hypB | NIES39_D03400 | Hydrogenase accessory protein HypB |
| hypA | NIES39_D03410 | Hydrogenase nickel insertion protein HypA; Probably plays a role in a hydrogenase nickel cofactor insertion step |
| BAI90090.1 | NIES39_D06730 | ABC transporter ATP-binding protein |
| BAI90115.1 | NIES39_D06980 | Hypothetical protein |
| trpC | NIES39_D07270 | Indole-3-glycerol phosphate synthase |
| gatA | NIES39_E01380 | glutamyl-tRNA(Gln) amidotransferase subunit A; Allows the formation of correctly charged Gln-tRNA(Gln) through the transamidation of misacylated Glu-tRNA(Gln) in organisms which lack glutaminyl-tRNA synthetase. The reaction takes place in the presence of glutamine and ATP through an activated gamma-phospho-Glu-tRNA(Gln) |
| gdhA | NIES39_E01630 | Glutamate dehydrogenase |
| BAI90427.1 | NIES39_E01990 | Thioredoxin |
| BAI90524.1 | NIES39_E02970 | Two-component hybrid histidine kinase |
| trpF | NIES39_E02990 | Phosphoribosylanthranilate isomerase |
| gatB | NIES39_E03060 | glutamyl-tRNA(Gln) amidotransferase subunit B; Allows the formation of correctly charged Asn-tRNA(Asn) or Gln-tRNA(Gln) through the transamidation of misacylated Asp- tRNA(Asn) or Glu-tRNA(Gln) in organisms which lack either or both of asparaginyl-tRNA or glutaminyl-tRNA synthetases. The reaction takes place in the presence of glutamine and ATP through an activated phospho-Asp-tRNA(Asn) or phospho-Glu-tRNA(Gln) |
| trpA | NIES39_F00460 | Tryptophan synthase alpha chain; The alpha subunit is responsible for the aldol cleavage of indoleglycerol phosphate to indole and glyceraldehyde 3- phosphate |
| trxA | NIES39_G00780 | Thioredoxin |
| BAI90987.1 | NIES39_H00620 | 3-oxoacyl-[acyl-carrier-protein] synthase |
| glnA | NIES39_J00500 | Glutamate--ammonia ligase, glutamine synthetase type I |
| BAI91564.1 | NIES39_J05180 | DNA-binding protein HU |
| glsF | NIES39_J05540 | Ferredoxin-dependent glutamate synthase |
| serA | NIES39_K02580 | D-3-phosphoglycerate dehydrogenase |
| plsY | NIES39_K03280 | Hypothetical protein; Catalyzes the transfer of an acyl group from acyl- phosphate (acyl-PO(4)) to glycerol-3-phosphate (G3P) to form lysophosphatidic acid (LPA). This enzyme utilizes acyl-phosphate as fatty acyl donor, but not acyl-CoA or acyl-ACP |
| BAI92066.1 | NIES39_K04210 | Adenylate cyclase |
| BAI92105.1 | NIES39_K04600 | Cell division protein FtsH; Acts as a processive, ATP-dependent zinc metallopeptidase for both cytoplasmic and membrane proteins. Plays a role in the quality control of integral membrane proteins |
| BAI92252.1 | NIES39_L00910 | Hypothetical protein |
| gatC | NIES39_L04400 | glutamyl-tRNA(Gln) amidotransferase subunit C; Allows the formation of correctly charged Asn-tRNA(Asn) or Gln-tRNA(Gln) through the transamidation of misacylated Asp- tRNA(Asn) or Glu-tRNA(Gln) in organisms which lack either or both of asparaginyl-tRNA or glutaminyl-tRNA synthetases. The reaction takes place in the presence of glutamine and ATP through an activated phospho-Asp-tRNA(Asn) or phospho-Glu-tRNA(Gln) |
| BAI92765.1 | NIES39_L06080 | Thioredoxin reductase |
| BAI93021.1 | NIES39_M01840 | Hypothetical protein |
| trxM1 | NIES39_N00250 | Thioredoxin M |
| fabH | NIES39_N01190 | 3-oxoacyl-[acyl-carrier-protein] synthase III; Catalyzes the condensation reaction of fatty acid synthesis by the addition to an acyl acceptor of two carbons from malonyl-ACP. Catalyzes the first condensation reaction which initiates fatty acid synthesis and may therefore play a role in governing the total rate of fatty acid production. Possesses both acetoacetyl-ACP synthase and acetyl transacylase activities. Its substrate specificity determines the biosynthesis of branched- chain and/or straight-chain of fatty acids |
| fabD | NIES39_N01200 | Malonyl coenzyme A-acyl carrier protein transacylase |
| dtd | NIES39_O01770 | D-tyrosyl-tRNA(Tyr) deacylase; D-aminoacyl-tRNA deacylase with broad substrate specificity. By recycling D-aminoacyl-tRNA to D-amino acids and free tRNA molecules, this enzyme counteracts the toxicity associated with the formation of D-aminoacyl-tRNA entities in vivo |
| accC | NIES39_O03320 | acetyl-CoA carboxylase, biotin carboxylase subunit |
| accB | NIES39_O04490 | Biotin carboxyl carrier protein |
| trpB | NIES39_O06170 | Tryptophan synthase beta chain; The beta subunit is responsible for the synthesis of L- tryptophan from indole and L-serine |
| BAI93897.1 | NIES39_O06510 | Transglutaminase-like domain |
| BAI94108.1 | NIES39_Q01000 | Adenylate cyclase |
| fabF | NIES39_Q02320 | 3-oxoacyl-[acyl-carrier-protein] synthase II; Catalyzes the condensation reaction of fatty acid synthesis by the addition to an acyl acceptor of two carbons from malonyl-ACP |
| BAI94398.1 | NIES39_R00890 | Hypothetical protein |
