# Supplementary figures and images for: Revealing the key point of the temperature stress response of Arthrospira platensis C1 at the interconnection of C- and N- metabolism by proteome analyses and PPI networking
Source: BMC Mol Cell Biol. 2020 Jun 12;21:43. doi: 10.1186/s12860-020-00285-y (PMC7291507; doi:10.1186/s12860-020-00285-y)

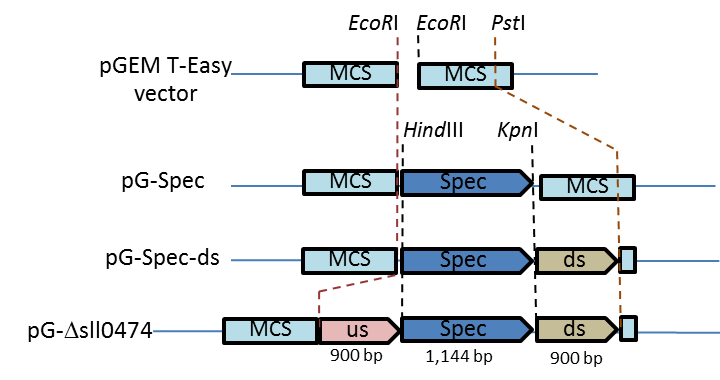

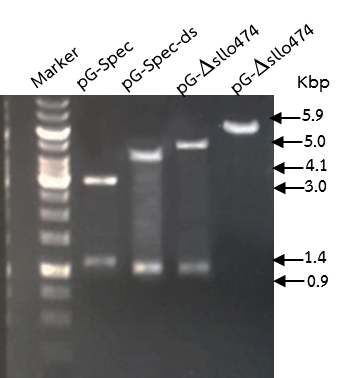


A

B

C

D


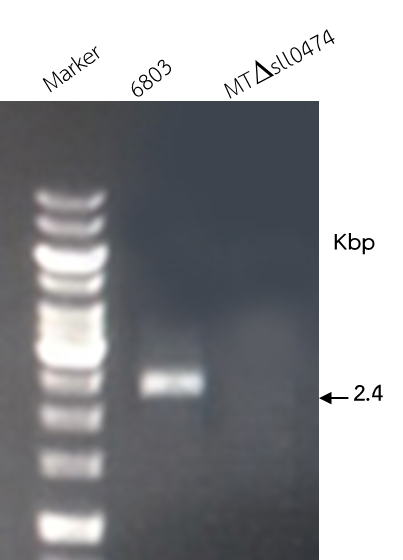

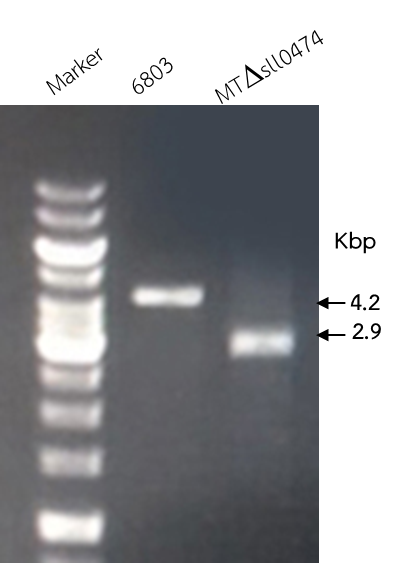

Supplement: Supplementary file 10 — Additional file 10. Construction of Δsll0474 mutant in Synechocystis sp. PCC6803; (A) Map of the recombinant plasmid containing the upstream (us) and the downstream (ds) regions of the sll0474 gene, and the spectinomycin resistant gene (Spec) cloned in pGEM T-Easy vector (B) Enzymatic digestion of the recombinant plasmids as shown in (A) represented the inserts (The recombinant plasmids in the last two lanes of the agarose gel were digested with EcoRI&HindIII and PstI, respectively.) (C) PCR products of Synechocystis sp. 6803WT and Δsll0474 mutant amplified by using US-sll0474ERIFW and DS-sll0474pstIRVprimers (D) PCR products of Synechocystis sp. 6803WT and Δsll0474 mutant amplified by using Sll0474ERIFW and Sll0474BaHIRV primers. [file 12860_2020_285_MOESM10_ESM.docx]

**Additional file 11**


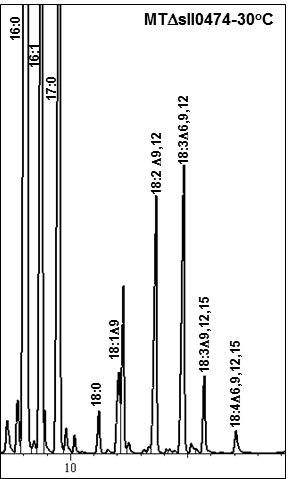

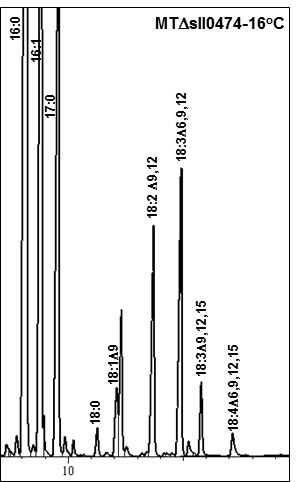

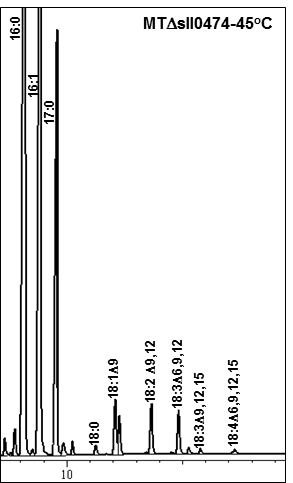

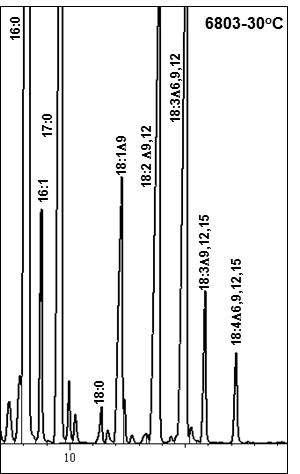

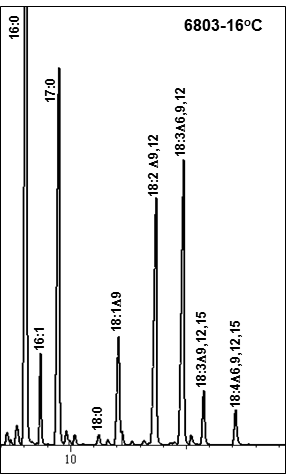

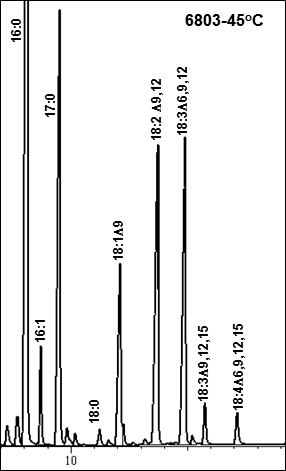

Supplement: Supplementary file 11 — Additional file 11. Fatty acid analysis profile obtained by using gas chromatography of Synechocystis sp. PCC6803 WT and Δsll0474 mutant. [file 12860_2020_285_MOESM11_ESM.docx]

**Additional File 17**


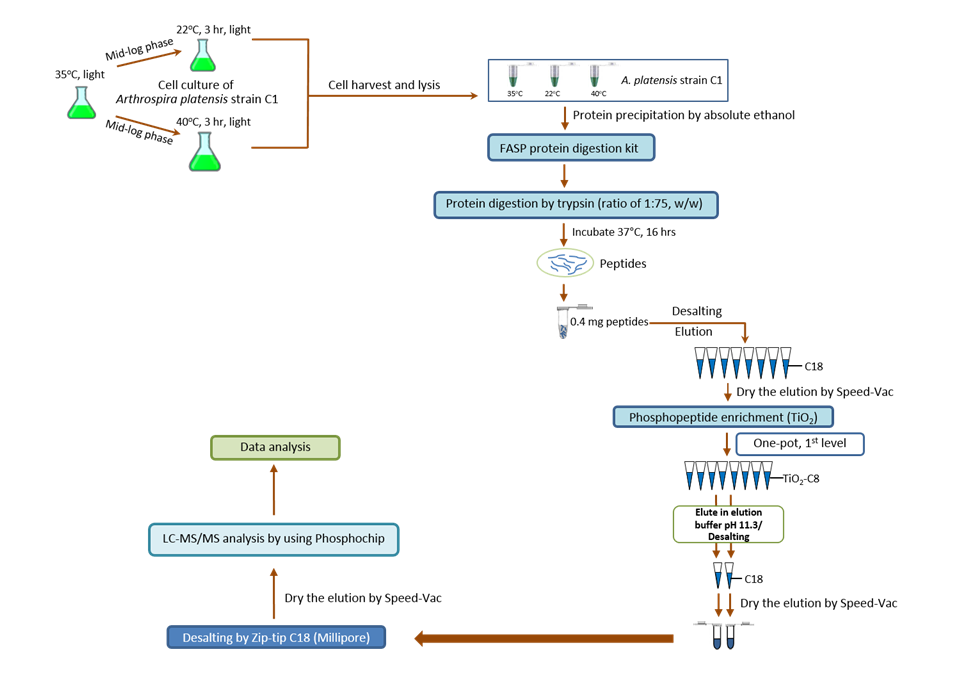

Supplement: Supplementary file 17 — Additional file 17. Experimental design and workflow for the phosphoproteome analysis of Spirulina in response to immediate temperature elevation and reduction. [file 12860_2020_285_MOESM17_ESM.docx]
