# Supplementary material for: Revealing the key point of the temperature stress response of Arthrospira platensis C1 at the interconnection of C- and N- metabolism by proteome analyses and PPI networking
Source: BMC Mol Cell Biol. 2020 Jun 12;21:43. doi: 10.1186/s12860-020-00285-y (PMC7291507; doi:10.1186/s12860-020-00285-y)
Supplement: Supplementary file 12 — Additional file 12. PPI subnetworks of the two multi-sensor histidine kinases, SPLC1_S082010 (orthologous of NIES39_M02160) and SPLC1_S230960 (ortholog of NIES39_L00910), and the glutamate synthase, GlsF. The subnetworks were constructed by using STRING. The A. platensis C1 proteins were inferred to that of the A. platensis NIES39 via orthologous group. [file 12860_2020_285_MOESM12_ESM.docx]

**Additional file 12**


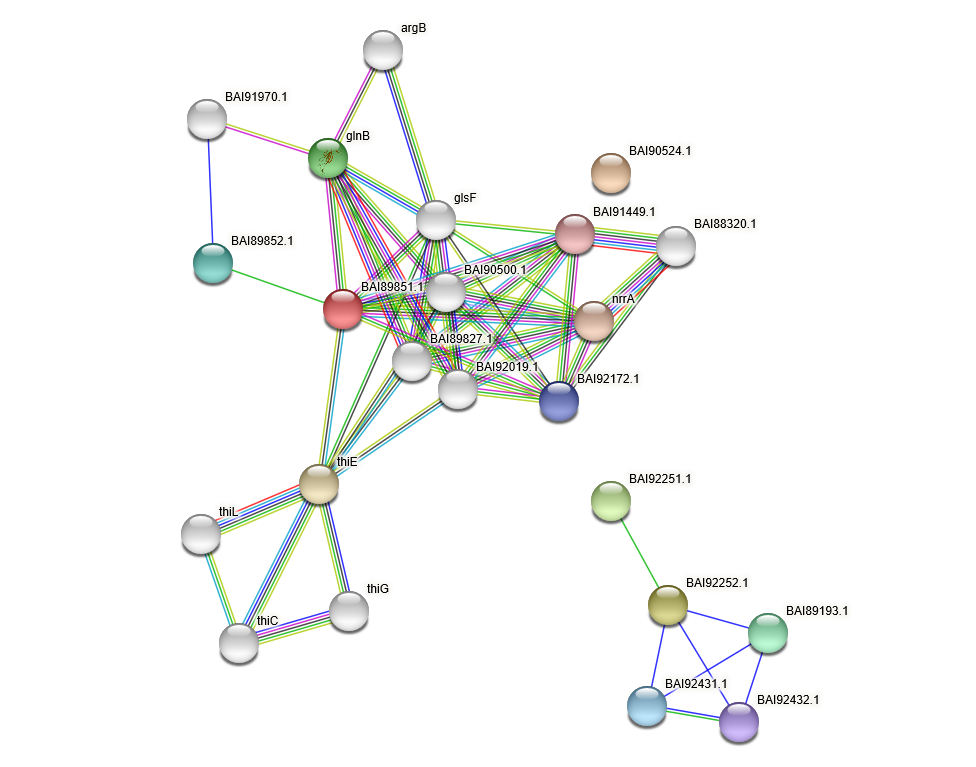


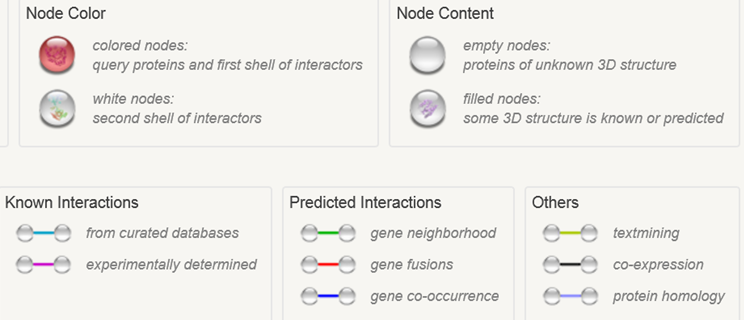


| **node** | **identifier** | **annotation** |
| --- | --- | --- |
| thiC | NIES39_A02970 | Thiamine biosynthesis protein ThiC; Catalyzes the synthesis of the hydroxymethylpyrimidine phosphate (HMP-P) moiety of thiamine from aminoimidazole ribotide (AIR) in a radical S-adenosyl-L-methionine (SAM)-dependent reaction |
| BAI88320.1 | NIES39_A04820 | Sensor histidine kinase, fragment |
| nrrA | NIES39_A06330 | OmpR family response regulator |
| thiL | NIES39_C00280 | Thiamine monophosphate kinase; Catalyzes the ATP-dependent phosphorylation of thiamine- monophosphate (TMP) to form thiamine-pyrophosphate (TPP), the active form of vitamin B1 |
| BAI89193.1 | NIES39_C03260 | Hypothetical protein |
| BAI89827.1 | NIES39_D04090 | Ammonium transporter |
| BAI89851.1 | NIES39_D04330 | Two-component hybrid sensor and regulator |
| BAI89852.1 | NIES39_D04340 | Hypothetical protein |
| BAI90500.1 | NIES39_E02730 | Two-component hybrid sensor and regulator |
| BAI90524.1 | NIES39_E02970 | Two-component hybrid histidine kinase |
| BAI91449.1 | NIES39_J04020 | Two-component response regulator |
| glsF | NIES39_J05540 | Ferredoxin-dependent glutamate synthase |
| glnB | NIES39_K02150 | Nitrogen regulatory protein P-II |
| BAI91970.1 | NIES39_K03240 | Hypothetical protein |
| BAI92019.1 | NIES39_K03730 | Ammonium transporter |
| BAI92172.1 | NIES39_L00110 | Nitrogen assimilation regulatory protein |
| BAI92251.1 | NIES39_L00900 | Pentapeptide repeat-containing protein |
| BAI92252.1 | NIES39_L00910 | Hypothetical protein |
| BAI92431.1 | NIES39_L02720 | Hypothetical protein |
| BAI92432.1 | NIES39_L02730 | Hypothetical protein |
| thiE | NIES39_O04290 | Thiamine-phosphate pyrophosphorylase; Condenses 4-methyl-5-(beta-hydroxyethyl)thiazole monophosphate (THZ-P) and 2-methyl-4-amino-5-hydroxymethyl pyrimidine pyrophosphate (HMP-PP) to form thiamine monophosphate (TMP) |
| argB | NIES39_O06160 | Acetylglutamate kinase |
| thiG | NIES39_R00640 | Thiamine biosynthesis protein; Catalyzes the rearrangement of 1-deoxy-D-xylulose 5- phosphate (DXP) to produce the thiazole phosphate moiety of thiamine. Sulfur is provided by the thiocarboxylate moiety of the carrier protein ThiS. In vitro, sulfur can be provided by H(2)S |
