# Supplementary material for: Revealing the key point of the temperature stress response of Arthrospira platensis C1 at the interconnection of C- and N- metabolism by proteome analyses and PPI networking
Source: BMC Mol Cell Biol. 2020 Jun 12;21:43. doi: 10.1186/s12860-020-00285-y (PMC7291507; doi:10.1186/s12860-020-00285-y)
Supplement: Supplementary file 13 — Additional file 13. PPI subnetwork of the SPLC1_S531000: PleD-like GGDEF-domain containing protein and its client proteins, GlsF, SPLC1_S041070, SPLC1_S082010 and SPLC1_S230960. The subnetwork was constructed by using STRING. The A. platensis C1 proteins were inferred to that of the A. platensis NIES39 via orthologous group. [file 12860_2020_285_MOESM13_ESM.docx]

**Additional file 13**


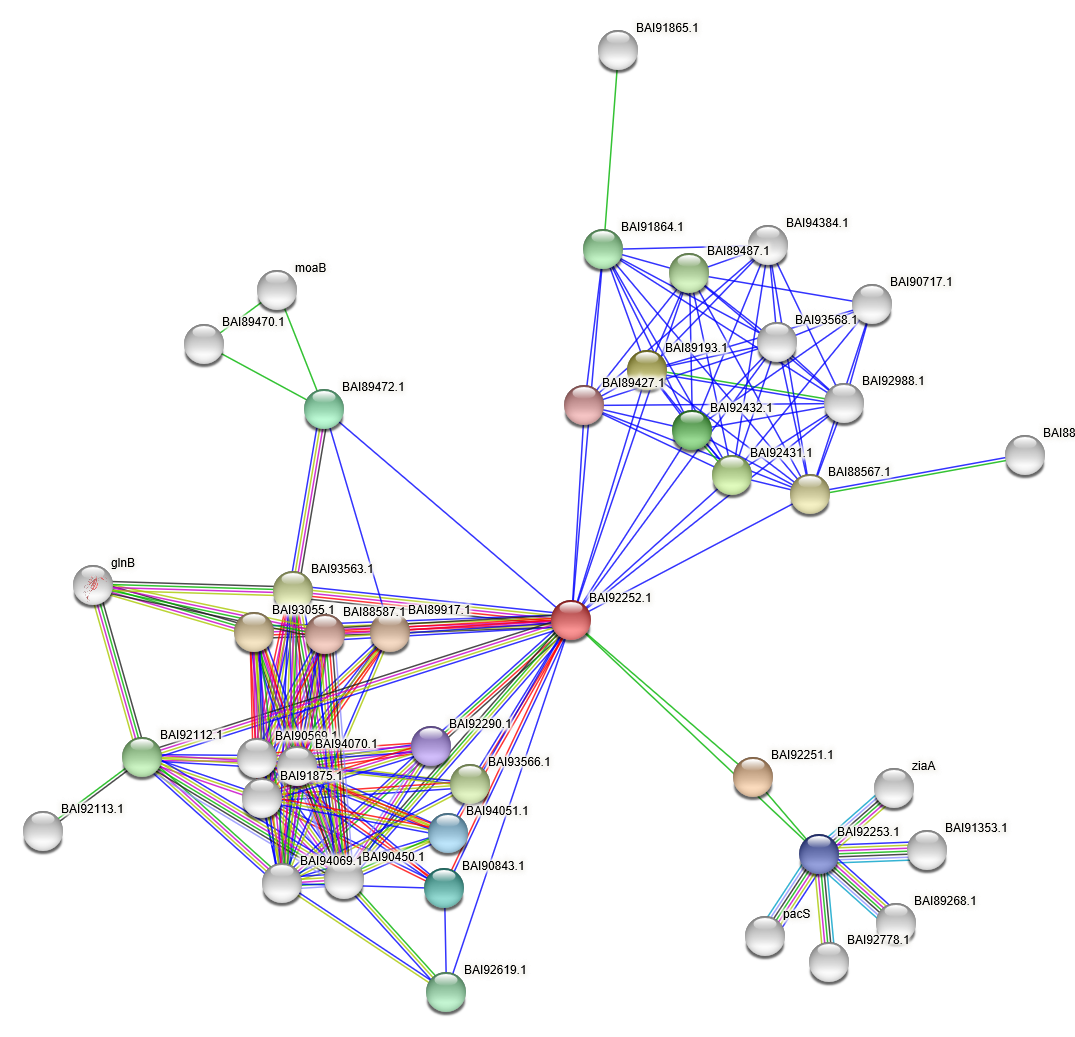


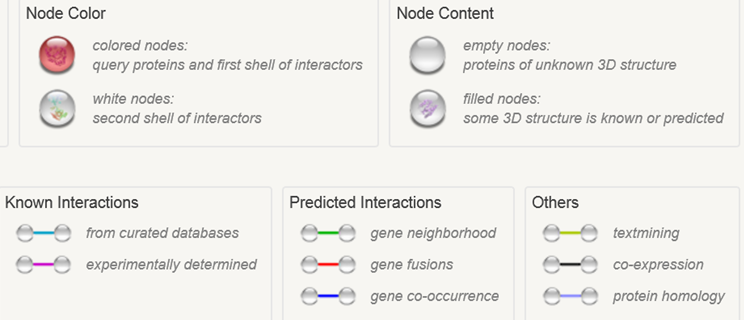


| **node** | **identifier** | **annotation** |
| --- | --- | --- |
| BAI88566.1 | NIES39_A07280 | Hypothetical protein |
| BAI88567.1 | NIES39_A07290 | Hypothetical protein |
| BAI88587.1 | NIES39_A07490 | Putative diguanylate cyclase |
| BAI89193.1 | NIES39_C03260 | Hypothetical protein |
| BAI89268.1 | NIES39_C04020 | Copper-transporting P-type ATPase CtaA |
| BAI89427.1 | NIES39_D00070 | Hypothetical protein |
| BAI89470.1 | NIES39_D00500 | Hypothetical protein |
| moaB | NIES39_D00510 | Molybdopterin precursor biosynthesis protein; May be involved in the biosynthesis of molybdopterin |
| BAI89472.1 | NIES39_D00520 | Hypothetical protein |
| BAI89487.1 | NIES39_D00670 | Hypothetical protein |
| pacS | NIES39_D01970 | Copper-transporting P-type ATPase PacS |
| BAI89917.1 | NIES39_D04990 | Hypothetical protein |
| BAI90450.1 | NIES39_E02220 | Hypothetical protein |
| BAI90569.1 | NIES39_E03420 | Hypothetical protein |
| BAI90717.1 | NIES39_F00240 | Hypothetical protein |
| BAI90843.1 | NIES39_G00600 | Putative diguanylate cyclase |
| BAI91353.1 | NIES39_J03060 | Cation-transporting P-type ATPase |
| glnB | NIES39_K02150 | Nitrogen regulatory protein P-II |
| BAI91864.1 | NIES39_K02170 | Hypothetical protein |
| BAI91865.1 | NIES39_K02180 | Putative esterase |
| BAI91875.1 | NIES39_K02280 | Hypothetical protein |
| BAI92112.1 | NIES39_K04670 | PleD-like protein |
| BAI92113.1 | NIES39_K04680 | Probable pseudouridine synthase |
| BAI92251.1 | NIES39_L00900 | Pentapeptide repeat-containing protein |
| BAI92252.1 | NIES39_L00910 | Hypothetical protein |
| BAI92253.1 | NIES39_L00920 | Cation-transporting P-type ATPase |
| BAI92290.1 | NIES39_L01290 | Putative diguanylate cyclase |
| BAI92431.1 | NIES39_L02720 | Hypothetical protein |
| BAI92432.1 | NIES39_L02730 | Hypothetical protein |
| BAI92619.1 | NIES39_L04620 | Putative diguanylate cyclase |
| BAI92778.1 | NIES39_L06210 | MgtC family protein |
| BAI92988.1 | NIES39_M01510 | Ferrochelatase-like protein; Catalyzes the ferrous insertion into protoporphyrin IX |
| BAI93055.1 | NIES39_M02180 | Putative diguanylate cyclase |
| BAI93563.1 | NIES39_O03140 | PleD-like protein |
| BAI93566.1 | NIES39_O03170 | Hypothetical protein |
| BAI93568.1 | NIES39_O03190 | Hypothetical protein |
| ziaA | NIES39_O04030 | Zinc-transporting P-type ATPase |
| BAI94051.1 | NIES39_Q00430 | Putative diguanylate cyclase |
| BAI94069.1 | NIES39_Q00610 | Hypothetical protein |
| BAI94070.1 | NIES39_Q00620 | Hypothetical protein |
| BAI94384.1 | NIES39_R00750 | Hypothetical protein |
