# Supplementary material for: Revealing the key point of the temperature stress response of Arthrospira platensis C1 at the interconnection of C- and N- metabolism by proteome analyses and PPI networking
Source: BMC Mol Cell Biol. 2020 Jun 12;21:43. doi: 10.1186/s12860-020-00285-y (PMC7291507; doi:10.1186/s12860-020-00285-y)
Supplement: Supplementary file 14 — Additional file 14. Protein sequence alignment of PleD from A. platensis C1 and PleD-like proteins in the same orthologous group. It shows the conserved GGDEF domain in this group of proteins. [file 12860_2020_285_MOESM14_ESM.pdf]

Additional file 14

```
              .........10 .........20 .........30 .........40 .........50
SPLC1_S531000 ---------- ---------- ---------- ---------- ----------
slr0687       ---------- ---------- ---------- ---------- ----------
slr1047       ---------- ---------- ---------- ---------- ----------
slr1798       ----MSGAIP QGPRRIDTLK RREILGCGGS AIMARQDYWA PIHRHELVRW
SYNPCC7002_A084 ---------- ---------- ---------- -----MDFLR LLHSRWL---
slr0829       ---------- -MLQVLLVMA VLVFSALSLD FFGQLNNNVY LFLVLGIIIF
sll0779       MAISAQSFIN VANESLVSTL AIAALGLSLL VILSLAVIQS INRPLRRLQQ
NIES39_N00380 MDLLHRWLHL IKRRFKDYWV ALFIGAIATL AILVIWQKLY IREQRHLAQL
slr0302       ---------- ---------- ---------- ---------- ----------
SPLC1_S490280 ---------- ---------- ---------- ---------- ----------
NIES39_O03140 ---------- ---------- ---------- ---------- ----------
slr1657       ---------- ---------- ---------- ---------- ----------

              .........60 .........70 .........80 .........90 .........100
SPLC1_S531000 ---------- ---------- ---------- ---------- ----------
slr0687       ---------- ---------- ---------- ---------- ----------
slr1047       ---------- ---------- ---------- ---------- ----------
slr1798       IKGSGSASFW QNPLLHNILL NLLVALF--- ---------- ------YVVG
SYNPCC7002_A084 ---------- -----RWFGL NICLAIA--- ---------- ------YAWT
slr0829       LI-------- PALTSLYIAR LVIEPLR--- ---------- ------KLVT
sll0779       QT-------- QALQKSEERF RFALESTDTS WWDWDIKTNE V--DWSDTFD
NIES39_N00380 VQ-------- QEANNIQSIL NRELSSRIVS LNRLAKRWEL SNGTPREFWE
slr0302       ---------- ---------- ---------- ---------- ----------
SPLC1_S490280 ---------- ---------- ---------- ---------- ----------
NIES39_O03140 ---------- ---------- ---------- ---------- ----------
slr1657       ---------- ---------- ---------- ---------- ----------

              .........110 .........120 .........130 .........140 .........150
SPLC1_S531000 ---------- ---------- ---------- ---------- ----------
slr0687       ---------- ---------- ---------- ---------- ----------
slr1047       ---------- ---------- ---------- ---------- ----------
slr1798       IRLSDQLVST LLPGRISPVW ---------- ---------- ----FPSALT
SYNPCC7002_A084 AEISLIFTTL P--GTVASVW ---------- ---------- ----LPSGLT
slr0829       TSQ---NLAQ -GTSHGTITQ ---------- ---------- --PVAIAEIQ
sll0779       AMVGRAPNSY -PKNIASFLS ---------- ---------- --FLHPDDVE
NIES39_N00380 ADVSNYLEDT HGYQVIHWVD PSFIIRWIVP LEGNEQVQDM DLNQEHRQRI
slr0302       ---------- ---------- ---------- ---------- ----------
SPLC1_S490280 ---------- ---------- ---------- ---------- ----------
NIES39_O03140 ---------- ---------- ---------- ---------- ----------
slr1657       ---------- ---------- ---------- ---------- ----------

              .........160 .........170 .........180 .........190 .........200
SPLC1_S531000 ---------- ---------- ---------- ---------- ----------
slr0687       ---------- ---------- ---------- ---------- ----------
slr1047       ---------- ---------- ---------- ---------- ----------
slr1798       FGVFFHF--- ---------- ---------- ---------- ----------
SYNPCC7002_A084 LGLILLF--- ---------- ---------- ---------- ----------
slr0829       QLAKAIN--- -------GMA L--------- ---------- ----------
sll0779       PTRAKLY--- -------DSL ENGIPYKTEF RFVHPDG--T IRWIMAR---
NIES39_N00380 SLQVARDLNQ IILSKHISLV EGGEGFLVII PLFVNERHDG FIVGVFEFTA
slr0302       ---------- ---------- ---------- ---------- ----------
SPLC1_S490280 ---------- ---------- ---------- ---------- ----------
NIES39_O03140 ---------- ---------- ---------- ---------- ----------
slr1657       ---------- ---------- ---------- ---------- ----------
```

```
                .........210 .........220 .........230 .........240 .........250
SPLC1_S531000   ----------   ----------   ----------   ----------   ----------
slr0687         ----------   ----------   ----------   ----------   ----------
slr1047         ----------   ----------   ----------   ----------   ----------
slr1798         ----------   ----------   ----------   ----------   ----------
SYNPCC7002_A084 ----------   ----------   ----------   ----------   ----------
slr0829         ----------   ----------   ----------   ----------   ----------
sll0779         ----------   ----------   GMVQRDAN--   -GQALRMS--   ----------
NIES39_N00380   LFDGILPLSP   LYTTQIYDRG   YLVYSNGDLS   ESPFQQTSLV   RAYGSDWTVQ
slr0302         ----------   ----------   ----------   ----------   ----------
SPLC1_S490280   ----------   ----------   ----------   ----------   ----------
NIES39_O003140  ----------   ----------   ----------   ----------   ----------
slr1657         ----------   ----------   ----------   ----------   ----------

                .........260 .........270 .........280 .........290 .........300
SPLC1_S531000   ----------   ----------   ----------   ----------   ----------
slr0687         ----------   ----------   ----------   ----------   ----------
slr1047         ----------   ----------   ----------   ----------   ----------
slr1798         ----------   ----------   ----------   ----------   ----------
SYNPCC7002_A084 ----------   ----------   ----------   ----------   ----------
slr0829         ----------   ----------   ----------   ----------   ----------
sll0779         ----------   ----------   ----------   ----------   ----------
NIES39_N00380   VSPQPLWIEQ   GKSPLPTIVL   SAGLIIVWTM   ALTVYLGQYS   WRYAHETRKI
slr0302         ----------   ----------   ----------   ----------   ----------
SPLC1_S490280   ----------   ----------   ----------   ----------   ----------
NIES39_O003140  ----------   ----------   ----------   ----------   ----------
slr1657         ----------   ----------   ----------   ----------   ----------

                .........310 .........320 .........330 .........340 .........350
SPLC1_S531000   ----------   ----------   ---MMGSIPE   QTLK----AD   ILV------V
slr0687         ----------   ----------   -------MTF   YQRR----LH   VLL------I
slr1047         ----------   ----------   ----------   ----------   ---------M
slr1798         ---------G   HWVTPGIILG   SVLGLVSVLS   TFDP------   --------PP
SYNPCC7002_A084 ---------G   NKILPSIALG   SLWVISFDLI   ERDP------   --------NI
slr0829         -----RLQQS   LAQTKEELQG   SKELYRQVVQ   SQTD----FI   LRSQPDTTIT
sll0779         --GINLDITE   QKLAEAALKE   NEATLRLALS   AETA----NW   WKWDIVNDQM
NIES39_N00380   NHKLQKEIIH   RQQIEAILLD   SQRQYQTLVE   NSPD----II   QRFDTNFKHI
slr0302         -------MSD   RHKTKAQLLQ   EMERLRADLA   DIQRIAKLGF   WRFDIASGEI
SPLC1_S490280   ----------   ----------   ----MNQLME   DRSK-----I   LWIA--GNVG
NIES39_O003140  ----------   ----------   ----MNELME   DRGK-----I   LWIA--GDVV
slr1657         ----------   ----------   ----------   ----------   ----------
                                          OmpR
                                   YesN superfamily

                .........360 .........370 .........380 .........390 .........400
SPLC1_S531000   DDQPENLHLL   SSLLRQVG--   -DHVR---QS   LNAEMALTSV   QAKLPD--LI
slr0687         EDQQCQVELL   KVLLESQS--   -FFAVQLQVT   RTLAQGVNRL   QSGIFD--TI
slr1047         QNLSLQKALK   KPEVKTL---   ----------   --LQQLGDSL   GK---N--FA
slr1798         SIAQFLVLET   AFAFANTL--   ----------   -QPFIGDLWL   KNKLISVAQK
SYNPCC7002_A084 SIQAFFWVNF   GCIAGNLV--   ----------   -QPLLARFIL   KKYSQSR---
slr0829         FANDALCQAL   GLALPEVIG-   -LQWLDFIVE   EDLKIVLDKI   ACLTPEQPQF
sll0779         YSAPSFHDLL   GRHGDELPTT   WADAIALIYP   EDRAKVQTAV   EATLNHDAPY
NIES39_N00380   YVSPILSQLT   GIPNYTWIGK   -TCRDFPFSA   AMANSWEAAA   NQLLTTGEKQ
slr0302         TWSAETYQLF   GLEPHQFSPS   YDWLVQTIQP   EFRELHQSIA   DKVIATGKTQ
SPLC1_S490280   NDNHSLPQSI   ---LQN----   --NGYEVHLV   IGLKPAYNAI   QSWPLN--LI
NIES39_O003140  NDDHSLPQNI   ---LQN----   --HGYEVNLV   MGLKLAYHAI   QSWPLN--LI
slr1657         ----------   ----------   ----------   ----------   ----------
```

```
                OmpR
                YesN superfamily
                . . . . . . . . . 410 . . . . . . . . . 420 . . . . . . . . . 430 . . . . . . . . . 440 . . . . . . . . . 450
SPLC1_S531000   LLDVRIPGMS G-----YELC RHLKNSENTS KVPIIFLSAL NDIDSIMAGF
slr0687         LLDLFLPDGQ G-----IEAL RTVQKF--AP HIPIIVLTAA TDLNMGLAAL
slr1047         IFDIG----- G-----HCLW GKAQD----S QPKVDIYC-- --HQVLMGTI
slr1798         ESVT------ ---------- ---------- --IIKDSDSI QEPLAILNPF
SYNPCC7002_A084 ---------- ---------- ---------- --------HF FNQVRGVLTY
slr0829         INENQDKRAG GVLGYTQWVS RGIFNDKGQL IEIQSVGRDI SALKETQLEL
sll0779         RVEFRMIPPN GQMVWIADLA ALERDETGRP IQLSGIMIDI TERKQIEEAL
NIES39_N00380   MIEFEAETVN G-WRYLEMAI VPEINEAKNI TSILCISRDV TARKQAETSL
slr0302         TIEYAITRAD ASMGWIWARI EAIIDDRGQV NGLQGVAMDI SDRKMAEIAL
SPLC1_S490280   LLDSKLHDGD S-----YELC NWLKSKAKFQ KIPVIIMESR GDLLNKHTVF
NIES39_O03140   LLDSKLHDGD S-----YELC NWLKSKSKFQ KIPVIIMESR GDLLNKHTVF
slr1657         ---------- ---------- ---------- ---------- ----------

                OmpR
                PRK15347 superfamily
                YesN superfamily
                . . . . . . . . . 460 . . . . . . . . . 470 . . . . . . . . . 480 . . . . . . . . . 490 . . . . . . . . . 500
SPLC1_S531000   QVGGVDY--- ---------- ---------- ---ITKP-FH NSEVLIRVNT
slr0687         QKGAEDY--- ---------- ---------- ---LVKEHLR ESQIAKSILY
slr1047         TGEFTEH--- ---------- ---------- ---SAEA--I AAMLSYIVQT
slr1798         QRVSTTLAFV QGAI------ ---------- -IGPFLSAVI GVTTLLLMGI
SYNPCC7002_A084 ITAATLSPMA SAFI------ ---------- -GVTSIVFAE RMEWG-----
slr0829         RQSQKFIESI AEATPSLLYI YD----HIKQ ANIYSNRSVA EFLGYSPEEI
sll0779         QKSGQRLRMT LESTSTNWWE RD-----LIT DQADWSEQSD HLLGYTPDSY
NIES39_N00380   KLSQAKFEAL ATNMPGMVYS YCPHNSEKPH HFKFVSHHCL DIFELEPNRI
slr0302         RESEARFRNL FESNIVGMFF AS-----NQG EIFDANDRFL QMIGFTKEEL
SPLC1_S490280   DVG------- ---------- ---------- AADYLTQPFH EQE--MLKRI
NIES39_O03140   DVG------- ---------- ---------- AADYITQPFH EQE--MLKRI
slr1657         ---------- ---------- ---------- ---------- ----MTTTEL

                OmpR
                PRK15347 superfamily
                YesN superfamily
                . . . . . . . . . 510 . . . . . . . . . 520 . . . . . . . . . 530 . . . . . . . . . 540 . . . . . . . . . 550
SPLC1_S531000   QLRLYQLQKQ LELQNQSLQE QIRLSQIYLH ---------- ----------
slr0687         ALERKKARRE LQVQIERERL MARILEEIRQ ---------- ----------
slr1047         EYEKKQLAKD TLQKYEEVVF LSQFANAVAT ---------- ----------
Aslr1798        IARENFFYSW ITWWSNSVLA IIVFTPVLIN ---------- ----------
SYNPCC7002_A084 ----QYGLNW FTWWLASTLA HLIFTPVILL ---------- ----------
slr0829         KTMGASLFTN ICHPEDLPRI LAAIENCQKL RDGEILEIEY RVRDARQQWR
sll0779         EKNQDTFYKL -VHPDDREHV QAGVNLAIAT --GEPYQGEF RLVQADGNCI
NIES39_N00380   LADINSLINL -IHEDDLPSL RASMKQSFEQ --FLPWYWQG RVITNSGSIK
slr0302         DLGLVHWDRL -TPPEYDQKD QEIIARLRNH --ETPEPWQK EYYRKDGSRI
SPLC1_S490280   ----GYQITL ---QRQKHQI KEQDLGLNF- ---------- ----------
NIES39_O03140   ----EYQITL ---QRQQHQI KEQDLGLTW- ---------- ----------
slr1657         -----TAATL TQLQQEINRL QRENDDLHIA ---------- ----------
```

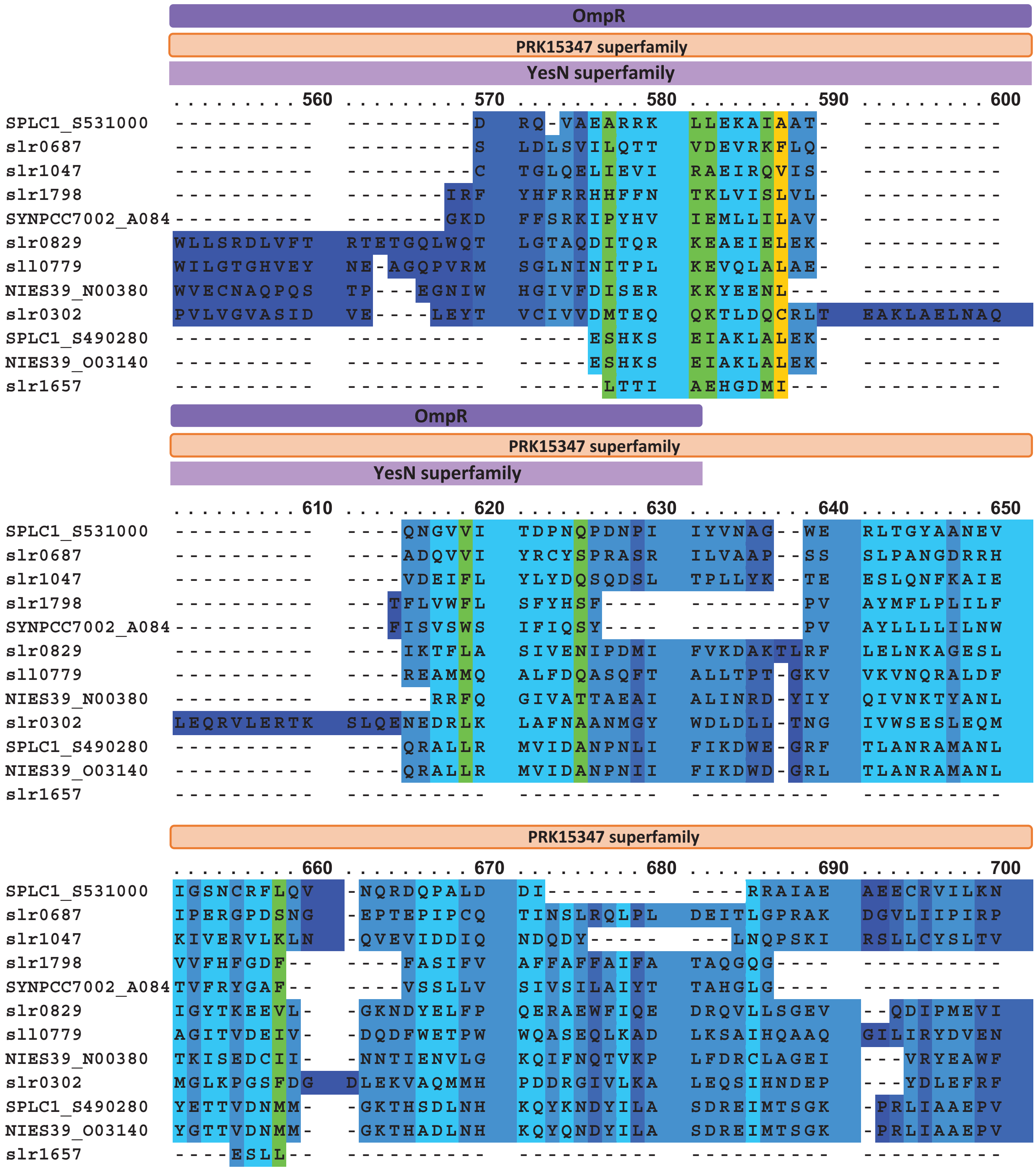
OmpR
PRK15347 superfamily
YesN superfamily
560 570 580 590 600
SPLC1_S531000 ---------- ---------D RQ-VAEARRK LLEKAIAAT- ----------
slr0687 ---------- ---------S LDLSVILQTT VDEVRKFLQ- ----------
slr1047 ---------- ---------C TGLQELIEVI RAEIRQVIS- ----------
slr1798 ---------- -------IRF YHFRRHHFFN TKLVISLVL- ----------
SYNPCC7002_A084 ---------- -------GKD FFSRKIPYHV IEMLLILAV- ----------
slr0829 WLLSRDLVFT RTETGQLWQT LGTAQDITQR KEAEIELEK- ----------
sll0779 WILGTGHVEY NE-AGQPVRM SGLNINITPL KEVQLALAE- ----------
NIES39_N00380 WVECNAQPQS TP---EGNIW HGIVFDISER KKYEENL--- ----------
slr0302 PVLVGVASID VE----LEYT VCIVVDMTEQ QKTLDQCRLT EAKLAELNAQ
SPLC1_S490280 ---------- ---------- -----ESHKS EIAKLALEK- ----------
NIES39_O03140 ---------- ---------- -----ESHKS EIAKLALEK- ----------
slr1657 ---------- ---------- ------LTTI AEHGDMI--- ----------
OmpR
PRK15347 superfamily
YesN superfamily
610 620 630 640 650
SPLC1_S531000 ---------- ----QNGVVI TDPNQPDNPI IYVNAG--WE RLTGYAANEV
slr0687 ---------- ----ADQVVI YRCYSPRASR ILVAAP--SS SLPANGDRRH
slr1047 ---------- ----VDEIFL YLYDQSQDSL TPLLYK--TE ESLQNFKAIE
slr1798 ---------- ---TFLVWFL SFYHSF---- --------PV AYMFLPLILF
SYNPCC7002_A084 ---------- ---FISVSWS IFIQSY---- --------PV AYLLLLILNW
slr0829 ---------- ----IKTFLA SIVENIPDMI FVKDAKTLRF LELNKAGESL
sll0779 ---------- ----REAMMQ ALFDQASQFT ALLTPT-GKV VKVNQRALDF
NIES39_N00380 ---------- ------RRFQ GIVATTAEAI ALINRD-YIY QIVNKTYANL
slr0302 LEQRVLERTK SLQENEDRLK LAFNAANMGY WDLDLL-TNG IVWSESLEQM
SPLC1_S490280 ---------- ----QRALLR MVIDANPNLI FIKDWE-GRF TLANRAMANL
NIES39_O03140 ---------- ----QRALLR MVIDANPNII FIKDWD-GRL TLANRAMANL
slr1657 ---------- ---------- ---------- ---------- ----------
PRK15347 superfamily
660 670 680 690 700
SPLC1_S531000 IGSNCRFLQV -NQRDQPALD DI-------- ----RRAIAE AEECRVILKN
slr0687 IPERGPDSNG -EPTEPIPCQ TINSLRQLPL DEITLGPRAK DGVLIIPIRP
slr1047 KIVERVLKLN -QVEVIDDIQ NDQDY----- ---LNQPSKI RSLLCYSLTV
slr1798 VVFHFGDF-- ----FASIFV AFFAFFAIFA TAQGQG---- ----------
SYNPCC7002_A084 TVFRYGAF-- ----VSSLLV SIVSILAIYT TAHGLG---- ----------
slr0829 IGYTKEEVL- -GKNDYELFP QERAEWFIQE DRQVLLSGEV --QDIPMEVI
sll0779 AGITVDEIV- -DQDFWETPW WQASEQLKAD LKSAIHQAAQ GILIRYDVEN
NIES39_N00380 TKISEDCII- -NNTIENVLG KQIFNQTVKP LFDRCLAGEI ---VRYEAWF
slr0302 MGLKPGSFDG DLEKVAQMMH PDDRGIVLKA LEQSIHNDEP ---YDLEFRF
SPLC1_S490280 YETTVDNMM- -GKTHSDLNH KQYKNDYILA SDREIMTSGK -PRLIAAEPV
NIES39_O03140 YGTTVDNMM- -GKTHADLNH KQYQNDYILA SDREIMTSGK -PRLIAAEPV
slr1657 ----ESLL-- ---------- ---------- ---------- ----------

PRK15347 superfamily

FhlA
GAF superfamily

```
             .........710 .........720 .........730 .........740 .........750
SPLC1_S531000 YRRDGTIFWN ELFISPVRNE PGELTNFIGI QTDVTERKKS EEALERSR--
slr0687       QKPHQEDCLW GQLVVRVGD- --QKRSWLPW EIEFLCHLSS QVAIAIQQ--
slr1047       Q--NSIIGVL GLAHYQVKHF DSSDLNLFST LTGQVAAAIR TAQYYETIKN
slr1798       ---------- ---------- ---LFNQDSG NNSIIFLQLF TAVISMTALF
SYNPCC7002_A084---------- ---------- ---VFVLESQ NQSLLFLQSF MGVFALSSLL
slr0829       QTCHEGIRIL HTKKIPICDA SGQAQYLLGI SADITDLLES QQRLQELARH
sll0779       LGNNGQKVIL DFSIRPIYDT DQRIIFLLCE GRDITDKFRI QEALAESEER
NIES39_N00380 NF-PGKPRFI GATYSPILQP DNTVKEVIVT VRDLTSLKQA EIAMKKQSDR
slr0302       IKPDGTLRWA ASQATVIRDQ NNVPLRVIGV DVDITRRKQF EEELQKVNQV
SPLC1_S490280 RLNNGEVRWF QTNKIPLIGN DGQTPYLLVV STDITERQEA EQELWSHAER
NIES39_O03140 RLKNGVVRWF QTNKIPLIGN DGQTPYLLVV STDITERQEA EQELWSHAER
slr1657       ---------- ---------- -------NET NVKLRSEILE RQRAEAKLQN
```

FhlA
GAF superfamily

```
             .........760 .........770 .........780 .........790 .........800
SPLC1_S531000 ---------- ---------- ---------- ---------- ----------
slr0687       ---------- ---------- ---------- ---------- ----------
slr1047       YSQTLEIRVK ERTM------ ---------- ---------- ----------
slr1798       FSAIIQE--- ---------- ---------- ---------- ----------
SYNPCC7002_A084LSAVVEE--- ---------- ---------- ---------- ----------
slr0829       IPGVIYQ--- F--------- ---------- --------RM RPDGTFH---
sll0779       FRQTFQTTAV S--------- ---------- --------SA LISLDGK---
NIES39_N00380 QDILSKVTNR IRRSLHLQDI LNTTVVEVSK VLGNHIVLVC KFQLNWS---
slr0302       LEERLGELKQ RNAEMLILSG ITDYLQSCFT VKDACGVIAA LAQPLFPDCC
SPLC1_S490280 ERMLRAIVQS LHECIDLEQV LECTVNQIRE FLVSDRALIY RLENDGNYRV
NIES39_O03140 ERMLRAIVQS IHECLDLEQV LECTVNQIRE FLVSDRALIY RLENDGNYRV
slr1657       ILSLISR--- ---------- ---------- ---------- ----------
```

FhlA
GAF superfamily

```
             .........810 .........820 .........830 .........840 .........850
SPLC1_S531000 ---------- ---------- ---------- ---------- ----------
slr0687       ---------- ---------- ---------- ---------- ----------
slr1047       ---------- ---------- ---------- ---------- ----------
slr1798       ---------K KIA------- ----QQSLSR AVKN------ ----------
SYNPCC7002_A084---------R SRA------- ----QTSLKK ALDN------ ----------
slr0829       -FPYASEGIR KIYGVSPEEV KEDATPVFNV LHPDELPGVS RS--------
sll0779       -FLEVNPAFC ELLGYTSDEL QDHDVAEVTD --PNWVPFQG DL--------
NIES39_N00380 -HKIIAHSEE LCRDYHRLFC VIDPVMTIKI DQMIWQIAQL CRSGQ----I
slr0302       GGIFILSAQN NCLERVTFWG NPFCSDDVFT PLDCWALRRG RSHGVKQGQH
SPLC1_S490280 VMESVSPPWQ PLLGTTINYS G--LSEVITE MAAANYLKIW SISNVDKANI
NIES39_O03140 VMESVSPPWQ PLLGTAINYS G--LSEVITE MAAANYIKIW SISDVDKAHI
slr1657       ---------E KED------- ---------- ---------- ----------
```

FhlA
GAF superfamily

```
             .........860 .........870 .........880 .........890 .........900
SPLC1_S531000 ---------- ---------- ---------- ---------- ----------
slr0687       ---------- ---------- ---------- ---------- ----------
slr1047       ---------- ---------- ---------- ---------- ----------
slr1798       -LENEVQK-- ---------- ---------- ---------- ----------
SYNPCC7002_A084-STKLILE-- ---------- ---------- ---------- ----------
slr0829       -IYESAANL- TPWYYEYRVC FADGR----- -------VIW VLGYATPRRE
sll0779       -TQQLLNREI MAYTQERRYQ HRDGR----- -------WIW GLLNVSLVRD
NIES39_N00380 NASVEMWQQF QGPTHLIFPI WLEDD----- -------ELW GLLIAYFSSE
slr0302       ALFCNHVNDQ HLPLASLCIP LIAQG----- -------ETL GLLFLCTFKG
SPLC1_S490280 PINLRNKLDG LQVKSKLILP IIHRHPDLRS QPTESPNCLW GLLVIHQCDN
NIES39_O03140 PINLRDKLED LQVKSKLILP IIHRHPDLPS GGTESPHCLW GLLVIHQCDN
slr1657       -LEIIVET-- ---------- ---------- ---------- ----------
```

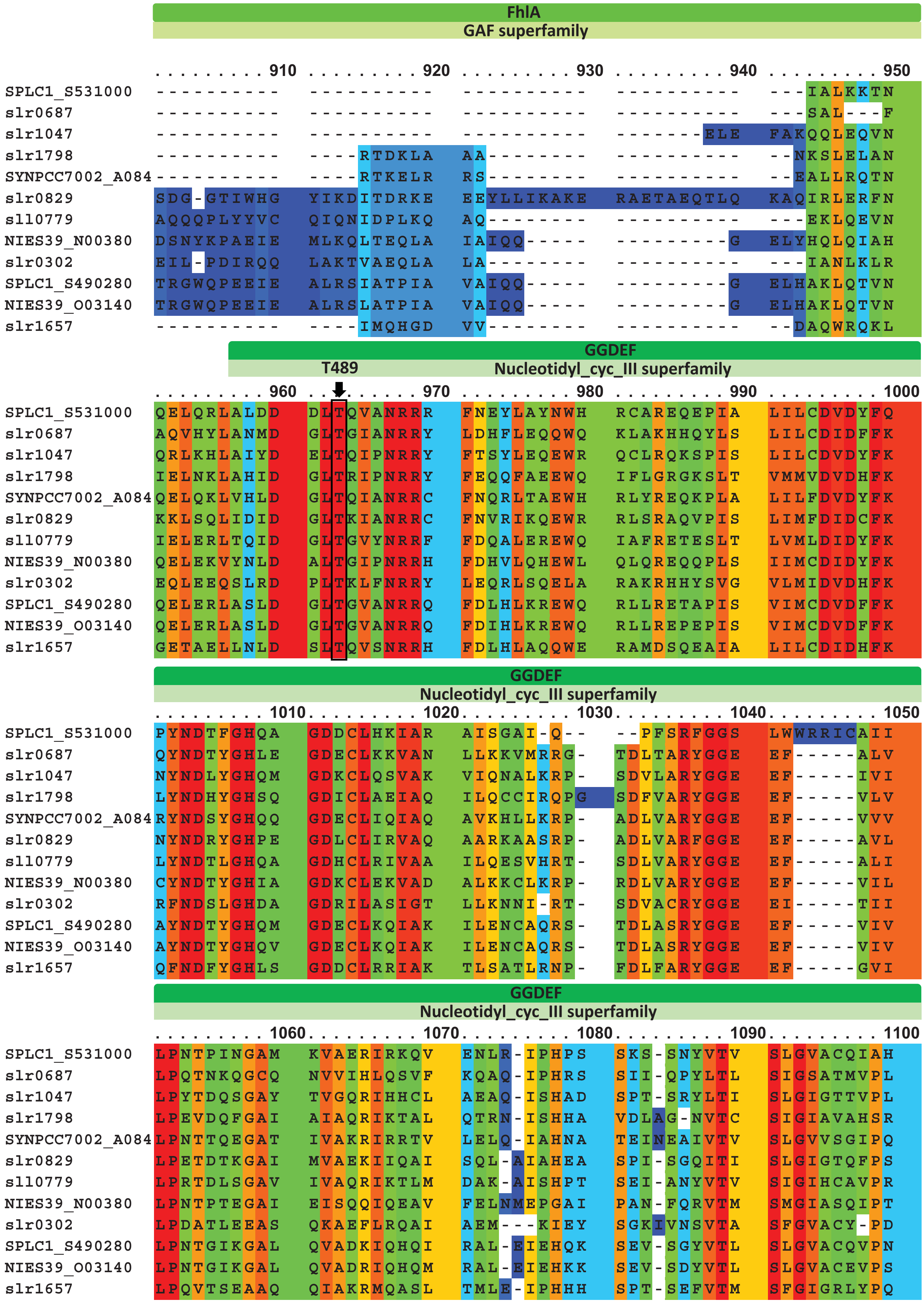

FhlA
GAF superfamily
910 920 930 940 950
SPLC1_S531000 ---------- ---------- ---------- ---------- ---IALKKTN
slr0687 ---------- ---------- ---------- ---------- ---SAL---F
slr1047 ---------- ---------- ---------- -------ELE FAKQQLEQVN
slr1798 ---------- ----RTDKLA AA-------- ---------- --NKSLELAN
SYNPCC7002_A084 ---------- ----RTKELR RS-------- ---------- --EALLRQTN
slr0829 SDG-GTIWHG YIKDITDRKE EEYLLIKAKE RAETAEQTLQ KAQIRLERFN
sll0779 AQQQPLYYVC QIQNIDPLKQ AQ-------- ---------- ---EKLQEVN
NIES39_N00380 DSNYKPAEIE MLKQLTEQLA IAIQQ----- ---------G ELYHQLQIAH
slr0302 EIL-PDIRQQ LAKTVAEQLA LA-------- ---------- ---IANLKLR
SPLC1_S490280 TRGWQPEEIE ALRSIATPIA VAIQQ----- ---------G ELHAKLQTVN
NIES39_003140 TRGWQPEEIE ALRSLATPIA VAIQQ----- ---------G ELHAKLQTVN
slr1657 ---------- ----IMQHGD VV-------- ---------- --DAQWRQKL
GGDEF
T489
Nucleotidyl_cyc_III superfamily
960 970 980 990 1000
SPLC1_S531000 QELQRLALDD DLTQVANRRR FNEYLAYNWH RCAREQEPIA LILCDVDYFQ
slr0687 AQVHYLANMD GLTGIANRRY LDHFLEQQWQ KLAKHHQYLS LILCDIDFFK
slr1047 QRLKHLAIYD ELTQIPNRRY FTSYLEQEWR QCLRQKSPIS LILCDVDYFK
slr1798 IELNKLAHID GLTRIPNRRY FEQQFAEEWQ IFLGRGKSLT VMMVDVDHFK
SYNPCC7002_A084 QELQKLVHLD GLTQIANRRC FNQRLTAEWH RLYREQKPLA LILFDVDYFK
slr0829 KKLSQLIDID GLTKIANRRC FNVRIKQEWR RLSRAQVPIS LIMFDIDCFK
sll0779 IELERLTQID GLTGVYNRRF FDQALEREWQ IAFRETESLT LVMLDIDYFK
NIES39_N00380 QELEKVYNLD ALTGIPNRRH FDHVLQHEWL QLQREQQPLS IIMCDIDYFK
slr0302 EQLEEQSLRD PLTKLFNRRY LEQRLSQELA RAKRHHYSVG VLMIDVDHFK
SPLC1_S490280 QELERLASLD GLTGVANRRQ FDLHLKREWQ RLLRETAPIS VIMCDVDFFK
NIES39_003140 QELERLASLD GLTGVANRRQ FDIHLKREWQ RLLREPEPIS VIMCDVDFFK
slr1657 GETAELLNLD SLTQVSNRRH FDLHLAQQWE RAMDSQEAIA LILCDIDHFK
GGDEF
Nucleotidyl_cyc_III superfamily
1010 1020 1030 1040 1050
SPLC1_S531000 PYNDTFGHQA GDDCLHKIAR AISGAI-Q-- --PFSRFGGS LWWRRICAII
slr0687 QYNDTYGHLE GDECLKKVAN LLKKVMRRG- TDLTARYGGE EF-----ALV
slr1047 NYNDLYGHQM GDKCLQSVAK VIQNALKRP- SDVLARYGGE EF-----IVI
slr1798 LYNDHYGHSQ GDICLAEIAQ ILQCCIRQPG SDFVARYGGE EF-----VLV
SYNPCC7002_A084 RYNDSYGHQQ GDECLIQIAQ AVKHLLKRP- ADLVARYGGE EF-----VVV
slr0829 NYNDRYGHPE GDLCLIRVAQ AARKAASRP- ADLVARFGGE EF-----AVL
sll0779 LYNDTLGHQA GDHCLRIVAA ILQESVHRT- SDLVARYGGE EF-----ALI
NIES39_N00380 CYNDTYGHIA GDKCLEKVAD ALKKCLKRP- RDLVARYGGE EF-----VIL
slr0302 RFNDSLGHDA GDRILASIGT LLKNNI-RT- SDVACRYGGE EI-----TII
SPLC1_S490280 AYNDTYGHQM GDECLKQIAK ILENCAQRS- TDLASRYGGE EF-----VIV
NIES39_003140 AYNDTYGHQV GDECLKQIAK ILENCAQRS- TDLASRYGGE EF-----VIV
slr1657 QFNDFYGHLS GDDCLRRIAK TLSATLRNP- FDLFARYGGE EF-----GVI
GGDEF
Nucleotidyl_cyc_III superfamily
1060 1070 1080 1090 1100
SPLC1_S531000 LPNTPINGAM KVAERIRKQV ENLR-IPHPS SKS-SNYVTV SLGVACQIAH
slr0687 LPQTNKQGCQ NVVIHLQSVF KQAQ-IPHRS SII-QPYLTL SIGSATMVPL
slr1047 LPYTDQSGAY TVGQRIHHCL AEAQ-ISHAD SPT-SRYLTI SLGIGTTVPL
slr1798 LPEVDQFGAI AIAQRIKTAL QTRN-ISHHA VDLAG-NVTC SIGIAVAHSR
SYNPCC7002_A084 LPNTTQEGAT IVAKRIRRTV LELQ-IAHNA TEINEAIVTV SLGVVSGIPQ
slr0829 LPETDTKGAI MVAEKIIQAI SQL-AIAHEA SPI-SGQITI SLGIGTQFPS
sll0779 LPRTDLSGAV IVAQRIKTLM DAK-AISHPT SEI-ANYVTV SIGIHCAVPR
NIES39_N00380 LPNTPTEGAI EISQQIQEAV FELNMEPGAI PAN-FQRVTM SMGIASQIPT
slr0302 LPDATLEEAS QKAEFLRQAI AEM---KIEY SGKIVNSVTA SFGVACY-PD
SPLC1_S490280 LPNTGIKGAL QVADKIQHQI RAL-EIEHQK SEV-SGYVTL SLGVACQVPN
NIES39_003140 LPNTGIKGAL QVADRIQHQM RAL-EIEHKK SEV-SDYVTL SLGVACEVPS
slr1657 LPQVTSEAAQ QIAKRMQASL TMLE-IPHHH SPT-SEFVTM SFGIGRLYPQ

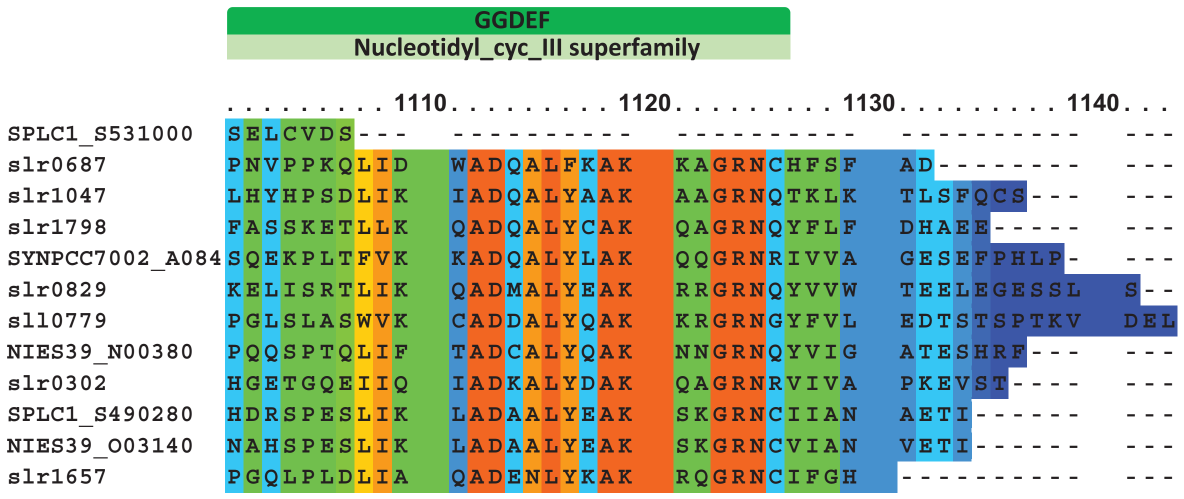
GGDEF
Nucleotidyl_cyc_III superfamily
..........1110..........1120..........1130..........1140...
SPLC1_S531000 SELCVDS--- ---------- ---------- ---------- ---
slr0687 PNVPPKQLID WADQALFKAK KAGRNCHFSF AD-------- ---
slr1047 LHYHPSDLIK IADQALYAAK AAGRNQTKLK TLSFQCS--- ---
slr1798 FASSKETLLK QADQALYCAK QAGRNQYFLF DHAEE----- ---
SYNPCC7002_A084 SQEKPLTFVK KADQALYLAK QQGRNRIVVA GESEFPHLP- ---
slr0829 KELISRTLIK QADMALYEAK RRGRNQYVVW TEELEGESSL S--
sll0779 PGLSLASWVK CADDALYQAK KRGRNGYFVL EDTSTSPTKV DEL
NIES39_N00380 PQQSPTQLIF TADCALYQAK NNGRNQYVIG ATESHRF--- ---
slr0302 HGETGQEIIQ IADKALYDAK QAGRNRVIVA PKEVST---- ---
SPLC1_S490280 HDRSPESLIK LADAALYEAK SKGRNCIIAN AETI------ ---
NIES39_O03140 NAHSPESLIK LADAALYEAK SKGRNCVIAN VETI------ ---
slr1657 PGQLPLDLIA QADENLYKAK RQGRNCIFGH ---------- ---
