# Supplementary material for: Revealing the key point of the temperature stress response of Arthrospira platensis C1 at the interconnection of C- and N- metabolism by proteome analyses and PPI networking
Source: BMC Mol Cell Biol. 2020 Jun 12;21:43. doi: 10.1186/s12860-020-00285-y (PMC7291507; doi:10.1186/s12860-020-00285-y)
Supplement: Supplementary file 15 — Additional file 15. PPI subnetworks of the proteins in the same orthologous group as (A) SPLC1_S082010, (B) SPLC1_S540750 and (C) SPLC1_S360070. The subnetworks were constructed by using STRING. The A. platensis C1 proteins were inferred to that of the A. platensis NIES39 via orthologous group. [file 12860_2020_285_MOESM15_ESM.docx]

**Additional file 15**


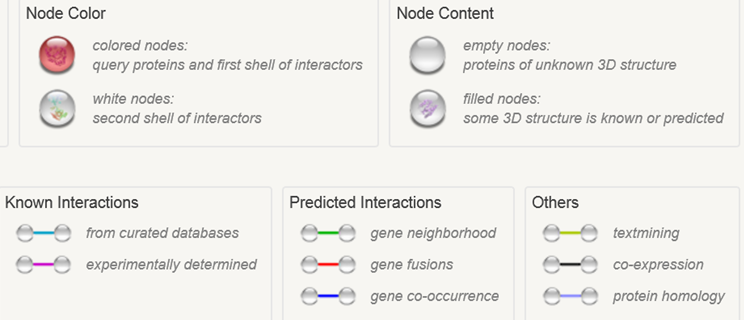


**(A)**

**
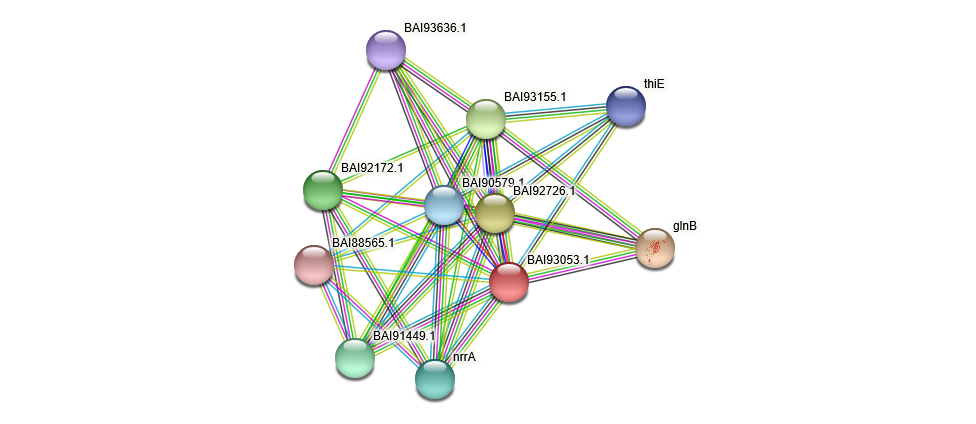
**

| **node** | **identifier** | **annotation** |
| --- | --- | --- |
| nrrA | NIES39_A06330 | OmpR family response regulator |
| BAI88565.1 | NIES39_A07270 | 3-hydroxyisobutyrate dehydrogenase |
| BAI90579.1 | NIES39_E03520 | Hypothetical protein |
| BAI91449.1 | NIES39_J04020 | Two-component response regulator |
| glnB | NIES39_K02150 | Nitrogen regulatory protein P-II |
| BAI92172.1 | NIES39_L00110 | Nitrogen assimilation regulatory protein |
| BAI92726.1 | NIES39_L05690 | Putative sensor protein |
| BAI93053.1 | NIES39_M02160 | Two-component hybrid histidine kinase |
| BAI93155.1 | NIES39_N00380 | PleD-like protein |
| BAI93636.1 | NIES39_O03890 | Two-component response regulator |
| thiE | NIES39_O04290 | Thiamine-phosphate pyrophosphorylase; Condenses 4-methyl-5-(beta-hydroxyethyl)thiazole monophosphate (THZ-P) and 2-methyl-4-amino-5-hydroxymethyl pyrimidine pyrophosphate (HMP-PP) to form thiamine monophosphate (TMP) |

**(B)**

**
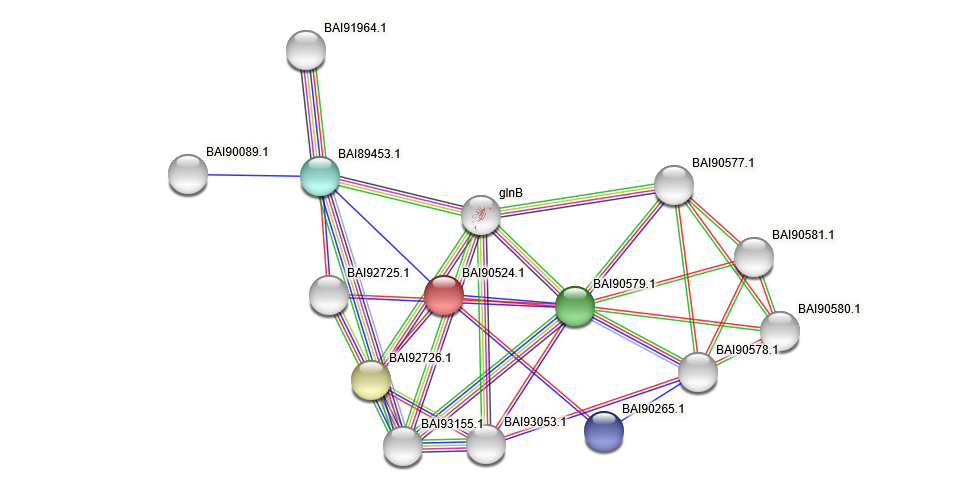
**

| **node** | **identifier** | **annotation** |
| --- | --- | --- |
| BAI89453.1 | NIES39_D00330 | Two-component sensor histidine kinase |
| BAI90089.1 | NIES39_D06720 | Two-component response regulator |
| BAI90265.1 | NIES39_E00300 | Putative PAS/PAC sensor protein |
| BAI90524.1 | NIES39_E02970 | Two-component hybrid histidine kinase |
| BAI90577.1 | NIES39_E03500 | Hypothetical protein |
| BAI90578.1 | NIES39_E03510 | Hypothetical protein |
| BAI90579.1 | NIES39_E03520 | Hypothetical protein |
| BAI90580.1 | NIES39_E03530 | Hypothetical protein |
| BAI90581.1 | NIES39_E03540 | Hypothetical protein |
| glnB | NIES39_K02150 | Nitrogen regulatory protein P-II |
| BAI91964.1 | NIES39_K03180 | Two-component response regulator |
| BAI92725.1 | NIES39_L05680 | Two-component sensor histidine kinase |
| BAI92726.1 | NIES39_L05690 | Putative sensor protein |
| BAI93053.1 | NIES39_M02160 | Two-component hybrid histidine kinase |
| BAI93155.1 | NIES39_N00380 | PleD-like protein |

**(C)**

**
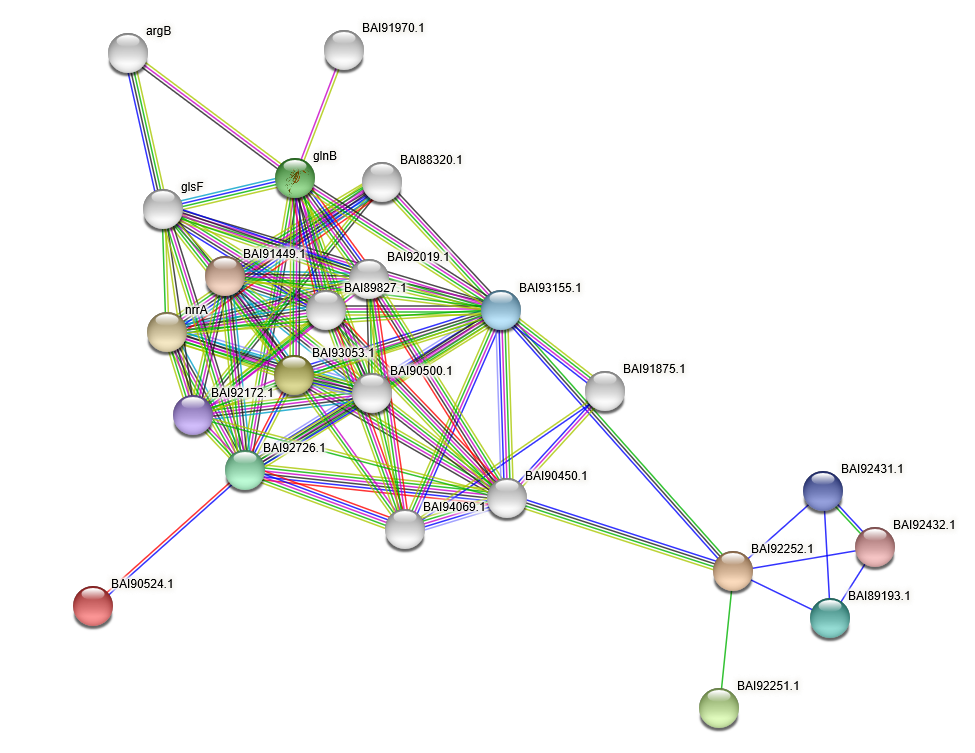
**

| **node** | **identifier** | **annotation** |
| --- | --- | --- |
| BAI88320.1 | NIES39_A04820 | Sensor histidine kinase, fragment |
| nrrA | NIES39_A06330 | OmpR family response regulator |
| BAI89193.1 | NIES39_C03260 | Hypothetical protein |
| BAI89827.1 | NIES39_D04090 | Ammonium transporter |
| BAI90450.1 | NIES39_E02220 | Hypothetical protein |
| BAI90500.1 | NIES39_E02730 | Two-component hybrid sensor and regulator |
| BAI90524.1 | NIES39_E02970 | Two-component hybrid histidine kinase |
| BAI91449.1 | NIES39_J04020 | Two-component response regulator |
| glsF | NIES39_J05540 | Ferredoxin-dependent glutamate synthase |
| glnB | NIES39_K02150 | Nitrogen regulatory protein P-II |
| BAI91875.1 | NIES39_K02280 | Hypothetical protein |
| BAI91970.1 | NIES39_K03240 | Hypothetical protein |
| BAI92019.1 | NIES39_K03730 | Ammonium transporter |
| BAI92172.1 | NIES39_L00110 | Nitrogen assimilation regulatory protein |
| BAI92251.1 | NIES39_L00900 | Pentapeptide repeat-containing protein |
| BAI92252.1 | NIES39_L00910 | Hypothetical protein |
| BAI92431.1 | NIES39_L02720 | Hypothetical protein |
| BAI92432.1 | NIES39_L02730 | Hypothetical protein |
| BAI92726.1 | NIES39_L05690 | Putative sensor protein |
| BAI93053.1 | NIES39_M02160 | Two-component hybrid histidine kinase |
| BAI93155.1 | NIES39_N00380 | PleD-like protein |
| argB | NIES39_O06160 | Acetylglutamate kinase |
| BAI94069.1 | NIES39_Q00610 | Hypothetical protein |
