# Supplementary material for: Revealing the key point of the temperature stress response of Arthrospira platensis C1 at the interconnection of C- and N- metabolism by proteome analyses and PPI networking
Source: BMC Mol Cell Biol. 2020 Jun 12;21:43. doi: 10.1186/s12860-020-00285-y (PMC7291507; doi:10.1186/s12860-020-00285-y)
Supplement: Supplementary file 18 — Additional file 18. List of primers used for gene cloning, site-directed mutagenesis and construction of Sll0474 deletion mutant. [file 12860_2020_285_MOESM18_ESM.docx]

**Additional file 18**

| **No.** | **ORF** | **Protein name** | **Primer name** | **Oligonucleotide primers (5' to 3')** | **Size (bp)** | **PCR condition** |
| --- | --- | --- | --- | --- | --- | --- |
| 1 | SPLC1_  S041070 | Multi-sensor Hybrid Histidine Kinase | 1SPL041070BmIFW  SPL41070PsIRV | CGGAATTCATGGCAAGCAACTCAT CA  GGGCTGCAGTTATAAGTCTAAAAT TTCCTG | 5,337 | Initial denaturation;  95 °C, 3 min  Cycle 1-30;  95 °C 30 sec  50 °C 45 sec  68 °C 6 min |
| 2 | SPLC1_  S041070S976A | Multi-sensor Hybrid Histidine Kinase (Mutant S976A) | SPL41070_  S976AFW  SPL41070_  S976ARV | CAATGGTTAGGATACGCTCGTGAACAATTA  CAGTTCCGTATCATTAACTTTGAC ATAGCG | 5,337 | Initial denaturation;  98 °C, 3 min  Cycle 1-30;  98 °C 30 sec  65 °C 45 sec  72 °C 5 min |
| 3 | SPLC1_  S490280 | Response regulator receiver modulated diguanylate cyclase (PleD) | AP07540011  FW  AP07540011_RV | GAATTCATGAATCAGTTAATGGAA GACC  TGGGGATCCTTAAATGGTCTCAGC A | 1,959 | Initial denaturation;  95 °C, 3 min  Cycle 1-30;  95 °C 30 sec  50 °C 45 sec  68 °C 2 min |
| 4 | SPLC1_  S490280T489A | Response regulator receiver modulated diguanylate cyclase (PleD) (Mutant T489A) | SPL490280_  T489AFW  SPL490280_ T489ARV | TAGACGGATTAGCCGGAGTGGCGA AT  GGCTGGCTAGGCGTTCCAATTCTT G | 1,959 | Initial denaturation;  98 °C, 3 min  Cycle 1-30;  98 °C 30 sec  65 °C 45 sec  72 °C 3 min |
| 5 | SPLC1S240280 | Serine/threonine protein kinase with TPR repeat protein | S240280Fw  S240280Rv | CGG GGA TCC ATG AAC TTA GAC GAT  GCCAAGCTTCTTCGTTACTGTAAT CG | 2,265 | Initial denaturation; 94°C, 3 min  Cycle 1-34;  94°C, 30 sec  50°C, 45 sec  68°C, 3 min |
| 6 | SPLC1_  S240280S254A | Serine/threonine protein kinase with TPR repeat protein (Mutant S254A) | SPL240280_  S254AFW  SPL240280_  S254ARV | CTCCAAGATCCTGCCCTATCTATA TCTGGA    TTTGGGCAAATCTGAGCCTTCTAA ACC | 2,265 | Initial denaturation;  98 °C, 3 min  Cycle 1-30;  98 °C 30 sec  65 °C 45 sec  72 °C 3 min |
| 7 | SPLC1_  S240860 | Hybrid sensory kinase | AP07310007_FW  AP07310007  RV | CATATGATGAATCACAATCACCGT CAAT  GGATCCCTACTTTGGCTTGGTTAA ATC | 2,658 | Initial denaturation;  95 °C, 3 min  Cycle 1-30;  95 °C 30 sec  50 °C 45 sec  68 °C 3 min |
| 8 | SPLC1_  S540750 | Similar sensory box histidine kinase/response regulator (Hik21) | AP07430015  FW  AP07430015  RV | GAATTCATGGTTTTAACAGTAATG GCAAT  TGGGGATCCTCAGCTATAAAATTT TTGTT | 3,729 | Initial denaturation;  95 °C, 3 min  Cycle 1-30;  95 °C 30 sec  50 °C 45 sec  68 °C 4 min |
| 9 | SPLC1_  S081620 | Possible two-component hybrid sensor and regulator | AP07850026  FW  AP07850026_RV | GAATTCATGATGCGTATCCCCAAAA  TGGGGATCCTCAAATCAAAGT TTTC | 3,729 | Initial denaturation;  95 °C, 3 min  Cycle 1-30;  95 °C 30 sec  50 °C 45 sec  68 °C 4 min |
| 10 | SPLC1_  S360070 | Two-component hybrid sensor and regulator | AP07650006  FW  AP07650006_RV | GAATTCGTGCATCAGGATGTAGTC C  TGGGGATCCTTAAATGGTCAAATT TGC | 3,945 | Initial denaturation;  95 °C, 3 min  Cycle 1-30;  95 °C 30 sec  50 °C 45 sec  68 °C 4 min |
| 11 | SPLC1_  S203560 | Similar multi-sensor signal transduction histidine kinase | AP06710002  FW  AP06710002_RV | GAATTCATGGCTTCCACCGATATC AAT  TGGGGATCCTTAATGGTCAGTACGG | 3,033 | Initial denaturation;  95 °C, 3 min  Cycle 1-30;  95 °C 30 sec  50 °C 45 sec  68 °C 3 min |
| 12 | SPLC1_  S270460 | Elongation factor Tu (EfTu) | AP06740003FW  AP06740003RV | GGAATTCATGACATTGGCTGCCTC T  TTGATCCCTAAGCCAGAATCTTAG CA | 1,230 | Initial denaturation;  95 °C, 3 min  Cycle 1-30;  95 °C 30 sec  50 °C 45 sec  68 °C 2 min |
| 13 | SPLC1_  S032670 | Similar two-component sensor histidine kinase | AP95690002  FW  AP95690002  RV | GGAATTCATGTTCCAAGCTACTCGA AG  CGGGATCCTCAATTACTTTGTTGCAG | 1,512 | Initial denaturation;  95 °C, 3 min  Cycle 1-30;  95 °C 30 sec  50 °C 45 sec  68 °C 2 min |
| 14 | SPLC1_ S270380 | Glutamate synthase (GlsF) | AP06740013_FW  AP06740013_RV | CATATGATGGATAGAAAAGAAACCAAT TTC A  GGATCCCTACACCGC AACCTTT T | 4,704 | Initial denaturation; 95 °C, 3 min  Cycle 1-30;  95 °C 30 sec  50 °C 45 sec  72°C 5 min |
| 15 | SPLC1_ 270380Y237A | Glutamate synthase (Mutant Y237A) | SPL270380_ Y237AFW  SPL270380_ Y237ARV | GACTATTGTTGCCAA GGGTATGGTGC    CGACAGGACCAGGAGCAAATGTAGAT | 4,704 | Initial denaturation; 98 °C, 3 min  Cycle 1-30;  98 °C 30 sec  65 °C 45 sec  72 °C 4 min |
| 16 | Upstream of sll0474 | Upstream of Sensory transduction histidine kinase Hik28 | US-sll0474  ERIFW  US-sll0474  HdIIIRV | CGGAATTCCACAGCCGCTCATAGTAGG  CCCAAGCTTTCTTAAGACCGCTCCACA | 900 | Initial denaturation;  95 °C, 3 min  Cycle 1-30;  95 °C 30 sec  50 °C 45 sec  68 °C 1 min |
| 17 | Downstream of sll0474 | Downstream of Sensory transduction histidine kinase Hik28 | DS-sll0474  KpnIFW  DS-sll0474  pstIRV | TTGGTACCCGACGAGAACCATCGGGCA  GGGCTGCAGAAATTGAGCCTGAATCGGTTC | 900 | Initial denaturation;  95 °C, 3 min  Cycle 1-30;  95 °C 30 sec  50 °C 45 sec  68 °C 1 min |
| 18 | sll0474 | Sensory transduction histidine kinase Hik28 | Sll0474ERI  FW  Sll0474BaHI  RV | CCGGAATTCATGGTTCAGGAGAAACCACGT  TTGGATCCTTAACGGGGAACGGGA CAAG | 2,408 | Initial denaturation;  95 °C, 3 min  Cycle 1-30;  95 °C 30 sec  50 °C 45 sec  68 °C 3 min |
