# Supplementary material for: Revealing the key point of the temperature stress response of Arthrospira platensis C1 at the interconnection of C- and N- metabolism by proteome analyses and PPI networking
Source: BMC Mol Cell Biol. 2020 Jun 12;21:43. doi: 10.1186/s12860-020-00285-y (PMC7291507; doi:10.1186/s12860-020-00285-y)

**Additional file 19**

pGBKT7 Vector (Clontech Laboratories, Inc. USA)


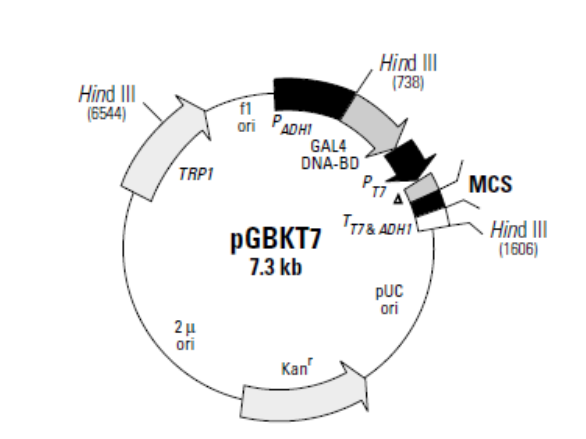


pGADT7 AD Vector (Clontech Laboratories, Inc. USA)


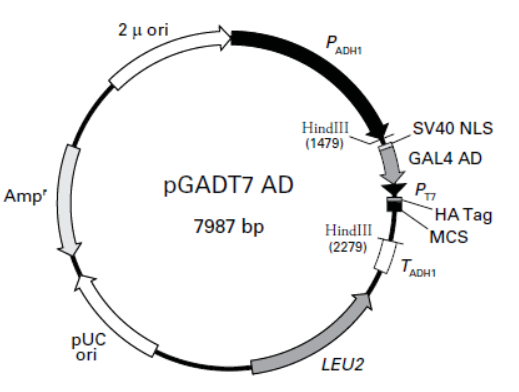

Supplement: Supplementary file 19 — Additional file 19. Pictures of pGBKT7 and pGADT7 AD vectors (Clontech Laboratories, Inc. USA). [file 12860_2020_285_MOESM19_ESM.docx]
